# Valuation of opportunity costs by rats working for rewarding electrical brain stimulation

**DOI:** 10.1371/journal.pone.0182120

**Published:** 2017-08-25

**Authors:** Rebecca Brana Solomon, Kent Conover, Peter Shizgal

**Affiliations:** Centre for Studies in Behavioural Neurobiology / Groupe de recherche en neurobiologie comportementale, Department of Psychology, Concordia University, Montréal, Québec, Canada; Brain and Spine Institute (ICM), FRANCE

## Abstract

Pursuit of one goal typically precludes simultaneous pursuit of another. Thus, each exclusive activity entails an “opportunity cost:” the forgone benefits from the next-best activity eschewed. The present experiment estimates, in laboratory rats, the function that maps objective opportunity costs into subjective ones. In an operant chamber, rewarding electrical brain stimulation was delivered when the cumulative time a lever had been depressed reached a criterion duration. The value of the activities forgone during this duration is the opportunity cost of the electrical reward. We determined which of four functions best describes how objective opportunity costs, expressed as the required duration of lever depression, are translated into their subjective equivalents. The simplest account is the identity function, which equates subjective and objective opportunity costs. A variant of this function called the “sigmoidal-slope function,” converges on the identity function at longer durations but deviates from it at shorter durations. The sigmoidal-slope function has the form of a hockey stick. The flat “blade” denotes a range over which opportunity costs are subjectively equivalent; these durations are too short to allow substitution of more beneficial activities. The blade extends into an upward-curving portion over which costs become discriminable and finally into the straight “handle,” over which objective and subjective costs match. The two remaining functions are based on hyperbolic and exponential temporal discounting, respectively. The results are best described by the sigmoidal-slope function. That this is so suggests that different principles of intertemporal choice are involved in the evaluation of time spent working for a reward or waiting for its delivery. The subjective opportunity-cost function plays a key role in the evaluation and selection of goals. An accurate description of its form and parameters is essential to successful modeling and prediction of instrumental performance and reward-related decision making.

## Introduction

### Opportunity cost

*“Lost time is not found again”*—Bob Dylan, Odds and Ends

Time is the quintessentially scarce good [1, 2]. Its supply cannot be increased, no matter how much we might wish otherwise, and its quantity is insufficient for the full satisfaction of our needs and wants. Time invested in reaping a particular benefit could have been spent instead in pursuit and enjoyment of another. By choosing to invest time towards attainment of one goal, we forgo the benefits of alternatives that cannot be pursued simultaneously. Thus, consumption of time is one of the fundamental costs of any activity, one that must be taken into account by any mechanism for optimizing the selection of goals and actions. Economists use the term, “opportunity cost,” to refer to the foregone benefits of the most valuable activity that was eschewed in favour of the one pursued [3, 4]. This concept also plays an important role in accounts of the behavior of non-human animals [2]. For example, time spent foraging for food reduces time available for other activities essential for survival [5, 6].

The present paper concerns the psychophysical function that translates the objective time spent pursuing a reward into a subjective opportunity cost. Such psychophysical functions figure prominently in accounts of perception and decision making. For example, the functions that transform objective gains and losses into changes in subjective value and that transform objective probabilities into subjective decision weights lie at the core of prospect theory [7, 8], arguably the most influential single account of human decision making. Psychophysical transformation of value plays a similarly important role in accounts of reward-seeking decisions in laboratory animals [9–13]. We show that like the functions at the core of prospect theory, the function that evaluates opportunity costs is non-linear and has a form that is consequential for reward-seeking decisions.

We investigated evaluation of opportunity costs by rats working for rewarding electrical stimulation of the medial forebrain bundle (MFB). Performance for such stimulation is remarkably stable over time and is not undermined by accumulating satiety, as is the case for natural rewards, such as food. This makes it feasible to run long test sessions during which many rewards are collected under near-constant conditions, thus facilitating the collection of the large datasets required to describe the subjective mapping of opportunity costs. Multiple models of this function were tested. Determining which of these is best has implications for accounts of inter-temporal choice. For instance, the tested functions provide different answers to the question of whether time spent working to earn a reward, which imposes an opportunity cost, is evaluated in the same way as time spent waiting for delivery of a reward that has already been earned, which drives delay discounting.

This experiment also has implications for efforts to model, distinguish, and characterize neural processes underlying reward pursuit. One such effort, Shizgal’s “reward-mountain” model, relates reward-seeking behavior to the magnitude, cost, and risk of the returns. In early versions of this model, the objective opportunity cost contributes to valuation directly [14–17], whereas in later implementations and applications [18–21], objective opportunity costs undergo psychophysical transformation. The present paper pits the psychophysical function adopted in that work against three alternatives.

### The schedule of reinforcement

In the present work, electrical stimulation of the MFB sets the strength of the reward, whereas a novel schedule of reinforcement sets the opportunity cost. In operant experiments, the contingency between responses and reward delivery is determined typically by ratio or interval schedules of reinforcement. Conover and Shizgal [22] argue that both of these schedule types fail to control the opportunity cost stringently; the subject retains partial control. To remove control of opportunity costs from the subject and grant it exclusively to the experimenter, we have developed a novel schedule of reinforcement called the “fixed, cumulative, handling-time schedule” (FCHT schedule) [15, 23]. On this schedule, the rat accumulates work time by holding down a lever. A reward is delivered when the cumulative time that the rat has depressed the lever (“work time”) reaches a criterion duration. The accumulation of hold time pauses when the lever is released but increments again when the lever is next depressed.

We refer to the criterion duration of accumulated work time as the “objective price” of the reward, objective in the sense that it is a physical quantity determined by the experimenter. This quantity is controlled strictly on the FCHT schedule. Unlike the case of ratio schedules, the subject has no control over the minimum inter-reward interval, and unlike the case of interval schedules, all work performed by the subject is credited towards earning the next reward.

Holding down the lever precludes engagement in other activities, such as grooming, resting and exploring. Thus, the rewards from these alternate activities are foregone when the subject works. In a manner consistent with economic accounts [3, 4], we equate the foregone benefits with the opportunity cost of pursuing the reward offered by the experimenter.

Performance for rewarding electrical brain stimulation on the FCHT schedule provides a tightly controlled, stable, and simplified context in which to study the contribution of opportunity costs to reward-seeking behavior. For a foraging animal in a natural setting, opportunity costs are composed of multiple components associated with search, procurement, handling, and consumption. Substitution of brain-stimulation reward (BSR) for a natural reward and imposition of the FCHT schedule collapses the components of the opportunity cost into one variable: the required cumulative work time (objective price). Manipulation of this variable changes, in an orderly fashion, the proportion of the trial time spent working for BSR on the FCHT schedule (“time allocation”) [15, 18]. This makes it possible to estimate the subjective valuation of opportunity costs with considerable precision. The inference of subjective opportunity cost from performance on the FCHT schedule is described in the following section.

### The dependence of FCHT performance on the balance between benefits and costs

The reward-mountain model [15, 18] expresses the payoff from work performed on the FCHT schedule in terms of the balance between benefits and costs. In the present experiment, the benefit arises from the neural signal triggered by the MFB stimulation, which is presumed to simulate the benefit derived from naturally incurring rewards [24–27]. This benefit is discounted by the product of two types of costs, one stemming from the time devoted to work (the opportunity cost) and the other from the exertion entailed in holding down the lever (the effort cost):

$$\begin{array}{c} U_{b}=\frac{R}{\left(1+\xi \right)\times P_{sub}} \end{array}$$ (1)

where

*P_sub_* = subjective opportunity cost

*R* = intensity of the subjective reward signal triggered by the brain stimulation

*U_b_* = payoff from the brain stimulation, and

(1 + *ξ*) = subjective rate of exertion experienced while holding down the lever

We control *R* by setting the pulse frequency of the electrical stimulation [15, 18], and we assume that subjective exertion is constant under the conditions of the experiment. If so, we can infer *P*_*sub*_ if we can estimate *U*_*b*_, which we do by means of a behavioral-allocation function [15, 18] derived from the single-operant matching law [28–30]:

$$\begin{array}{c} T=T_{min}+[\left(T_{max}-T_{min}\right)\times \frac{U_{b}^{\;a}}{U_{b}^{\;a}+U_{e}^{\;a}}] \end{array}$$ (2)

where

*a* = the payoff-sensitivity exponent

*T* = the proportion of trial time spent holding down the lever (“time allocation”)

*T_max_* = maximal time allocation

*T_min_* = maximal time allocation

*U_b_* = payoff from a train of rewarding brain stimulation, and

*U_e_* = payoff from leisure activities

Thus, Eqs 1 and 2 link the quantity we wish to infer, the subjective opportunity cost of the reward (*P*_*sub*_) to a quantity we observe: the proportion of trial time that the rat devotes to work (*T*).

Symbols and acronyms are listed in Table 1.

**Table 1:** Definition of symbols and acronyms.

Symbol or acronym	Units	Meaning
*AIC*		Akaike information criterion
*a*		price-sensitivity exponent
*BSR*		brain stimulation reward
*D*	*s*	delay to reinforcement
*F*	*Hz*	pulse frequency
*F*_*hm*_	*Hz*	pulse frequency at which reward intensity is half-maximal
*F*_*mid*_	*Hz*	pulse frequency at which time allocation is mid-way between its minimum and maximum values
*FCHT*		Fixed-cumulative-handling-time
*f*_*t*_		interval-timing function
$f_{P_{sub}}$		subjective-price function
*f*_*v*_		time-valuation function
*g*		reward-growth exponent
*K*_*h*_		delay-discount constant
*K*_*u*_	*utils* *hedon*^−1^	constant that translates reward intensity into payoff
*K*_*x*_		exponent of exponential-price function
*P*_*obj*_	*s*	objective price (set by the experimenter)
*P*_*obj*_*e*_	*s*	objective price (set by the experimenter) at which time allocation to pursuit of a maximal reward is half-way between its minimum and maximum values
$P_{obj_{mid}}$	*s*	For a given value of *F*, the price that drives *T* half-way between its minimum and maximum values
*P*_*sub*_	*s* *oomph*^−1^	subjective price
$P_{sub_{bend}}$		transition parameter of sigmoidal-slope function
*P*_*sub*_*e*_	*s* *oomph*^−1^	subjective price at which time allocation to pursuit of a maximal reward is half-way between its minimum and maximum values
$P_{sub_{mid}}$	*s* *oomph*^−1^	
$P_{sub_{min}}$	*s* *oomph*^−1^	minimum subjective price
*R*	*hedon*	reward intensity
*R*_*rel*_		reward intensity expressed as a proportion of its maximal value
*R*_*max*_	*hedon*	maximal reward intensity
*S*	*hedon*	reward magnitude
*T*		time allocation
*T*_*max*_		maximal time allocation
*T*_*mid*_		time allocation half-way between its minimum and maximum values
*T*_*min*_		minimal time allocation
*t*_*sub*_	*s*	subjective time
*U*_*b*_	*util*	utility of brain stimulation reward
*U*_*e*_	*util*	utility of alternate activities (everything else)
*V*		delay-discounted reward value
*ξ*	*oomph* *s*^−1^	constant determining the rate of subjective exertion

See S1 File for discussion and definition of units.

### The translation of objective price into subjective opportunity cost

The purpose of this study is to describe and explain the “subjective-price function” used by the subject to evaluate the objective price of the reward. At least two stages are involved. The first entails estimation of the criterion work duration, whereas the second entails valuation of the resulting estimates.

Estimation of time intervals has been studied and modeled extensively. In one influential account [31], subjective estimates are directly proportional to objective time intervals, with scalar error and constant coefficient of variation. Formally,

$$\begin{array}{c} t_{sub}=f_{t}\left(P_{obj}\right) \end{array}$$ (3)

where

*t_sub_* = the subjective time estimate

*P_obj_* = the objective price

*f_t_* = the function that translates the objective price set by the experimenter into a subjective time estimate. In Gibbon’s theory, *f_t_* is scalar.

The subjective estimate of the time worked is converted into a subjective-price estimate by a second function, *f*_*v*_:

$$\begin{array}{c} P_{sub}=f_{v}\left(t_{sub}\right) \end{array}$$ (4)

where

*f_v_* = the function that valuates *t_sub_*, thus translating it into a subjective opportunity cost

The units of *P*_*sub*_ are defined in S1 File.

Substituting for *t*_*sub*_, we obtain:

$$\begin{array}{c} P_{sub}=f_{v}\left(f_{t}\left(P_{obj}\right)\right) \end{array}$$ (5)

The subjective-price function is then generated by embedding *f*_*t*_ in *f*_*v*_:

$$\begin{array}{c} P_{sub}=f_{P_{sub}}\left(P_{obj}\right) \end{array}$$ (6)

where

$f_{P_{sub}}$ = the subjective-price function

This composite function ($f_{P_{sub}}$) translates objective work times (the price of the reward, *P*_*obj*_) into subjective opportunity costs (*P*_*sub*_). In the following section, we discuss the four candidate forms for this function that will be assessed in the present study.

### Competing candidates for the subjective-price function

#### Objective-price function

The simplest form that the subjective-price function can assume is the identity function. Formally,

$$\begin{array}{c} P_{sub}=P_{obj} \end{array}$$ (7)

Estimation of time intervals is a noisy process [31]; additional noise would be expected in valuation of the associated opportunity cost. Thus *P*_*sub*_ in Eq 7 should be interpreted as the central tendency of a distribution of estimates. In addition, the subjective-price function cannot extend indefinitely. There is only so long an animal can work for a reward and still manage to survive, given that energy stores are finite [33]. In practice, there is a limit on the testing-session duration during which performance for rewarding brain stimulation remains stable and a much lower limit on the highest price that the rat will pay for the reward. The highest price tested in the current study was 238.5 s.

**Fig 1:**
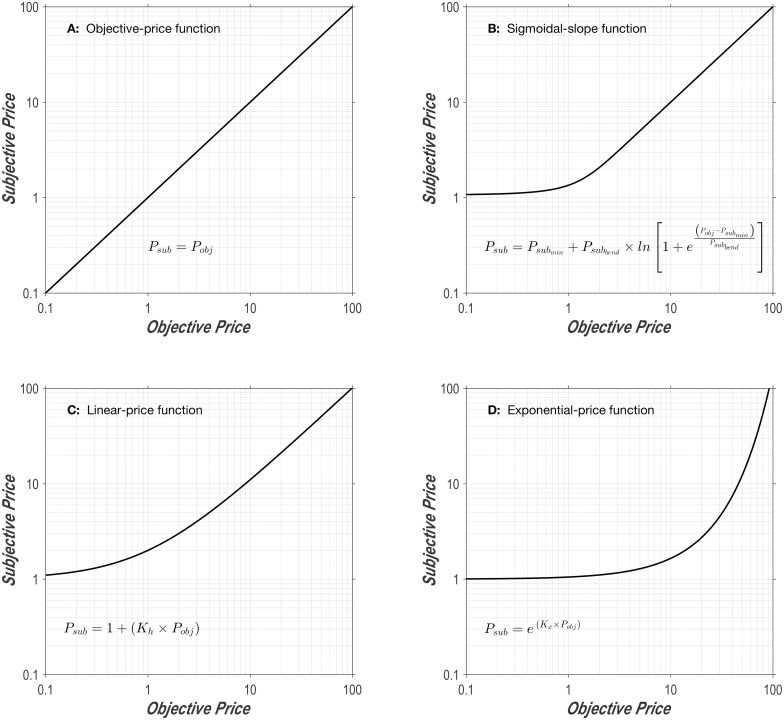
**A: Objective-price function.** Subjective prices are equal to objective prices, as assumed in early studies using the reward-mountain model [14–17]. **B: Sigmoidal-slope function.** The functional form resembles a hockey stick. The flat “blade” of the function denotes the range over which costs are subjectively invariant. That portion merges with an upward-bending segment over which the costs are discriminable, but do not yet rise at the same rate as the objective price. The final “handle” portion denotes the range over which the relationship between objective and subjective costs is scalar. Changing the value of the $P_{sub_{min}}$ parameter shifts the curve vertically, whereas changing the value of the $P_{sub_{bend}}$ parameter alters the abruptness of the transition between the blade and the handle. **C: Linear-price function.** This is the subjective-price function derived from Mazur’s [32] hyperbolic-discounting equation. The greater the value of *K*_*h*_, the faster the function rises. In order for the terminal slope to approach unity, *K*_*h*_ must equal 1. (The lines look curved because they are plotted in double logarithmic coordinates; in linear coordinates, they would be straight.) **D: Exponential-price function.** This is the subjective-price function derived from exponential discounting. The larger the *K*_*x*_ value, the more rapidly the function rises. See S1 File for a discussion of units.

The plot in Fig 1**A** shows the objective-price function in double logarithmic coordinates. This function becomes increasingly unrealistic as the duration of the required hold time (the price) decreases. For example, according to the function portrayed in Fig 1**A**, the benefit/cost ratio (“payoff”) from a reward of given strength that requires 0.10 s of work to obtain is one half the payoff from that reward when only 0.05 s of work are required. However, reducing the objective price from 0.10 s to 0.05 s is unlikely to be significant to the animal because the 50 ms thus freed up are insufficient to perform a worthwhile alternate activity. The realization that opportunity costs cease to matter below some minimal duration is what drove us to modify the objective-price function in the manner described in the next section.

#### Sigmoidal-slope function

We propose that the subjective-price function is flat over the shortest work times, rises at the same rate as the objective price once work times become substantial, and transitions smoothly between these two segments (Fig 1**B**). The proposed function thus has the form of a hockey stick, with a flat blade that curves into a straight, diagonally oriented handle; the handle has a slope of 1 when plotted in double logarithmic coordinates. Note that the handle corresponds to the objective-price function, whereas the blade and curved connecting region deviate from it. The integral of a sigmoid has the required form:

$$P_{sub}\;=\;\int \frac{1}{1+{\text{e}}^{\left(\frac{P_{sub_{min}}\;-\;P_{obj}}{P_{sub_{bend}}}\right)}}\text{d}P_{obj}$$ (8)

=Psubmin+Psubbend×ln(1+ePobj-Psubmin(Psubbend() (9)

where

$p_{sub_{min}}$ = the minimum subjective price and

$p_{sub_{bend}}$ = a constant that controls the abruptness of the transition. from “blade” to “handle”

#### Linear-price function

The rationale for the objective- and sigmoidal-slope functions treats the valuation of work time as distinct from delay discounting. Work time refers to time spent fulfilling the response requirement for procuring a reward, whereas reinforcement delays refer to time spent waiting for reward delivery after the response requirement has already been satisfied. What if these two times were subject to analogous forms of valuation? From that viewpoint, the time that elapses while holding down the lever is construed as a delay between response initiation and reward delivery. Here, we adapt two widely used delay-discounting functions for application to the FCHT paradigm.

In a typical delay-discounting experiment, the agent is offered a choice between performance of two responses that deliver rewards of different magnitudes after delays of different duration [34, 35]. Alternatively, the agent may be offered an implicit choice [28, 29] between performance of a single response that triggers a delayed, experimenter-controlled reward and performance of alternate activities such as resting, exploring or grooming [36, 37]. In either case, the value of the prospective reward at the time of response selection declines as the expected time of reward delivery recedes into the future. To apply this delay-discounting perspective to performance on the FCHT schedule, this delay-driven decline in value is recast as a cost of waiting. This requires that the delay-discounting function be inverted. (Value declines as a function of delay, whereas the cost of waiting grows.)

Behavioral data obtained from many types of experimental subjects and in many delay-discounting paradigms are well described by Mazur’s hyperbolic delay-discounting equation [32]:

$$\begin{array}{c} V=\frac{S}{1+(K_{h}\times D)} \end{array}$$ (10)

where

*V* = discounted subjective value of the future reward

*S* = a constant representing reward magnitude

*K_h_* = a constant that determines how abruptly the value of the function declines as the delay grows, and

*D* = the delay in seconds from the completion of the response requirement to delivery of the reward

Inversion transforms Mazur’s equation from a rectangular hyperbola into a straight line:

$$\begin{array}{c} \frac{1}{V}=\frac{1}{S}\times [1+(K_{h}\times D)] \end{array}$$ (11)

The *S* parameter represents the magnitude of the reward (e.g., the number of food pellets). The reward-mountain model that we have fit to the data already includes a variable, “subjective reward intensity,” that replaces Mazur’s *S* parameter [14, 15, 18]. Thus, we remove the scale parameter from Eq 11 in order to arrive at an expression for the subjective price of the reward. Given that the required work time is to be treated as a delay to reward delivery, we substitute the objective price (*P*_*obj*_) for *D* in Eq 11; to describe the escalating cost of waiting for reward, we equate the inverted, delay-discounted value ($\frac{1}{V}$) in Eq 11 to the subjective price (*P*_*sub*_). The resulting equation thus expresses the subjective price of holding down the lever as a function of the required work time in a manner analogous to hyperbolic discounting of reward as a function of the delay to reward delivery:

$$\begin{array}{c} P_{sub}=1+\left(K_{h}\times P_{obj}\right) \end{array}$$ (12)

We call Eq (12) the “linear-price function.” Note that this function differs from the objective-price function only by an additive constant (the y-intercept, which is equal to one) and a slope constant. The additive constant nonetheless produces a substantial change in the shape of the function in the double-logarithmic plot (Fig 1**C**) and causes the linear-price function to deviate from the objective-price function in the general manner we have advocated, which blunts the impact of changes over the lowest range of prices.

The linear-price function has only one parameter in contrast to the sigmoidal-slope function, which has two. Thus, the linear-price function is less flexible than the sigmoidal-slope function but faces less stringent criteria in model-fitting comparisons, which prize simplicity and penalize the addition of parameters. The sigmoidal-slope function can transition more abruptly from blade to handle; both blade and handle are truly straight, whereas they are only approximately so in the case of the double-logarithmic plot of the linear-price function. In order for the linear-price function to behave normatively at larger objective prices, *K*_*h*_ must equal one. However, that value prevents the function from approximating a flat blade at low prices. Lower values of *K*_*h*_ (e.g., 0.1) can provide good approximations of a flat blade, but at the cost of dramatically compressing the subjective-price values; for example, when *K*_*h*_ = 0.1, subjective prices rise at only 10% of the rate of rise in the objective prices.

#### Exponential-price function

The final function evaluated in this study is derived from another discounting function, one that has been used widely in economics [38] and machine learning [39]. Just as inversion changes the Mazur equation into the linear-price function, inversion (changing the sign of the exponent) transforms the exponential-discounting function into an exponential-price function:

$$\begin{array}{c} P_{sub}=e^{K_{x}\times P_{obj}} \end{array}$$ (13)

where

*K_x_* = a constant that controls the rate at which subjective price grows

The exponential-price function is illustrated in Fig 1**D**. Like the linear-price function, it has only a single parameter. Unlike both the linear-price function and the sigmoidal-slope function, the exponential-price function cannot have a straight or approximately straight handle. Like the linear-price function and unlike the sigmoidal-slope function, the exponential-price function cannot have a truly flat blade.

In principle, any number of additional functions could have been tested, although in practice, time and resource constraints dictated that we restrict the number of candidates. For simplicity, we have confined ourselves to four: the simplest possible function (the objective-price function), a modification of this simple function to deal more realistically with low prices (the sigmoidal-slope function), and functions derived from the two most common expressions for temporal discounting (the linear- and exponential-price functions). Even this restricted test allows us to determine how the subjective-price function employed in the most recent version of reward-mountain model [18] fares in competition with plausible alternates. In that work, the objective-price function was replaced by the sigmoidal-slope function on the basis of *a priori* assumptions. Here we determine empirically whether the sigmoidal-slope function in fact provides a better description of the data.

#### Measuring the subjective-price function

**Fig 2:**
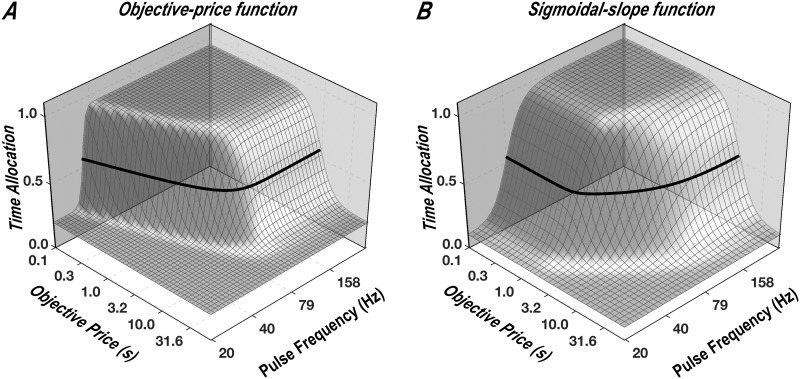
The reward mountain. Allocation of time to reward seeking as a function of the cost and strength of reward. **A**: The surface predicted by the version of the reward-mountain model incorporating the objective-price function. **B**: The surface predicted by the version of the reward-mountain model incorporating the sigmoidal-slope function. The heavy black line traces the contour corresponding to “mid-range” time allocation (half-way between the minimal and maximal values). Given that the two surfaces differ in shape, we can choose between the subjective-price functions embedded in the respective models (and in the linear-price and exponential-price functions as well) by determining which surface best fits the data. Note that the x-axes represent *P*_*obj*_, the objective price. The subjective-price functions determine how *P*_*obj*_ is translated into *P*_*sub*_, the subjective price of the reward. The surfaces produced by the version of the reward-mountain model incorporating the linear- and exponential-price models can be seen in Fig 5**C** and 5**D**.

In this study, we set out to obtain data of sufficient density and precision to determine which of the four functions best accounts for the effect of varying opportunity cost. We used an experimental paradigm for quantitative measurement of reward seeking: operant performance on the FCHT schedule in rats working for rewarding electrical stimulation of the MFB. This paradigm is based on a model [14, 15, 18] that describes how reward seeking depends on the cost and strength of rewards. The model generates a curved surface enclosing what is called the “reward mountain,” which is depicted in Fig 2. A separate version of the model was generated using each of the price-mapping functions described above (Eqs 7, 9, 12 and 13); panels *A* and *B* of Fig 2 show the surfaces generated by the reward-mountain models incorporating the objective-price and sigmoidal-slope functions, respectively. As the contrast between panels *A* and *B* illustrates, the different subjective-price functions generate surfaces with different shapes. This allows us to determine which subjective-price function provides the best fit to the data.

The reward-mountain surfaces shown in Fig 2 are defined by the following equation:

$$\begin{array}{c} T=T_{min}+\left[\left(T_{max}-T_{min}\right)\times \frac{{\left(\frac{F^{g}}{F^{g}+F_{hm}^{g}}\right)}^{a}}{{\left(\frac{F^{g}}{F^{g}+F_{hm}^{g}}\right)}^{a}+{\left(\frac{P_{sub}\left(P_{obj}\right)}{P_{sub\_e}\left(P_{obj\_e}\right)}\right)}^{a}}\right] \end{array}$$ (14)

where

*a* = the payoff-sensitivity exponent. This parameter determines the steepness of the mountain along the price axis.

*F_hm_* = the pulse frequency that produces half-maximal reward intensity. This parameter determines the location of the reward mountain along the pulse-frequency axis.

*g* = the intensity-growth exponent. This parameter determines the steepness of the intensity-growth function and contributes to the steepness of the mountain along the pulsefrequency axis.

*P_obj_* = the objective price (opportunity cost).

*P_obj_e_* = the objective price at which the time allocation to pursuit of a maximal reward falls halfway between *T_max_* and *T_min_*. This parameter determines the location of the reward mountain along the price axis.

*P_sub_*(*P_obj_*) = one of the four subjective price functions defined by Eqs 7, 9, 12 & 13. These functions translate the objective prices (*P_obj_*) represented on the x-axes of Fig 2**A** and 2**B** into subjective prices (*P_sub_*).

*P_sub_e_*(*P_obj_e_*) = the subjective price at which time allocation to pursuit of maximal reward falls halfway between *T_max_* and *T_min_*. This constant is obtained by passing *P_obj_e_* through a subjective-price function.

*T* = time allocation.

*T_max_* = maximal time allocation.

*T_min_* = minimal time allocation.

With reference to Eq 14, we can define the objective of this study as finding the form of the subjective-price function (the mapping of *P*_*obj*_ into *P*_*sub*_) that provides the best and most realistic fit of the surface to the data.

In prior experiments [14–21], the surface defined by Eq 14 has been estimated by sampling sparsely from the independent-variable space so as to obtain sufficient data to fit the model accurately while minimizing the required testing time. The space represented by the cost and strength of reward (Fig 2) is traversed along three paths, one parallel to the strength axis (the pulse-frequency “pseudo-sweep”), one parallel to the cost axis (the price pseudo-sweep), and one running diagonally from the intersection of the pulse-frequency and price pseudo-sweeps (the radial pseudo-sweep). To estimate the subjective-price function in the present study, we added frequency pseudo-sweeps at equally spaced intervals along the logarithmic objective-price axis. Different versions of the reward-mountain model were then fitted to the data, each incorporating one of the subjective-price functions described above (Eqs 7, 9, 12 and 13), and the goodness of fit was assessed by means of the Akaike Information Criterion (AIC) [40].

## Methods

### Subjects

Animal-care and experimental procedures were carried out in accordance with the principles in the Canadian Council on Animal Care’s (CCAC) Guide to the Care and Use of Experimental Animals, with the approval of the Concordia University Animal Research Ethics Committee (certificate #: 30000302). Six male Long-Evans rats (Charles River breeding farms, St. Constant, Québec, Canada) that weighed between 450 g and 600 g at the time of surgery served as subjects. They were housed individually in plastic cages and had unlimited access to food (Purina Rat Chow) and water. A reverse light cycle was in effect (lights off from 08:00 to 20:00). The rats served as subjects in a previous experiment [21] in which the current was varied and the frequency-following function was estimated. The subjective-price experiment was conducted prior to the frequency-following experiment.

### Surgical procedure

Prior to surgery, a subcutaneous injection of atropine sulfate (0.05 mg/kg, sc) was given to reduce bronchial secretions. Ten minutes later, a ketamine-xylazine mixture (10/100 mg/kg, ip) was administered to induce anesthesia. To confirm that the level of anesthesia was sufficiently deep, the tail was pinched 5 min after administration of the ketamine-xylazine mixture. Buprenorphine (0.05 mg/kg, sc) was administered as an analgesic, and penicillin-g (0.3 ml, sc) was administered to prevent infections. Xylocaine jelly was applied in the ears to prevent discomfort from the stereotaxic ear bars. The anesthetized rat was mounted into a stereotaxic frame. Anesthesia was maintained during the remainder of the surgical procedure by administration of isoflurane through a snout-mounted mask affixed to the stereotaxic frame.

Six jeweller’s screws embedded in the frontal and parietal bones served as anchors for the electrode assembly. A 5 cm thread of copper wire was wrapped around two of the skull screws, which served as the anode for the stimulation circuit. The other end of this wire was crimped to a male Amphenol pin. A monopolar stimulating electrode (0.25 mm in diameter), fashioned from a 000 stainless steel insect pin and insulated with Formvar to within 0.5 mm of the tip, served as the cathode. An insulated wire was soldered to the insect pin and terminated in a gold-plated male Amphenol pin. The stimulation electrodes were lowered into place using standard stereotaxic manipulators, aimed at the lateral hypothalamus of the left hemisphere with reference to the Paxinos and Watson atlas [41] (AP: -2.8 mm from bregma, ML: -1.7 mm from the midline, DV: 9.0 mm from the skull surface). Dental acrylic was used to secure the electrode and connector to the skull and jeweller’s screws. The Amphenol pins were inserted into an externally threaded, nine-pin connector (Scientific Technology Centre, Carleton University, Ottawa, Ontario, Canada). Rats were given a 1-week recovery period after surgery to allow healing before preliminary testing began.

### Apparatus and materials

The stimulation lead was attached to an electrical swivel at the top of the test box, allowing the rat to circle without becoming tangled in the lead. A second cable linked the swivel to the output of the constant-current stimulator.

The test boxes were 34 cm × 23 cm × 60 cm with Plexiglas walls and a hinged Plexiglas front door. Two retractable levers (1.5 cm × 5 cm) (ENV–112B, MED Associates, St. Albans, Vermont) were located in the center of the right and left walls, 10 cm above the wire-mesh floor; only the right lever was used in the present experiment. A cue light, located 2 cm above the lever, was illuminated when the lever was depressed. A house light, located on the back wall 35 cm from the floor, flashed between trials.

### General summary of the behavioral testing procedures

The subjects underwent 3 phases of behavioral testing:

1. preliminary testing
2. training (frequency sweeps, price sweeps, 3D sampling)
3. the subjective price experiment proper (estimation of the subjective-price function).

Performance for rewarding brain stimulation was measured as a function of two independent variables (referred to as the experimental parameters): pulse frequency and objective price. A trial is defined as a period within a daily session during which the values of these two independent variables remain fixed. The value of one or more of these variables was changed from trial to trial, but remained fixed within a trial. The composition and duration of the trials within the test sessions at each phase of testing are described in detail below. Throughout the experiment, stimulation consisted of 0.5 s trains of rectangular, cathodal, constant-current pulses, 0.1 ms in duration.

### Detailed description of the behavioral protocol

#### Preliminary testing

Preliminary testing using manually controlled stimulators allowed the experimenter to determine whether the stimulation electrode was effective (screening) and to determine the values of the independent variables (pulse frequency and current) supporting maximal response rates for each rat. An FR-1 schedule of reinforcement was in effect. At first, weak stimulation trains (low current and pulse frequency) were delivered. The stimulation current was increased gradually if the rat failed to approach the lever and no signs of aversion or motor-effects were observed. The rat was trained to press the lever using standard shaping techniques. Once shaped, subjects were transferred to an automated operant set-up controlled by a customized program named “PREF” developed by Steve Cabilio (Concordia University, Montréal, Québec, Canada). The subsequent experimental phases were conducted in the automated setup.

#### Task details

**Schedules of reinforcement.** During all phases of the study following the initial screening, a fixed cumulative handling-time (FCHT) reinforcement schedule was employed. This schedule sets the objective opportunity cost of a stimulation train, which we call the “objective price.” The FCHT imposes the cumulative work time that the rat must invest to harvest the reward [15, 23]. Work time accumulates on a clock as the subject depresses the lever. The accumulation of work time pauses when the lever is released and then resumes incrementing when the lever is next depressed. The reward is delivered when the cumulative work time reaches a criterion duration (the objective price). The schedule name refers to the concept of handling time in behavioral ecology.

Objective prices spanned 0.125 s to 238.5 s. (Because of the PC clock-system constraints, the actual price is not exactly equal to the experimenter-set price, but is very close. The error is somewhat larger at the lower prices but is unlikely to be of great consequence given that the models predict little or no change in subjective price in this range. Table A in S1 File shows the differences between the experimenter-set price and actual (computer-set) price, from 0.125 s to 16 s. For simplicity, we refer to the objective prices by their experimenter-set values.)

**The black-out delay.** The lever was extended at the beginning of the trial and withdrawn for a 2-4 s black-out delay immediately after reward delivery. The duration of this black-out delay was dependent on the properties of the stimulation in each subject. Aversive or motoric side-effects limit time allocation, and thus, the duration of the black-out delay was adjusted in the training sessions so as to minimize this interference. If the maximal time allocation was 0.6 or less when the black-out delay was 2 s in the initial training condition, the black-out delay was increased.

**Trial time and duration.** Trial time was defined as the total duration within a trial during which the lever was extended and depression of the lever incremented the cumulative work time. The trial time during the experiment proper was set to be 25 times the price of the reward, except in the case of prices <1 s, in which case the trial time was 25 s.

**Cue light over lever.** The cue light was illuminated to signal the rat when the lever was depressed sufficiently to achieve a switch closure.

**The inter-trial interval.** The 10 s inter-trial intervals were signalled by flashing the house light.

**Priming stimulation.** During the screening and training on frequency and price sweeps, the pulse frequency of the priming train was set to the same value as in the train that would be triggered by the lever on that particular trial. During training on the 3D sampling procedure and during the estimation of the subjective-price function, a fixed, high pulse-frequency value was used for the priming stimulation at the start of all trials.

**Fig 3:**
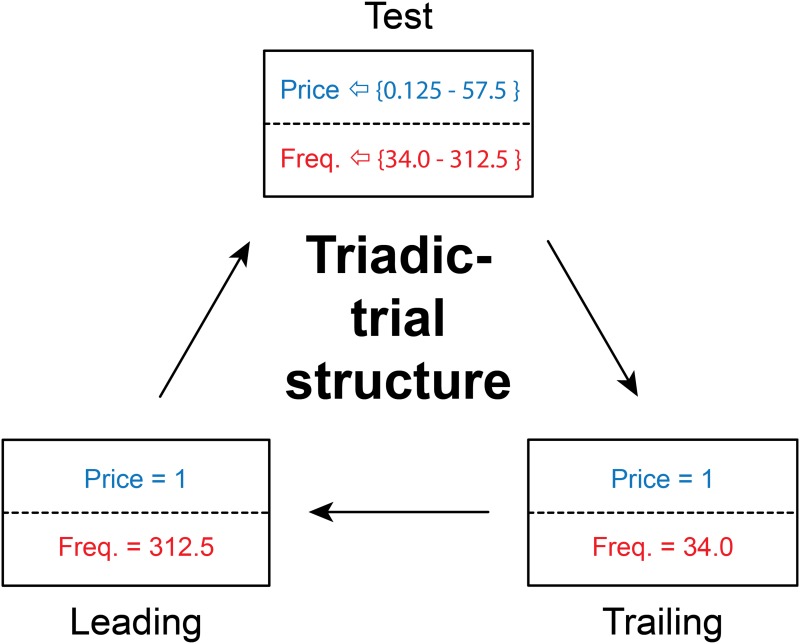
Triadic-trial structure. On test trials, pairs of price and pulse-frequency values are drawn at random from a large set, without replacement; the ranges of these two variables tested in Rat F12 are shown in curly braces. Test trials are interposed between two types of “bracket” trials on which the price of pulse frequency are fixed. On leading-bracket trials, the price is 1 s, and the pulse frequency is set to the maximum value the rat can tolerate without showing signs of aversion or pronounced, forced, stimulation-evoked movements. On trailing-bracket trials, the price is also 1 s, but the pulse frequency is set to a value far too low to support operant responding. The cycle of three trials until every pair of price and pulse-frequency values in the set used on the test trials has been selected, thus completing a “survey” of the reward mountain. The price and pulse-frequency values shown (for Rat F12) are depicted in detail in Fig 4**D**.

**Triadic trial structure.** Due to the possibility that the rat’s standards of evaluation would drift over the course of a test session, a triadic trial structure was employed during training on the 3D sampling procedure and during the main phase of the experiment (when the subjective-price function was estimated). This structure provides repeated exposure to two standardized extremes with which to compare the conditions in effect on the test trial. The triadic trial structure is illustrated in Fig 3. Each experimental trial is embedded between two “bracketing trials.” During the first bracketing trial (the leading trial), the pulse frequency was set to the maximum value a given rat would encounter in the experiment, and the price was 1 s. During the second bracketing trial, (the trailing trial) the pulse frequency was set to a very low value, and the price was 1 s.

#### Sampling matrices of independent-variable values

The experimental parameter space from which we sampled was organized into matrices composed of 2 columns, one for each of the independent variables {*F*: pulse frequency (Hz), *P*_*obj*_: objective price (s)}. (The current remained constant throughout all phases of the experiment.) Three types of sampling matrices were employed:

1. Pulse-frequency sampling matrix: the pulse frequency varies across rows while objective price remains constant. The same set of objective prices was used for all rats. In contrast, the pulse frequencies were selected separately for each rat so as to cause time allocation to grow in sigmoidal fashion as the pulse frequency was increased, with the steeply rising portion of the sigmoid near the center of the pulse-frequency range.
2. Price-sampling matrix: the objective price varies across rows while the pulse frequency remains constant. The pulse frequency, determined separately for each rat, was set to the highest value that the rat could tolerate without showing signs of aversion or excessive stimulation-induced movement. The objective prices were selected separately for each rat so as to cause time allocation to decrease in sigmoidal fashion as the price was increased, with the steeply falling portion of the sigmoid near the center of the price range.
3. Radial-sampling matrix: pulse frequency and objective price co-vary across rows. The pulse frequencies and objective prices were selected separately for each rat so as to cause time allocation to decrease in sigmoidal fashion as the pulse-frequency was decreased and the price was increased, with the steeply falling portion of the sigmoid near the center of the pulse-frequency and price ranges.

**Fig 4:**
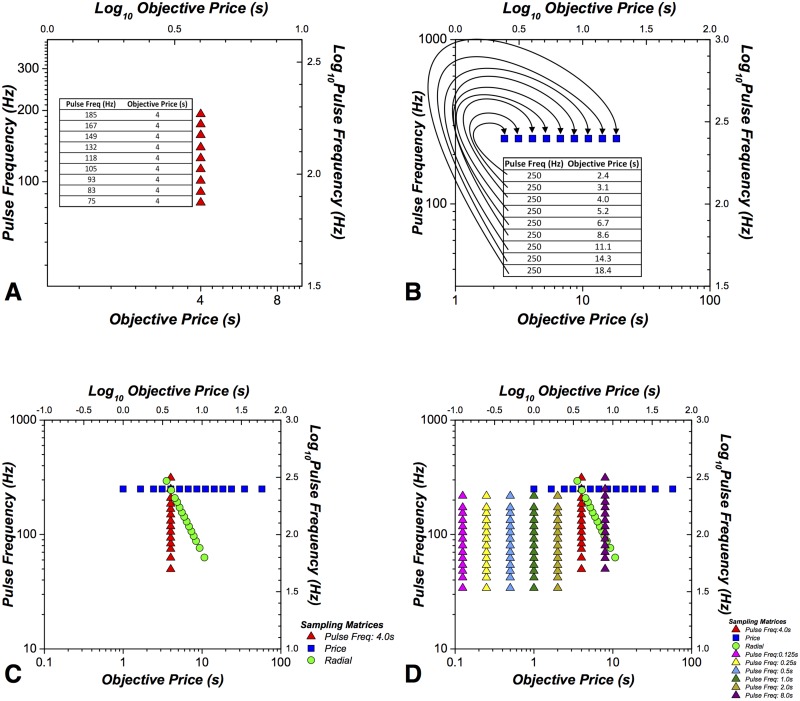
Sampling matrices. **A**. An example of a pulse-frequency sampling matrix. The position of each red marker is determined by the pulse-frequency and price values in the corresponding row of the matrix. The trajectory defined by the set of red markers constitutes a pulse-frequency sweep. **B**. A corresponding example of a price-sampling matrix and price sweep. **C**. The trajectory of a radial pseudo-sweep (green) is shown along with the trajectories of the pulse-frequency (red) and price (blue) pseudo-sweeps. The prefix, pseudo-, refers to the fact that the depicted locations in the parameter space were visited in random order during each experimental session. In contrast, the trajectories shown in **A** and **B** (training on price-frequency and price sweeps) were traversed sequentially. **D**. The full set of pseudo-sweeps used to estimate the subjective-price function. The values shown were used to test Rat F12.

The sampling matrices can be visualized in the 2D experimental parameter spaces: [*Log*_10_(*PulseFrequency*) vs. *Log*_10_(*ObjectivePrice*)]. Each row in a sampling space is denoted by a single point in the parameter space. For example, Fig 4A illustrates a pulse-frequency sampling matrix, Fig 4B illustrates a price-sampling matrix, and Fig 4C illustrates a radial-sampling matrix (green) along with the frequency (red), and price (blue) sampling matrices.

In the example presented in Fig 4A, the price is fixed at 4 s and the pulse frequency at 185 Hz on one trial; on another, the price is fixed at 4 s and the pulse frequency at 167 Hz, etc. In the frequency- and price-sweep training conditions, there were 9 rows in a sampling matrix. In training during 3D sampling and during the main experimental phase (estimation of the subjective-price function), there were 14 rows within each sampling matrix for rats F12, F16, F17 and F18, and 9 rows for rats F3 and F9. In these phases of the study, the experimental parameter values for each trial were drawn from a row of a sampling matrix, without replacement. The experimental phase used to measure the subjective price function employed 1 price-sampling matrix, 7 pulse-frequency sampling matrices, and 1 radial-sampling matrix (Fig 4C).

The trial time was set to allow the rat to harvest a maximum of 25 rewards (e.g., 1 s price × 25 rewards = 25 s trial time, 2 s price × 25 rewards = 50 s, etc.). The black-out delay is excluded from the trial time. A survey is defined as a complete test of all rows of the entire set of sampling matrices employed at a given phase of the study.

#### Training

The 3 training phases served to establish and stabilize task performance as well as to determine the experimental parameter values that drove time allocation from its minimal to maximal values; these parameter values were used subsequently to estimate the subjective-price function. The first two training phases are referred to as “sweeps,” an ascending or descending sequence of experimental parameter values. In the third training phase, the experimental parameter values were sampled randomly from the 3 sampling matrices. The trials carried out using these sampled values were embedded within bracketing trials (i.e., the triadic trial structure was employed).

1. **Frequency sweeps.** Frequency sweeps were carried out by stepping sequentially through the pulse-frequency sampling matrix from row to row. One sweep refers to a test of all rows of the sampling matrix.
  This matrix consists of 9 rows; the objective price remains fixed, whereas the pulse frequency decrements from row to row by equal logarithmic steps (e.g., Fig 4A). For example, if the logarithmic step size was set to 0.05, then the pulse frequencies were: 185, 167, 149, 132, 118, 105, 93, 83, 75 Hz.
  The first trial in the set served as a warm-up trial; this pulse frequency was the same as that of the second trial. The data from the warm-up trial were discarded from the analysis. One daily session consisted of a total of 10 sweeps; the data from the first sweep were discarded from the analysis. In the first frequency-sweep session, the price was set to 1 s. If the maximal time allocation was at least 0.8, the price was raised to 2 s on the next session. If the maximal time allocation was less than 0.8, the current and pulse frequencies were adjusted to drive performance to a time allocation value of at least 0.8. The price was increased until it reached 4 s. The range of pulse frequencies was adjusted such that there were several points along each of the upper and lower asymptotes and along the sloping portion of the psychometric curve relating time allocation to pulse frequency.
2. **Price sweeps.** Following completion of frequency-sweep training, price-sweep training was undertaken by repeatedly stepping sequentially through the price-sampling matrix from row to row. An example of a price-sampling matrix is presented in Fig 4. The pulse frequency was set to the maximum value used in pulse-frequency sweep training and remained fixed while the objective prices were varied by equal, ascending logarithmic steps across 9 rows. For example, at a step size of 0.11 logarithmic units, the tested prices were: 2.4, 3.1, 4, 5.2, 6.7, 8.6, 11.1, 14.3, 18.4 s. An individual sweep consisted of a total of 10 trials: the first trial of the sweep was a warm-up, set to the same price as the one in effect on the second trial. The range and starting price were adjusted such that there were several points on the upper and lower asymptotes and on the sloping portion of the time allocation versus price plot. Because the rats required long trial-time durations at high prices, only 2-3 sweeps were conducted per daily session. In total, the rats trained in this condition for approximately 10-15 daily sessions.
3. **3D sampling.** During this phase of training, a third sampling matrix, the “radial-sampling matrix” (Fig 4C), was introduced along with the triadic trial structure (Fig 3). In frequency- and price-sweep training, the experimental parameters for the “test” trials were chosen from sequential rows of a single sampling matrix. Thus, the value of the “swept” independent variable changed in ascending or descending order. In contrast, during 3D sampling, a row from one of the three matrices {frequency, price, radial} was chosen at random to determine the experimental parameters for the “test” trial. Thus, the values of the independent variables for the “test” trial changed in a random order from triad to triad during 3D sampling.
  In the radial-sampling matrix (green points in Fig 4C), both price and pulse frequency vary from row to row in logarithmic steps. In successive rows, the price increases while the pulse frequency decreases. The trajectory of the pulse-frequency and pulse values in the parameter space [*Log*_10_(*Pulse* *Frequency*) versus *Log*_10_(*Objective* *Price*)] was aimed so as to pass through or near the point defined by the model parameters, [*Log*_10_(*P*_*obj*_*e*_), *Log*_10_(*F*_*hm*_)]. Parameter *P*_*obj*_*e*_ positions the 3D function along the price axis; it is the objective price in seconds that supports half-maximal responding for maximal rewarding electrical stimulation. Parameter *F*_*hm*_ positions the 3D function along the pulse-frequency axis; it is the pulse frequency that produces half-maximal reward intensity. The trajectory through the parameters [*Log*_10_(*P*_*obj*_*e*_), *Log*_10_(*F*_*hm*_)] ensures there is sufficient data to obtain accurate estimates of these critical parameters and an accurate fit of the reward-mountain model. The extremes of the pulse-frequency and price ranges were established on the basis of the data from frequency-sweep and price-sweep training, and the intervening values were generated using a simulator developed by Yannick Breton in MATLAB (The Mathworks, Natick, MA).
  In this phase, the row that determined the experimental parameters for a given trial was sampled at random and without replacement from the following matrices:
  - Pulse-frequency sampling matrix (pulse frequency varies across 9 or 14 values, fixed 4 s price)
  - Price-sampling matrix (objective price varies across 9 or 14 values, fixed high pulse frequency)
  - Radial-sampling matrix (objective price and pulse frequency co-vary across 9 or 14 values)
  A complete survey was composed of a complete set of the trials defined by all the rows of the 3 sampling matrices. In total, for all subjects, there were 126 trials per survey: 42 test trials and 84 bracketing trials. For rats F3 and F9 in which there were 9 rows per sampling matrix, the 5 center rows of each sampling matrix were visited twice in a survey. For rats F12, F16, F17, and F18, in which there were 14 rows per sampling matrix, each row was visited only once in a survey.
  For rats F3 and F9, equal logarithmic intervals separated all values along each pseudo-sweep. For rats F12, F16, F17 and F18, equal logarithmic intervals separated the central 10 elements, and the logarithmic intervals separating the highest and second highest values, as well as the lowest and second lowest value, were twice that of the intervals separating the central points. The logarithmic intervals separating the second and third highest value, as well as the second lowest and third lowest value was one and a half times the interval separating the central points. The intervals were spaced in this manner to ensure that the steep portion of the psychometric curves, which determines the values of the location parameters, was sampled more densely than the less critical, flatter regions at the ends (Fig 4D).
  After 5 surveys in this condition, the reward-mountain model was fit to the data using the objective-price function (Eq 7). If the radial-sampling matrix did not pass through, or close to, the crosshair defined by the two model parameters [*Log*_10_(*P*_*obj*_*e*_), *Log*_10_(*F*_*hm*_)], then the radial-sampling matrix was adjusted using the mountain simulator. After an additional 5 surveys, the data were analyzed again, and if the radial-sampling matrix passed through the location parameters, 5 more surveys were conducted. The results obtained during this phase were used to choose experimental-parameter values used in the next phase.

#### The subjective price experiment proper (estimation of the subjective price function)

During this final phase of the study, data were collected so as to fit the full 3D reward model and to derive the subjective-price function. The triadic trial structure (Fig 3) was employed. Nine sampling matrices were employed. An example of a 9-matrix set is presented in Fig 4D. The sampling matrices employed were:

- Pulse-frequency sampling matrix at 0.125 s (pulse frequency varies across 9 or 14 values, fixed 0.125 s price)
- Pulse-frequency sampling matrix at 0.25 s (pulse frequency varies across 9 or 14 values, fixed 0.25 s price)
- Pulse-frequency sampling matrix at 0.5 s (pulse frequency varies across 9 or 14 values, fixed 0.5 s price)
- Pulse-frequency sampling matrix at 1 s (pulse frequency varies across 9 or 14 values, fixed 1 s price)
- Pulse-frequency sampling matrix at 2 s (pulse frequency varies across 9 or 14 values, fixed 2 s price)
- Pulse-frequency sampling matrix at 4 s (pulse frequency varies across 9 or 14 values, fixed 4 s price)
- Pulse-frequency sampling matrix at 8 s (pulse frequency varies across 9 or 14 values, fixed 8 s price)
- Price-sampling matrix (objective price varies across 9 or 14 values, fixed high pulse frequency)
- Radial-sampling matrix (objective price and pulse frequency co-vary across 9 or 14 values)

The radial-sampling matrix, price-sampling matrix, and pulse-frequency sampling matrix (with the price set to 4 s) were similar or identical to those used in the preceding conditions. One survey consisted of a complete set of the trials defined by all rows in all 9 sampling matrices; 2 daily sessions, each about 6 to 7 hours in duration, were required to complete a survey. In total, for each subject, there were 378 trials per survey: 126 test trials and 252 bracketing trials. For rats F3 and F9 in which there were 9 rows per sampling matrix, the 5 center rows of each sampling matrix were visited twice in a survey. For rats F12, F16, F17, and F18, in which there were 14 rows per sampling matrix, each row was visited only once in a survey. After 5 surveys had been collected, the data were analyzed, and, if necessary, the experimental parameters were adjusted as described in the 3D sampling section above. A total of 8-11 surveys were collected following the final adjustment of the experimental parameters.

### Statistical analysis

#### Raw data

The raw data were the onset time and durations of “holds” (intervals during which the lever was depressed by the rat); “release times” (intervals during which the lever was extended but not depressed by the rat) were computed by determining the duration between the end of a given hold and the onset of the subsequent hold. Total work time included 1) the cumulative duration of hold times during a trial, and 2) release times less than 1 s. The latter correction was used because during very brief release intervals, the rat typically stands with its paw over or resting on the lever [23]. Therefore, we treat these brief pauses as work and subtract them from the total release time.

In the calculation of total work and release times, the values prior to the first encounter with the reward were excluded. These data were removed because at the start of a randomized trial, the rat does not yet know the strength or cost of the reward; it needs at least one encounter with the reward to learn the values of the reward parameters [18].

The dependent measure plotted on the 3D graphs is time allocation (*T*): total corrected work time as a proportion of total trial time.

#### The reward-mountain model

The surface that was fitted to the time-allocation (*T*) data obtained at each of the values in the 9 sampling matrices (Fig 4C) as defined by Eq 14.

In the same rats, we have previously estimated the function that translates the experimenter-set pulse frequencies, *F*, to the induced firing frequency [21]. That work shows that the pulse frequencies employed here, which are ≤316 Hz, are within the range of perfect frequency following. Thus, the experimenter-set pulse frequencies were used as proxies for the induced firing frequencies.

Four versions of Eq 14 were fitted to the time-allocation data; in each version, one of the subjective-price functions described by Eqs 7, 9, 12 and 13 was substituted for *P*_*sub*_. An additional version of Eq 14 was fitted to the data from rat F17. This is a variant of the sigmoidal-slope function (Eq 9) with the $P_{sub_{bend}}$ parameter set to 0.5, a value close to the across-subject mean of the estimates obtained when this parameter was free to vary.

#### Resampling

The dataset for each rat was assessed individually. To fit the model to the data, a bootstrapping method [42] was employed, as follows.

A survey refers to one complete test of the 9 sampling matrices. The complete dataset consists of *n* (7-11) surveys per rat. Thus, *n* *T* values were obtained for each combination of pulse-frequency and objective-price values (i.e., for each row of a sampling matrix). (In Fig 4, each combination of pulse-frequency and objective-price values is represented by a colored marker.) A resampled dataset was produced by drawing *n* *T* values, with replacement, from the set obtained at each combination of pulse-frequency and objective-price values. Rats F12, F16, F17, F18, were tested at 126 combinations of pulse-frequency and objective-price values (14 combinations × 9 sampling matrices). Resampled *T* values for a particular combination of pulse-frequency and objective-price values might consist of data from surveys {2,3,5,1,8,8,2,10,1,1}, the second combination from surveys {4,9,1,9,4,7,5,3,6,8}, etc. One thousand such resampled subsets of *T* values were generated for each of the 126 combinations of pulse-frequency and objective-price values. The 3D mountain model (Eq 14) was then fit individually to each of the 1000 datasets using the non-linear least-squares routine in the MATLAB Optimization Toolbox (The Mathworks, Natick, MA). The mean values of the parameters {*a*, *g*, *F*_*hm*_, *P*_*sub*_*e*_, *T*_*max*_, *T*_*min*_} across the 1000 data sets were determined, along with the parameters of each of the subjective-price models {$P_{sub_{min}}$, $P_{sub_{bend}}$, *K*_*h*_, *K*_*x*_}. 95% confidence intervals about each estimated parameter were calculated by excluding the lowest 2.5% and highest 2.5% of the estimates.

### Histology

Following the estimation of the subjective-price function, the subjects underwent two additional experiments [43], which lasted 8 months. After completion of these experiments, the rats were anesthetized with a lethal dose of sodium pentobarbital. The brains were removed and were fixed with a 10% formalin solution for at least two weeks. Coronal sections, 30 to 40 *μ*m thick, were cut with a cryostat, and tip locations were determined under low magnification with reference to the stereotaxic atlas of Paxinos and Watson [41].

## Results

The fits of the reward-mountain models incorporating the objective-price function, linear-price function, and the sigmoidal-slope function converged in all cases, thus providing parameter estimates and a statistical basis for evaluating goodness of fit. In contrast, the reward-mountain model incorporating the exponential-price function converged successfully only in the cases of rats F3, F16, and F17. In the remaining cases, the fits failed to converge, and goodness of fit cannot be evaluated.

### Surface fits and contour graphs

**Fig 5:**
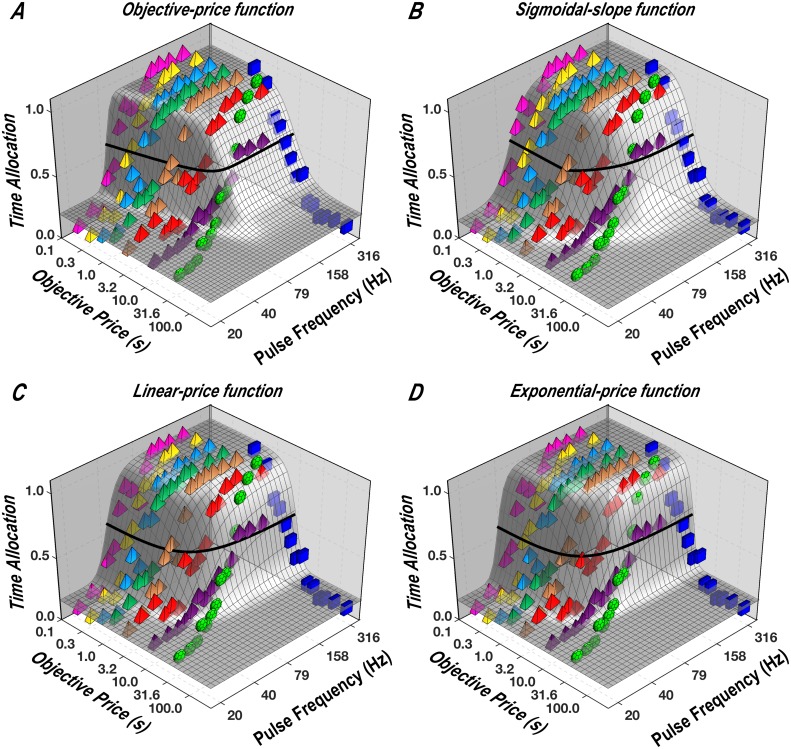
Time allocation as a function of the strength and cost of reward. The colored symbols represent the proportion of trial time allocated to reward seeking by rat F16 as a function of price and pulse frequency. The corresponding legend and contour plots are presented in Fig 6. Each of the fitted surfaces is defined by one of the four subjective-price functions (Eqs 7, 9, 12 and 13). Analogous plots for the remaining rats are shown in Figs A, B, C, E and F in S1 File.

The surfaces generated by the reward-mountain models (Eq 14) and their proximity to the data are illustrated in Fig 5. This figure shows the empirical data from rat F16 superimposed on the fitted surfaces defined by each of the four subjective price functions (Eqs 7, 9, 12 and 13) Analogous plots for the remaining rats are shown in Figs A, B, C, E and F in S1 File.

The black contour line slices the mountain at the “mid-range” time allocation (half-way between *T*_*min*_ and *T*_*max*_). In functional form, the contour line is expressed as:

$$\begin{array}{c} Log\left(F_{mid}\right)=Log\left(F_{hm}\right)+[\frac{1}{g}\times Log(\frac{P_{sub}\left(P_{obj}\right)}{P_{sub\_e}\left(P_{obj\_e}\right)-P_{sub}\left(P_{obj}\right)})] \end{array}$$ (15)

where

*F_mid_* = the pulse frequency at which time allocation is mid-way between its minimum and maximum values, and

*P_sub_*(*P_obj_*) = the output of one of the four proposed subjective price functions.

The derivation of Eq 15 is presented in S1 File.

**Fig 6:**
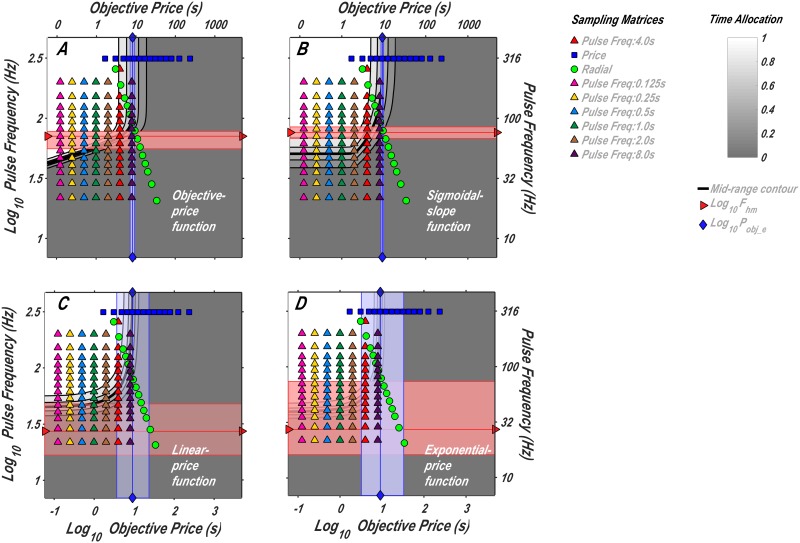
Contour plots corresponding to the surfaces in Fig 5. Time allocation is represented by the grey level, as shown in the bar at the upper right. Each colored symbol represents a tested pair of objective-price and pulse-frequency values (i.e., a row of a sampling matrix); each color-shape combination denotes a different pseudo-sweep. The horizontally oriented series of blue squares represents the price pseudo-sweep, whereas the diagonally oriented series of green circles represents the radial pseudo-sweep. All the remaining series are pulse-frequency pseudo-sweeps carried out at different prices. The vertical blue line represents the fitted value of the *P*_*obj*_*e*_ location parameter, whereas the horizontal red line represents the fitted value of the *F*_*hm*_ location parameter (Eq 14). The colored bands surrounding the location-parameter lines are 95% confidence intervals. Analogous plots for the remaining rats are shown in Figs G, H, I, K and L in S1 File.

The contour graphs corresponding to the fitted surfaces in Fig 5 are shown in Fig 6. Superimposed on the contour graphs are colored symbols denoting the pseudo-sweeps; each symbol represents a tested pair of objective-price and pulse-frequency values. Also shown in Fig 6 are the fitted location parameters {*P*_*obj*_*e*_,*F*_*hm*_} of the reward-mountain model [18] and their surrounding 95% confidence intervals. (Please see Eq 14 for the definitions of the location parameters.) Note that the confidence intervals are narrowest in the case of the sigmoidal-slope function. Analogous plots for the remaining rats are shown in Figs G, H, I, K and L.

### Derivation of the trade-off between pulse frequency and objective price

It is not always easy to visually discern the goodness of fit of a curved surface to multiple data points (e.g., in Fig 5). To complement the 3D depiction, 2D pulse-frequency-vs.-objective price trade-off functions were plotted along with interpolated data points: the pulse frequencies and/or objective prices corresponding to the mid-range *T*.

Spline functions (a series of smoothly joined polynomial segments) were fit to each of the 2D psychometric plots of time allocation vs. pulse frequency and time allocation vs. objective price using the MATLAB spline-function routine. For example, when 10 surveys were collected, there were 90 2D plots (9 sampling matrices × 10 surveys). For each spline function fit to the data from a test carried out with the pulse-frequency sampling matrix, the pulse frequency corresponding to the mid-range time-allocation value (*F*_*mid*_) was determined. Similarly, for each spline function fit to the data from a test carried out with the price-sampling matrix, the objective price corresponding to the mid-range time-allocation value (*P*_*obj*_*mid*_) was determined. For each spline function fit to the data from the test carried out with the radial-sampling matrix, both *F*_*mid*_ and *P*_*obj*_*mid*_ were determined. Given that these values were interpolated from the empirical data, they are independent of the mountain model, and thus their proximity to the fitted surface reflects how well this surface fits the data.

**Fig 7:**
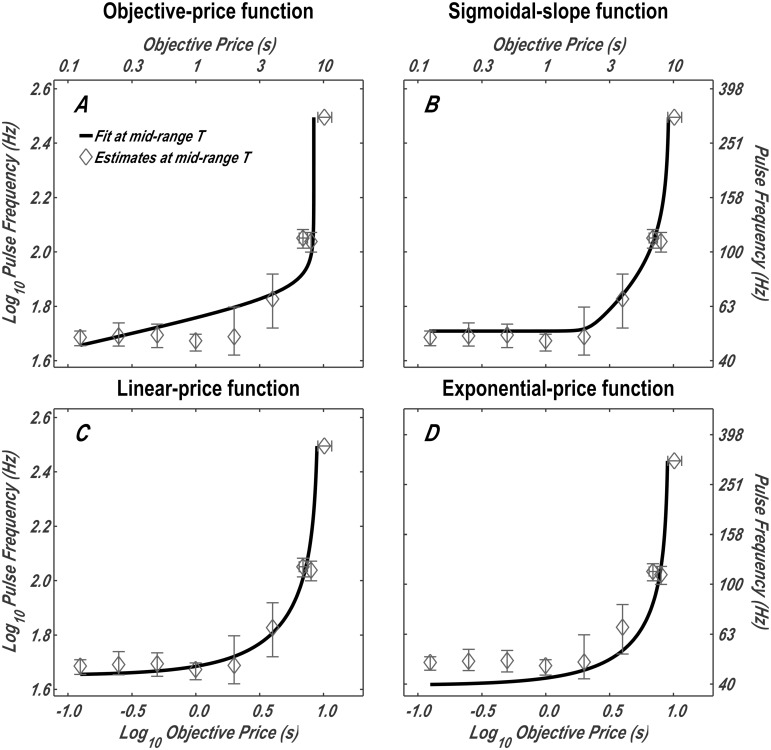
Comparison between interpolated data points and pulse-frequency-versus-objective-price trade-off functions derived from the surface fits. The solid line is the contour in Fig 6 representing mid-range time allocation (half-way between *T*_*min*_ and *T*_*max*_) by rat F16. The corresponding data points were interpolated by means of spline fits to the data from the pulse-frequency, price, and radial pseudo-sweeps. Analogous plots for the remaining rats are shown in Figs M, N, O, Q and R in S1 File.

The bootstrap method [42] was employed to estimate the mean *F*_*mid*_ and *P*_*obj*_*mid*_ values and the surrounding confidence intervals. One thousand resampled *F*_*mid*_ values were obtained from the tests carried out with each of the 7 pulse-frequency sampling matrices and the single radial-sampling matrix. One thousand resampled *P*_*obj*_*mid*_ values were obtained from the tests carried out with each price-sampling matrix and radial-sampling matrix. The means of the 1,000 resampled values were calculated, and the corresponding 95% confidence interval was obtained by excluding the lowest 2.5% and highest 2.5% of the estimates. These means and confidence intervals are plotted in Fig 7 along with the pulse-frequency-vs.-objective-price trade-off functions derived from the surface fits. Analogous plots for the remaining rats are shown in Figs M, N, O, Q and R in S1 File.

It is important to keep in mind that Fig 7 shows deviations in a plane orthogonal to the one in which goodness-of-fit was assessed. The deviations of the interpolated means from the curves in Fig 7 are horizontally oriented with respect to the 3D structure (Fig 5); they are in the plane defined by the pulse frequency and price. In contrast, the deviations on which the goodness-of-fit measure is computed are vertically oriented; they are arrayed along the time-allocation dimension (the vertical axis of Fig 5). Where the fitted surface is steep, a small horizontal deviation corresponds to a large vertical one. Whereas the deviations shown in Fig 7 are restricted to a single altitude, the level half-way between the minimum and maximum time allocation, the the goodness-of-fit measure is based on the vertical deviation of all data points from the fitted surface (Fig 5). What Fig 7 emphasizes are systematic deviations of neighboring pseudo-sweep data from the fitted surface (e.g., in the case of the three shortest durations in Panel **D**). The same point is made rather subtly in Fig 5 by the fact that the data points between the middle and shoulder of the corresponding curves in Panel **D** lie beneath the fitted surface.

### Subjective price as a function of objective price

In addition to the six parameters of the reward-mountain model (*a*, *g*, *Log*_10_(*F*_*hm*_), *Log*_10_(*P*_*sub*_*e*_), *T*_*max*_, *T*_*min*_) [18], the surface fits return the subjective-price parameters ($P_{sub_{bend}}$, $P_{sub_{min}}$, *K*_*h*_, *K*_*x*_), which are listed in Table 2 for rat F16 and in Tables B-G in S1 File for all six rats.

**Table 2:** Best fitting parameter values for rat F16.

Function	Parameter	Fitted estimate	CB low	CB high	CB width
Objective	*a*	1.99	1.52	2.91	1.39
	*g*	9.42	7.43	24.08	16.65
	*Log*_10_(*F*_*hm*_)	1.85	1.75	1.89	0.14
	*Log*_10_(*P*_*sub*_*e*_)	0.92	0.86	0.98	0.11
	*T*_*max*_	0.94	0.90	0.96	0.05
	*T*_*min*_	0.20	0.18	0.23	0.05
Sigmoidal	*a*	2.35	2.06	2.72	0.66
	*g*	3.06	2.66	3.53	0.87
	*Log*_10_(*F*_*hm*_)	1.88	1.83	1.93	0.10
	*Log*_10_(*P*_*sub*_*e*_)	0.97	0.93	1.00	0.07
	$Log_{10}\left(P_{sub_{min}}\right)$	0.33	0.22	0.42	0.20
	$P_{sub_{bend}}$	0.21	0.03	0.52	0.49
	*T*_*max*_	1.00	1.00	1.00	0.00
	*T*_*min*_	0.15	0.13	0.17	0.04
Linear	*a*	15.44	5.79	25.00	19.21
	*g*	2.01	1.65	2.49	0.84
	*K*_*h*_	0.04	0.02	0.10	0.08
	*Log*_10_(*F*_*hm*_)	1.44	1.22	1.68	0.46
	*Log*_10_(*P*_*sub*_*e*_)	0.14	0.06	0.29	0.23
	*T*_*max*_	1.00	1.00	1.00	0.00
	*T*_*min*_	0.16	0.15	0.18	0.03
Exponential	*a*	16.80	2.65	25.00	22.35
	*g*	2.06	1.62	2.97	1.35
	*K*_*x*_	0.04	0.01	0.16	0.14
	*Log*_10_(*F*_*hm*_)	1.44	1.21	1.87	0.66
	*Log*_10_(*P*_*sub*_*e*_)	0.17	0.06	0.61	0.55
	*T*_*max*_	1.00	1.00	1.00	0.00
	*T*_*min*_	0.16	0.15	0.18	0.04

CB = 95% confidence band.

**Fig 8:**
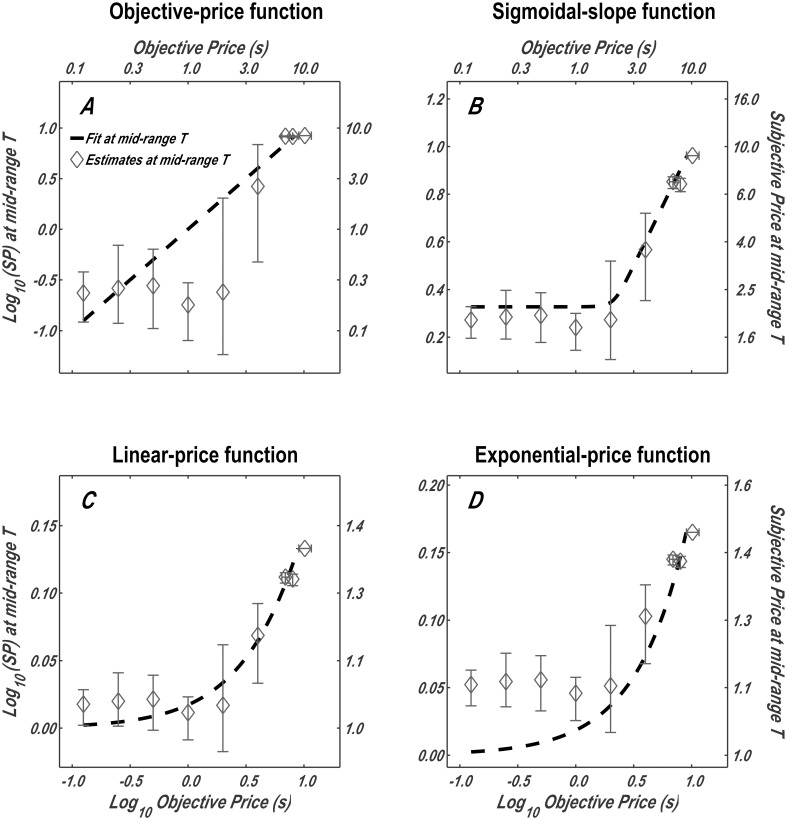
The subjective-price functions obtained by fitting the four models. The dashed lines are the subjective-price functions corresponding to the contours in Fig 7 representing mid-range time allocation (half-way between *T*_*min*_ and *T*_*max*_) by rat F16. These functions were computed by back-solving Eq 15 for *P*_*sub*_, given the fitted values of *F*_*hm*_, *g*, and *P*_*sub*_*e*_ (e.g., Table 2) and the values of the two independent variables at each point along the contour line in Fig 7. The data points were transformed in the same manner. Analogous plots for the remaining rats are shown in Figs S, T, U, W and X in S1 File.

These parameter values were substituted into the subjective price functions (Eqs 7, 9, 12 and 13), which are plotted in Fig 8 (dashed lines). The interpolated data points in Fig 7 ($Log_{10}\left[P_{obj_{mid}}\right]$, *Log*_10_[*F*_*mid*_]) were transformed into subjective-price values, using the parameters obtained from the surface fits. The following equation (derived in S1 File) was used to carry out this transformation:

$$\begin{array}{c} Log_{10}\left(P_{sub_{mid}}\right)=Log_{10}\left(R_{rel}\right)+Log_{10}\left(P_{sub\_e}\right) \end{array}$$ (16)

where

$R_{rel}\;=\;\frac{F^{g}}{F^{g}\;+\;F_{hm}^{g}}$

$P_{sub_{mid}}$ = the subjective price corresponding to mid-range time allocation (half-way between *T_min_* and *T_max_*)

This transformation provides a visual depiction of the four fitted subjective-price models along with interpolated data points. It is evident from Fig 8**A** and panels A of Figs S-X in S1 File that the deviations of the interpolated data points from the fitted surfaces are particularly large and systematic in the case of the objective-price function. The form of this function is very different from the trajectory of the data points. It would not have helped had we allowed the y-intercept or exponent to assume values other than one because the resulting functions would still be linear in double logarithmic coordinates, whereas the interpolated data points clearly trace out non-linear trajectories. Analogous plots for the remaining rats are shown in Figs S, T, U, W and X in S1 File.

### Model comparisons

The AIC [40] was employed to determine which model best fits the data. The AIC statistic provides an estimate of the relative superiority of the tested models by balancing the goodness of fit with the complexity of the model (the number of parameters). The AIC statistic was calculated separately for the fit of each model to the (non-resampled) dataset for each rat and is presented in Table 3 along with associated statistics. On a relative scale, the more negative the AIC value, the better the model. The difference between the AIC for all of the models and the highest ranked model, termed ΔAIC, was determined. The likelihood corresponding to each ΔAIC was then calculated (*likelihood* = *e*(-Δ*AIC/2*)) to estimate the probability that a given model is better than the highest ranked model. The Akaike weight is the probability that the model is the best model among the whole set of candidate models (*Akaike weight* = *likelihood* / *sum of likelihood of all models*). The evidence ratio is the proportion of instances in which the highest ranked model is more likely to be better than a given model (*evidence ratio* = *Akaike weight of highest ranked model* / *Akaike weight of given model*). The goodness-of-fit statistics for each of the four models are presented from best to worst, for each rat, in Table 3. The three cases in which the reward-mountain model incorporating the exponential-price function (rats F9, F12 and F18) did not converge on best-fitting values of the exponent are designated “DNC.”

**Table 3:** Model comparison.

Rat	Model	AIC Value	ΔAIC	Likelihood	Akaike Weight	Evidence Ratio
F3	Sigmoidal	-2018.1	0.0	1.00E+00	1	1.00E+00
	Exponential	-1968.4	49.7	1.64E-11	0	6.11E+10
	Linear	-1960.6	57.5	3.34E-13	0	2.99E+12
	Objective	-1453.6	564.4	2.75E-123	0	3.63E+122
F9	Sigmoidal	-1876.8	0	1.00E+00	1	1.00E+00
	Linear	-1814.7	62.1	3.32E-14	0	3.01E+13
	Objective	-1333.4	543.4	1.02E-118	0	9.84E+117
	Exponential	DNC	DNC	DNC	DNC	DNC
F12	Sigmoidal	-1841.8	0	1.00E+00	1	1.00E+00
	Linear	-1790.8	51	8.23E-12	0	1.21E+11
	Objective	-1447.4	394.4	2.24E-86	0	4.47E+85
	Exponential	DNC	DNC	DNC	DNC	DNC
F16	Sigmoidal	-1415.2	0	1.00E+00	1	1.00E+00
	Linear	-1376.8	38.4	4.57E-09	0	2.19E+08
	Exponential	-1375.7	39.5	2.65E-09	0	3.78E+08
	Objective	-1166.5	248.7	9.88E-55	0	1.01E+54
F17	Exponential	-1580.6	0	1.00E+00	0.63	1.00E+00
	Sigmoidal	-1579.5	1.1	5.91E-01	0.37	1.69E+00
	Linear	-1566.2	14.4	7.44E-04	0	1.34E+03
	Objective	-1385.1	195.5	3.48E-43	0	2.87E+42
F17 FB	Sigmoidal FB	-1581.5	0	1.00E+00	0.5	1.00E+00
	Exponential	-1580.6	0.9	6.26E-01	0.31	1.60E+00
	Sigmoidal	-1579.5	2	3.70E-01	0.19	2.70E+00
	Linear	-1566.2	15.3	4.65E-04	0	2.15E+03
	Objective	-1385.1	196.5	2.18E-43	0	4.59E+42
F18	Sigmoidal	-2200	0	1.00E+00	1	1.00E+00
	Linear	-2164.3	35.7	1.80E-08	0	5.56E+07
	Objective	-1582.5	617.5	8.13E-135	0	1.23E+134
	Exponential	DNC	DNC	DNC	DNC	DNC

FB refers to the version of the sigmoidal-slope function in which the value of the $P_{sub_{bend}}$ parameter is fixed at 0.5. DNC designates fits that failed to converge.

The Akaike weights show that the sigmoidal-slope function provides the best fit by far to five of the six datasets. In those cases, the Akaike weight for the sigmoidal-slope function equals one (rounded for 5 decimal places), whereas it rounds to zero for the remaining functions. In these five datasets, the evidence ratios favor the sigmoidal-slope function over the next best-fitting function by seven to thirteen orders of magnitude.

In the case of rat F17, the exponential-price function fit marginally better than the sigmoidal-slope function: the Akaike weights are 0.63 and 0.37 for the exponential-price function and the sigmoidal-slope function, respectively. This discrepancy would have been larger had we evaluated the models by means of the Bayes Information Criterion (BIC) [44] rather than the AIC because the BIC penalizes extra parameters more heavily than the AIC. However, by fixing the value of $P_{sub_{bend}}$ (to the mean of the fitted values for all six rats), the sigmoidal-slope function is left with only one free parameter, placing it on the same footing as the exponential subjective-price functions. This “fixed-bend” variant of the model fits the data for rat F17 marginally better than the exponential subjective-price function: the Akaike weights are 0.50 and 0.31 for the fixed-bend variant of the sigmoidal-slope function and the exponential-price function, respectively.

In summary, the sigmoidal-slope function provides the best fit to the data in five of six cases, and the remaining case can be regarded as a draw.

### Histology

**Fig 9:**
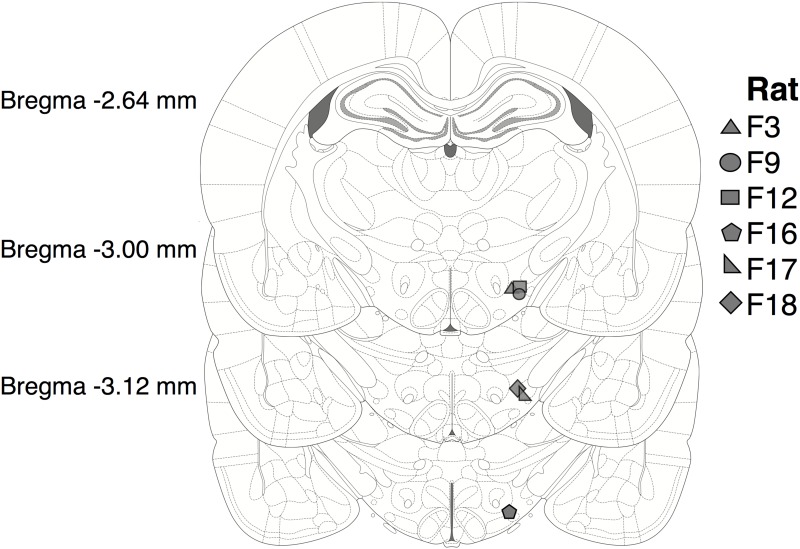
Location of the electrode tips. The filled symbols represent the location of the electrode tips, as determined by examination of the stained brain sections. The plates are from the Paxinos and Watson atlas [41].

Fig 9 shows the electrode-tip locations, which are all situated in the lateral hypothalamus. There is no apparent relationship between the subjective-price results and the location of the electrode tip.

## Discussion

In their landmark paper on motivation and behavioral decision making [45], McFarland and Sibley assert that “it is always possible to classify the behavioral repertoire of a species in such a way that the classes are mutually exclusive in the sense that the members of different classes cannot occur simultaneously.” A simplified rephrasing reads: “animals cannot ‘do more than one thing at a time’.” The concept of the opportunity cost of time flows naturally from this postulate. Time invested in achieving one objective is necessarily subtracted from the time available to achieve alternate goals that cannot be pursued simultaneously. The foregone benefits that would have accrued from achievement of those alternate goals thus contribute to the cost of the objective that was ultimately sought.

### Competing accounts of the subjective-price function

In this paper, we ask how the subjective value of time spent working for rewarding electrical brain stimulation is related to the opportunity cost incurred. Four versions of the opportunity-cost function were tested. The objective-price function is the simplest: time is translated into subjective opportunity cost on the basis of strict proportionality. The sigmoidal-slope function converges on the objective-price function when the time required to procure a reward is long but deviates from it as the required time becomes very short. This deviation is driven by the decreasing availability of alternate, substitutable activities. Once the time required to procure a reward becomes so brief as to preclude performance of a beneficial alternate activity, then further reduction in the required time ceases to reduce the subjective opportunity cost. The remaining two functions treat the valuation of opportunity costs as an instance of delay discounting. The longer the time required to procure the reward, the longer the delay between initiation of the reward-seeking action and its fulfillment. These subjective-price functions discount the reward progressively as its opportunity cost grows. The linear- and exponential-price variants differ only in the form of the discounting function applied.

As we argue below, determining which of the four functions fits best is important, not only for accurate modeling of performance for rewarding brain stimulation, but also for the more general issue of how opportunity costs contribute to decisions about selection and achievement of physiological, reproductive and social goals.

### The subjective-price function and the reward-mountain model

The reward-mountain model (Eq 14) expresses time allocated to the pursuit of rewards as a function of their strength and cost. Work time is an unavoidable component of the costs entailed in operant pursuit of reward. Thus, the form of the reward mountain must reflect the way subjective opportunity costs grow as the work time required to obtain a reward increases. In effect, the subjective-price function is embedded within the reward-mountain model, either implicitly, as in the initial versions [14, 15], or explicitly, as in a recent revision [18]. The goodness of fit of the reward-mountain model thus provides a criterion for selecting among different variants of the subjective-price function. That is the approach adopted here.

**Fig 10:**
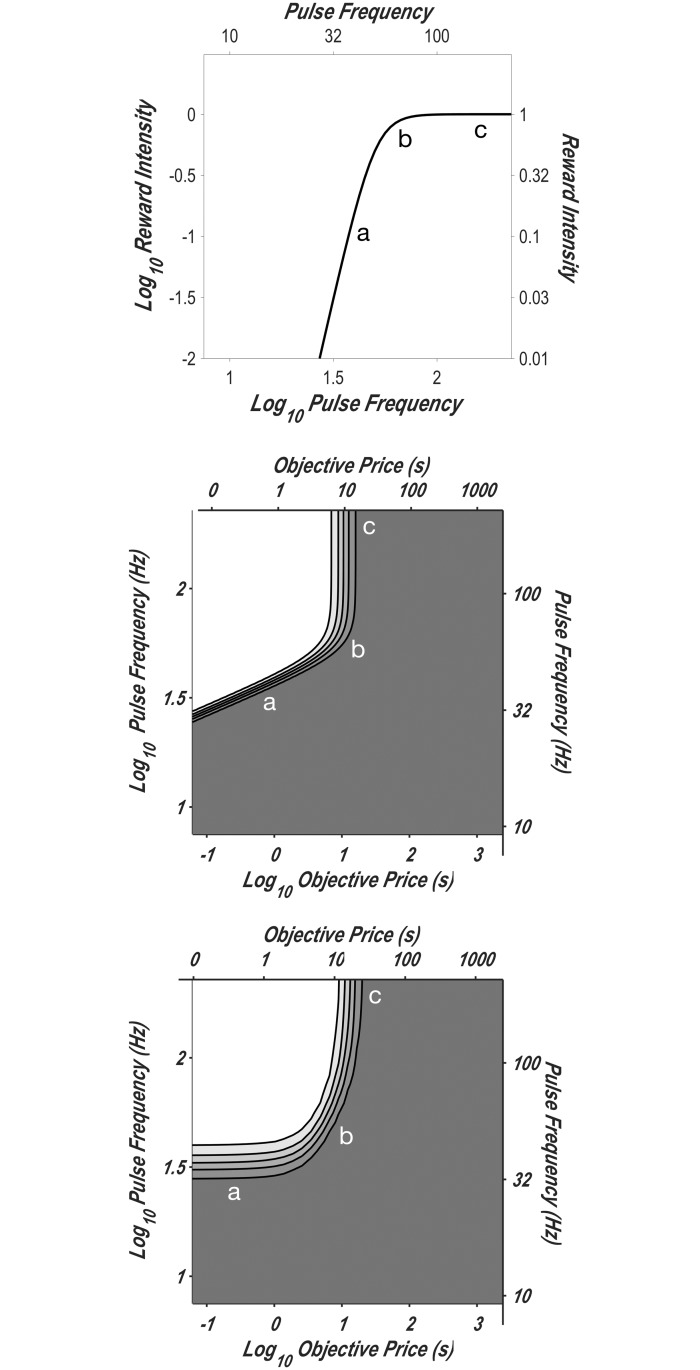
The contours of the reward mountain reflect the form of the reward-intensity-growth function. Upper panel: the reward-intensity-growth function, as described by Simmons and Gallistel [13] and by Sonnenschein, Conover and Shizgal [46]; the parameters {*F*_*hm*_,*g*} are from the fit of the objective-price function to the data from rat F18. Middle panel: Contour map of the reward-mountain variant that incorporates the objective-price function; the parameters {*a*,*F*_*hm*_,*g*} are again from the fit of the objective-price function to the data from rat F18. The contours are rotated traces of the reward-intensity-growth function in the upper panel. Lower panel: Contour map of the reward-mountain variant that incorporates the sigmoidal-slope function; the parameters {*a*,*F*_*hm*_,*g*,$P_{sub_{bend}}$,$P_{sub_{min}}$} are from the fit of the sigmoidal-slope function to the data from rat F18. These contours reflect the non-linear form of both the reward-intensity-growth and subjective-price functions. The contours defined by the reward-mountain variants incorporating the objective- and linear-price functions (not shown) also bend toward the horizontal at low prices.

According to the reward-mountain model, the experimenter can compensate for an increase in the price of rewarding electrical brain stimulation by making a compensatory increase in the strength of the stimulation; the rat is thereby induced to hold constant its allocation of time to reward pursuit in the face of the price increase. This trade-off between the cost and strength of the reward is reflected in the shape of the contour lines that describe the reward-mountain surface (Eq 15; Figs 2 and 5). Underlying the trade-off between the two objective variables, stimulation strength and required work time, is a reciprocal relationship between their subjective counterparts, subjective reward intensity and subjective price [14, 15, 18]. Time allocation remains fixed provided that the ratio of subjective reward intensity and subjective price doesn’t change. In the early versions of the model [14, 15], the objective-price function was assumed, and thus the contour lines trace out what is called the “reward-intensity-growth function” (Fig 10, upper panel), which maps the relationship between subjective reward intensity and stimulation strength. This function determines the magnitude of the changes in stimulation strength required to compensate for changes in subjective price.

Simmons and Gallistel [13] demonstrated that the reward-intensity-growth function rises as a power function over low stimulation strengths, decelerates at higher stimulation strengths, and eventually levels off. Sonnenschein and Shizgal [46] used a logistic function to describe this behavior. In double logarithmic coordinates, the logistic reward-intensity-growth function is shaped like an upside-down hockey stick, with a straight, diagonally oriented handle (Fig 10, upper panel: **a**) and a horizontally oriented blade (Fig 10, upper panel: **c**). Stimulation strength is represented by the y-axis in the coordinate space for the contour map of the mountain (Fig 10, middle and lower panels), whereas it is represented by the x-axis in plots of the reward-intensity-growth function (Fig 10, upper panel). Thus, in the variant of the reward-mountain model that incorporates the objective-price function [14, 15] (Fig 6**A**), the contour lines trace out a rotated version of the reward-intensity-growth function (Fig 10, middle panel). When prices are low, relatively weak stimulation strengths suffice to drive time allocation above its minimal value. The reward-intensity-growth function rises steeply over this range, and thus a small increment in stimulation strength suffices to offset a given change in price, causing the contour lines to rise with a shallow slope (Fig 10, middle panel: **a**; note the different scales of the x- and y-axes). As the reward-intensity-growth function decelerates, ever-larger offsetting increments in stimulation strength are required to offset a given increase in price, and the contour lines bend upwards (Fig 10, middle panel: **b**). Once the reward-intensity-growth function levels off, further changes in stimulation strength can no longer compensate for increments in price, and the contour lines run vertically (Fig 10, middle panel: **c**).

As Eq 15 implies, the shape of the contour lines generated by the versions of the reward-mountain model that incorporate the three more complex subjective-price functions under consideration (sigmoidal, linear, and exponential) is no longer determined exclusively by the reward-intensity-growth function but also reflects the form of the subjective-price function (e.g., Fig 10, lower panel). As the price is reduced to very low values, all three of these subjective-price functions bend the contour lines toward the horizontal (Figs 6**B**, 6**C** and 6**D** and 10:**lower panel**), albeit in different ways. The fact that reward-mountain surfaces of different shapes are produced by the four subjective-price functions provides is the basis for the method used here to determine which function fits the data best.

### The sigmoidal-slope function fits best

In five of the six datasets, the Akaike weights for the sigmoidal-slope function are overwhelmingly greater than those for the remaining subjective-price functions. The exponential function noses out the sigmoidal-slope function in the case of the remaining dataset (from rat F17), but when the number of free parameters in these two subjective-price functions is equated by fixing the value of the $P_{sub_{bend}}$ parameter at 0.5 (the mean estimate for the six datasets), the sigmoidal-slope function achieves a marginally better Akaike weight than the exponential-price function. Although from a model-fitting perspective, there is little basis for choosing between these two functions in the case of the data from rat F17, the sigmoidal-slope function provides by far the best fit to the data from the entire group of rats.

### Compatibility of the different subjective-price functions with normative behavior

According to the expression for payoff embedded within the reward-mountain model (Eq 1), increases in the subjective intensity of a reward compensate perfectly for increases in its subjective price. In the case of the objective-price function, this is true for objective prices, as well as for subjective ones, because the two are equal. The ratio of reward intensity to objective price is simply a rate, analogous to the key determinant of choice in influential theories of operant performance [28, 47, 48]. An agent employing the objective-price function to evaluate opportunity costs would be indifferent when offered a choice between an intense reward requiring a long work time (high price) and a weaker reward requiring a shorter work time (lower price), provided that the ratio of reward intensity and work time were the same for either option (i.e., reward intensity accrued at the same rate). Such behavior can be said to be “price-neutral.” The objective price *per se* has no bearing on choice; it influences choice only in terms of its relationship to the reward intensity: It is the ratio of the reward intensity to the objective price that matters. In contrast, an agent that eschews high-priced rewards in favour of lower-priced rewards earned at the same rate can be said to be “price-averse,” and an agent that seeks out such high-price rewards while neglecting lower-priced rewards earned at the same rate can be said to be “price-seeking.”

Let us assume that the value of a given volume of a 1-molar solution of sucrose is ten times the value of the same volume of a 0.1-molar solution. Faced with a choice between 1 ml of the 1-molar solution that can be earned with 50 s of work and 1 ml of the 0.1-molar solution that can be earned with 5 s of work, a price-averse agent would choose the latter, a price-seeking agent would choose the former, and a price-neutral agent would be indifferent. To achieve equipreference by the price-averse agent, the price of the 1-molar solution would have to be reduced. At the equipreference point, the price-averse agent would be leaving “money on the table” because it could harvest sucrose at higher rate by choosing the 1-molar solution exclusively. The price-seeking agent would have the complementary problem. To achieve equipreference by that agent, the price of the 1-molar solution would have to increased. At the equipreference point, that agent could have earned sucrose at a higher rate by choosing the 0.1 molar solution exclusively. Only the price-neutral agent would choose normatively, manifesting equipreference only when the two options deliver sucrose at the same rate.

**Fig 11:**
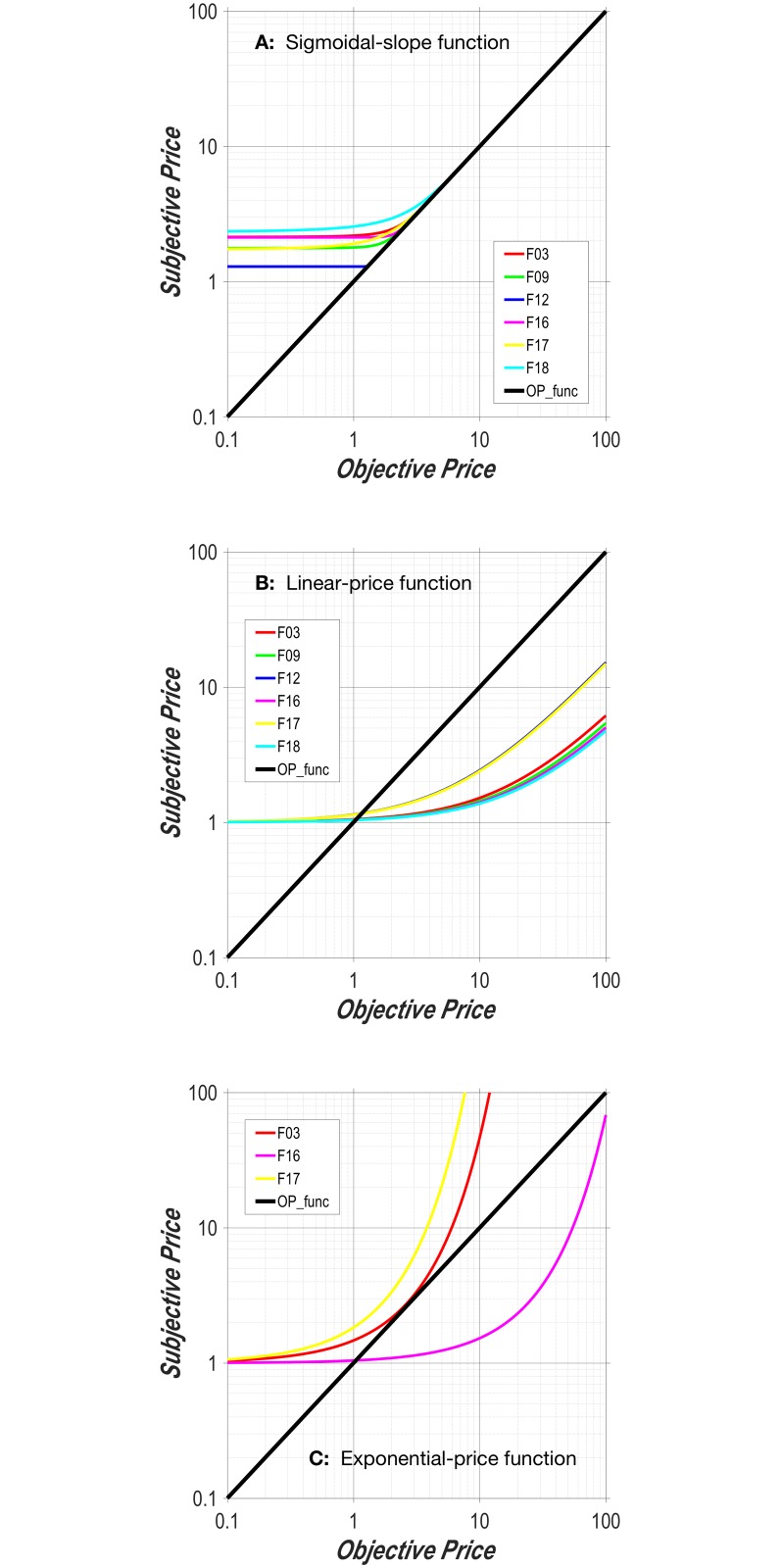
Fitted subjective-price functions for each rat. The black diagonal line in each panel is the objective-price function (“OP_func”). The fits of the sigmoidal-slope, linear-price, and exponential-price models to the data from each rat are shown in color in panels **A**, **B**, and **C**, respectively. Only three curves are shown in panel **C** because the fits of the exponential-price function in question converged only in these cases. Unlike the plots in Fig 1 and in Figs S-X in S1 File, the y-axes in these plots are scaled identically.

The mean value of the fitted $P_{sub_{min}}$ parameter obtained from the fits of the sigmoidal-price function is 1.85 s (the antilog of 0.26, the value given in Table 4 for $Log_{10}\left(P_{sub_{min}}\right)$). Given this value and $P_{sub_{bend}}=0.5$, the subjective price is within 1% of the objective price once the latter exceeds 3.18 s. In other words, the fitted sigmoidal-price functions converges on the objective-price function quite rapidly, as can be seen in the plots of the individual fits in Fig 11**A**. Beyond the point of convergence, an agent employing the sigmoidal-slope function behaves in a price-neutral, normative manner. Agents employing the subjective-price functions derived from temporal discounting do not (with one exception noted below).

**Table 4:** Sigmoidal-slope function: Estimated parameter values.

Parameter	mean	SEM	median	IQR
$Log_{10}\left(P_{sub_{min}}\right)$	0.26	0.04	0.29	0.10
$P_{sub_{bend}}$	0.50	0.18	0.41	0.44

Measures of central tendency and dispersion of the parameter estimates obtained for the sigmoidal-slope function. SEM = standard error of the mean; IQR = inter-quartile range.

The fitted values of the *K*_*h*_ parameter for the six datasets range from 0.04 to 0.14 (Tables B-G in S1 File). These values of the parameter of the linear-price function generate strongly price-seeking behavior because the subjective price rises much more slowly than the objective price. This is illustrated in Fig 11**B** and by the discrepancy between the *P*_*obj*_*e*_ and *P*_*sub*_*e*_ values (Table H in S1 File). Whereas the subjective-price estimates produced by the sigmoidal-slope function have converged on the corresponding objective prices well before *P*_*obj*_*e*_ the subjective-price estimates produced by the two functions derived from temporal discounting deviate substantially from the corresponding objective prices. For example, time allocation by rat F03 was halfway between its minimal and maximal values when the objective price (*P*_*obj*_*e*_) was 14.12 s (red curve in Fig 11**B**). According to the fitted value of the *K*_*h*_ parameter for this rat (0.05), this *P*_*obj*_*e*_ value is equivalent to a subjective price of only 1.73 s. Doubling the objective price to 24.24 s, and thus halving the rate at which reward intensity accrues, increases the subjective price generated by this linear-price function by only a factor of 1.39, to 2.41 s. In comparison to a price-neutral agent, an agent behaving in the manner prescribed by this linear-price function would be at a strong disadvantage in environments that included high-price rewards due to its exaggerated proclivity to pursue high-price options. The only value of the *K*_*h*_ parameter that would generate approximately normative choice would be unity. None of the fitted values come close.

Among the four subjective-price functions evaluated here, the exponential-price function generates the most extreme deviations of subjective from objective prices. For example, consider the best-performing fit of this function, which was obtained in the case of the data from rat F17 (yellow curve in Fig 11**C**). Time allocation is halfway between its minimal and maximal values at an objective price of 8.77 s, and the corresponding subjective price is 198.31 s (off-scale in Fig 11**C**). The already-large discrepancy between the objective and subjective price explodes as the objective price is further increased. Given the fitted value of 0.6 for the *K*_*x*_ parameter, doubling the objective price to 17.54 s drives the subjective price to 37,197.6 s. In that range, choice would be spectacularly price-averse.

Deviation from normative choice cannot in itself disqualify a functional form from consideration. Indeed, studies of decision making in humans [7, 8, 49], as well as in laboratory animals [50, 51], have documented many such systematic deviations. Nonetheless, flamboyant deviations, such as those predicted by the exponential-price function fitted to the data from rat F17, demand particularly strong empirical confirmation. This is lacking in the case of the subjective-price functions derived from temporal discounting. Not only do these functions fare much less well than the sigmoidal-slope function in describing the data from the current study, they also deviate from the results of a previous study in which a different empirical method, conjoint measurement, was used [52, 53]. The results of that study are consistent with the sigmoidal-price function and the normative behavior it generates.

### Implications

The results strongly support the recommendation [18] that the version of the reward-mountain model incorporating the sigmoidal-slope function should be used in future applications. Note that the objective-price function provided the worst fit to all six datasets (Table 3). Unlike the other functions considered, the objective-price function generates contour lines that continue to run diagonally as the price is reduced to very low values (Fig 10, middle panel: **a**). In contrast, the sigmoidal-slope function forces the contour lines to run horizontally at the lowest prices (Fig 10, lower panel: **a**), which improves the fit of the model and provides a more reasonable account of behavior when prices are so low as to preclude substitution of a gainful alternative activity.

It would be impractical to obtain individual estimates of the parameters of the sigmoidal-slope function in all future applications of the reward-mountain model. That would require repeating the current experiment each and every time the reward-mountain model were applied to new data, which would extend the required testing period unreasonably. Instead, we recommend fixing the parameters of the sigmoidal-slope model to the mean values reported here, which are reported in Table 4. Given these values, the subjective price converges rapidly with the objective price; once the objective price exceeds roughly 3.18 s, the subjective price is less than 1% greater than the objective price.

#### Linear versus logarithmic representation of subjective time

According to Eq 5, the subjective-price function contains within it a function, *f*_*t*_, that translates objective time into a subjective scale. There is a longstanding debate about whether this transformation is linear [31, 54] or logarithmic [55, 56]. The results reported here fit most gracefully with the linear account. The logarithm function is concave down: it changes most quickly over low values and decelerates continuously. In contrast, the best-fitting subjective-price function evaluated here, the sigmoidal-slope function, is concave up; that function is flat over low values and then accelerates until it approaches scalar growth. In order to reconcile logarithmic subjective-time with the results, the time-valuation function, *f*_*v*_, would have to invert the subjective-time function, *f*_*t*_, (i.e., by exponentiation) and then transform the result further to achieve the form of the sigmoidal-slope function. No such acrobatics are required of the linear-time account. Indeed, the x-intercept of a linear-time function could contribute to $P_{sub_{min}}$.

#### Working for reward versus waiting for reward

The results have potential implications that could extend well beyond the reward-mountain model and experiments on intracranial self-stimulation. Among these is the possibility that time spent spent ***working*** for reward is evaluated differently than time spent ***waiting*** for reward. A related distinction of potential interest concerns the consequences of having, or losing, agency over the time of reward delivery.

In the FCHT task, credit toward earning a reward accumulates as a result of the rat’s agency: The rat puts time on the work clock by actively holding down the lever. Performing this action brings the reward progressively closer in time, much like handling of a prey item by a forager (e.g., opening a shell) or travel to a new patch. The rat is also free to abandon holding down the lever. Thus, the response does not commit the rat to a period of passive waiting: the rat can switch into leisure activities at any time before the full price has been paid.

In contrast, the subject in a temporal discounting experiment has no control over time that elapses during the delay to reward delivery. Once the delay interval starts, the subject has already exercised the agency available to it by performing the operant response and now must wait helplessly until the programmed delay expires. By performing the operant response, the rat makes a commitment it cannot cancel: it must wait out the full delay. Could the exaggerated preference for immediacy that is expressed in hyperbolic delay discounting reflect the desire to minimize the duration of this binding commitment to passive waiting?

We suspect that differences between the evaluation of time spent working for and waiting for reward contributed to the superior performance of the sigmoidal-slope function in comparison to the subjective-price functions derived from discounting. Recall that the sigmoidal-slope function converges on the objective-price function quickly (within roughly 3 s in this study). After that time, the incremental increase in subjective price is constant, as Fig 8 shows. This is as predicted by the framework developed here. If the benefit of alternate activities remains stationary, the incremental opportunity cost of holding down the lever in the FCHT task is also deemed to be constant: it is simply the incremental value of leisure. In contrast, in the temporal-discounting accounts, the incremental cost of waiting, expressed as a proportion, varies as function of delay to reward delivery. This variation is flamboyant in the case of the exponential-price function.

It has been proposed that rewards are discounted differently by delay and physical effort [57, 58]. However, these studies do not address or manipulate opportunity costs, which are the subject of the present work. To our knowledge, the distinction proposed here between time spent working and waiting for reward has received little or no attention in the literatures on inter-temporal choice and reward valuation. We suggest that it could prove profitable to explore this distinction experimentally.

### Future directions

The conjecture that time spent working or waiting for reward is evaluated differently invites and demands a direct test. This could be done within the reward-mountain paradigm by interposing delays to reward delivery after the response requirement (work time) has been satisfied. Would a “classic” discounting function describe valuation of time spent waiting for rewards already earned [32, 36, 37], whereas the sigmoidal-slope function would continue to describe the valuation of time spent working for reward? An analogous head-to-head comparison could be carried out readily in human participants working and waiting for monetary rewards.

Sources of reward that aren’t under direct experimental control, such as the fruits of leisure activities, play a central role in Herrnstein’s depiction of single-operant responding [28, 29], in behavioral-economic accounts [59], in a recent reinforcement-learning model [60, 61], and in our account of performance on the FCHT schedule [15, 18, 23]. In contrast, such “background” rewards are typically omitted from explanations of temporal discounting. It would be interesting to explore the consequences of integrating such background sources of reward into accounts of inter-temporal choice.

Another issue that should be addressed in future work is the discrepancy between the portrayal of opportunity costs here and in accounts of operant performance derived from machine learning [60–62]. As in economic accounts [3, 4], opportunity cost is equated here with the highest-valued option forgone (a leisure activity), whereas in the accounts developed within the machine-learning tradition (and within optimal-foraging theory [63]), the opportunity cost reflects the average value of the environment as a whole, thus encompassing both the experimenter-controlled reward and the fruits of leisure. Moreover, the account presented here treats the benefits of leisure as a scalar function of leisure time, whereas more complex benefit-of-leisure functions have been explored in a promising manner by Niyogi and Dayan [60, 61]. Additional work will be required to determine the relative virtues and drawbacks of these different approaches and accounts. Exquisite experimental control, stability of performance over time, and very high data density make the intracranial self-stimulation paradigm a promising way to address such issues in future work.

## Supporting information

S1 File The Supporting-Information file contains text that a) specifies the units of subjective price, b) provides a derviation of the equation for the mid-range contour line, c) provides back-solutions of the subjective-price functions, and d) describes a computer problem that caused small deviations of the objective prices from the intended values (summarized in Table A). Six additional tables (Tables B-G) list the best-fitting parameter values for each of the rats). Another table (Table H) compares the fitted values of *P*_*obj*_*e*_ and *P*_*sub*_*e*_. Twenty-four figures are included: Figs A-F show time allocation as a function of the strength and cost of reward for each rat, Figs G-L show contour plots corresponding to the surfaces in Figs A-F, Figs M-R show comparisons, for each rat, between interpolated data points and pulse-frequency-versus-objective-price trade-off functions derived from the surface fits, and Figs S-X show, for each rat, the subjective-price functions obtained by fitting the four models.
(PDF)
