# Supplementary material for: Valuation of opportunity costs by rats working for rewarding electrical brain stimulation
Source: PLoS One. 2017 Aug 25;12(8):e0182120. doi: 10.1371/journal.pone.0182120 (PMC5571941; doi:10.1371/journal.pone.0182120)
Supplement: S1 File — The Supporting-Information file contains text that a) specifies the units of subjective price, b) provides a derviation of the equation for the mid-range contour line, c) provides back-solutions of the subjective-price functions, and d) describes a computer problem that caused small deviations of the objective prices from the intended values (summarized in Table A). Six additional tables (Tables B-G) list the best-fitting parameter values for each of the rats). Another table (Table H) compares the fitted values of Pobj_e and Psub_e. Twenty-four figures are included: Figs A-F show time allocation as a function of the strength and cost of reward for each rat, Figs G-L show contour plots corresponding to the surfaces in Figs A-F, Figs M-R show comparisons, for each rat, between interpolated data points and pulse-frequency-versus-objective-price trade-off functions derived from the surface fits, and Figs S-X show, for each rat, the subjective-price functions obtained by fitting the four models. (PDF) [file pone.0182120.s001.pdf]

Valuation of Opportunity Costs by Rats Working for Rewarding Electrical Brain Stimulation: Supporting Information

S1 File: Supplementary Information

The units of subjective price

To simplify the exposition in the main part of the paper, we did not provide definitions of the units for the variables. In particular, the units of subjective price were not defined. The purpose of this section is to provide that definition and to clarify how it fits into the reward-mountain model.

In the framework for the reward-mountain model, the period during which the lever is extended into the test cage is subdivided into time spent working for the experimenter-controlled reward (in this case, depressing a lever to obtain electrical brain stimulation), and time spent in alternate "leisure" activities such as grooming, resting, and exploring. Trial time in the FCHT paradigm is treated discontinuously as a series of "reward encounters" that begin upon extension of the lever and end when the reward is triggered. The reward encounters are separated by the black-out delays. It is assumed that a common currency is used to evaluate the reward and the leisure activities. We dub the units of this currency: "*utils*."

The function used in the reward-mountain model [1, 2] to describe time allocation within a reward encounter is the same as that used to describe time allocation within a trial:

$$T = T_{min} + \left[(T_{max} - T_{min}) \times \frac{{U_b}^a}{{U_b}^a + {U_e}^a} \right] \tag{S1}$$

where

$$\begin{aligned} a &= \text{the payoff-sensitivity exponent} \\ U_b &= \text{payoff from a train of rewarding brain stimulation} \\ U_e &= \text{payoff from the time spent in leisure activities during a} \\ &\quad\ \text{reward encounter} \\ T_{max} &= \text{maximal time allocation, and} \\ T_{min} &= \text{minimal time allocation.} \end{aligned}$$

It follows from Eq. S1 that when time allocation falls half-way between its minimal and maximal values (i.e., $T = T_{mid}$), U_b equals U_e. This makes intuitive the use of common units for the utilities of brain stimulation reward and "everything else" (the fruits of leisure activities).

In previous work [1, 2], we defined U_b as follows:

$$U_b = \frac{R}{(1+\xi) \times P_{sub}} \tag{S2}$$

where

$$\begin{aligned} P_{sub} &= \text{the subjective price of the stimulation train} \\ R &= \text{the intensity of the subjective reward signal triggered by the stimulation, and} \\ (1+\xi) &= \text{the subjective rate of exertion experienced while holding down the lever.} \end{aligned}$$

Eq. S2 makes clear that the utility of the rewarding brain stimulation incorporates both its benefits and two types of costs: opportunity and effort. To reflect this, we use specific units for each of the inputs and for the output.

We assign R the units, *hedons*, and subjective exertion the units, *oomphs*. Thus, $(1+\xi)$, the rate of subjective exertion entailed in holding down the lever, has the units, *oomphs* s^{-1}. We assign P_{sub} the complementary units, *s oomph*$^{-1}$. In this way, P_{sub}, which represents the subjective opportunity cost of the reward, is evaluated in units compatible with $(1+\xi)$, which expresses the rate at which the subjective effort cost of the reward grows as a function of work time. Defined in this way, P_{sub} gives the number of seconds of required work time that discount the reward by the same amount as an effort cost of 1 *oomph*.

Denominating P_{sub} in units of *s oomph*$^{-1}$ makes intuitive sense. In the reward-mountain model [1], P_{sub} can be expressed as:

$$P_{sub\_e} = \frac{R_{max}}{(1+\xi) \times U_e} \tag{S3}$$

where

$$\begin{aligned} R_{max} &= \text{the maximum attainable reward intensity, and} \\ P_{sub\_e} &= \text{the subjective price at which time allocated to working for a maximal reward is halfway between } T_{min} \text{ and } T_{max}. \end{aligned}$$

Imagine that the value of leisure activities were boosted by providing the rat with a set of interesting toys [3]. The cost of forgoing leisure will now have grown. Denominated in this more valuable currency, fewer seconds of work produce an opportunity cost that discounts the reward to the same degree as one *oomph* of subjective effort, and the rat will no longer be willing to devote as much time as before to pursuit of a maximal brain-stimulation reward.

Reward intensity grows as a function of stimulation strength and train duration [4, 5]. The study by Sonnenschein et al. [5] is consistent with the view that it is the peak reward intensity achieved during the pulse train that determines time allocation. Thus, R and R_{max} should be defined in terms of this peak value. For consistency, U_e should also be defined according to the principle of "representation by exemplar" [6], which means that the value of a prototypical moment is used to represent an entire episode (leisure bout).

Several additional scaling constants are required for consistency. For example, the function, f_v, (Eq. 4 in the main text) translates time spent holding down the lever (units: s) into the associated opportunity cost (units: $s\ oomph^{-1}$), thus requiring a scaling constant to bring this about. Similarly, Eq. S2 requires a scaling constant to transform $hedons$ into $utils$:

$$U_b = K_u \times \frac{R}{(1+\xi) \times P_{sub}} \tag{S4}$$

where

K_u = a scaling constant with the units $utils\ hedon^{-1}$.

Eq. S3 then becomes:

$$P_{sub\_e} = K_u \times \frac{R_{max}}{(1+\xi) \times U_e} \tag{S5}$$

K_u must also be incorporated into U_e so that the peak reward intensity experienced during performance of leisure is also translated into $utils$.

Derivation of the equation for the mid-range contour line

The equation for the reward-mountain model [1] can be rearranged as follows:

$$\frac{T - T_{min}}{T_{max} - T_{min}} = \frac{\left(\frac{F^g}{F^g + F^g_{hm}}\right)^a}{\left(\frac{F^g}{F^g + F^g_{hm}}\right)^a + \left(\frac{P_{sub}(P_{obj})}{P_{sub\_e}(P_{obj\_e})}\right)^a} \tag{S6}$$

where

a = the payoff-sensitivity exponent. This parameter determines the steepness of the mountain along the price axis.

F_{hm} = the pulse frequency that produces half-maximal reward intensity.

g = the intensity-growth exponent. This parameter determines the steepness of the intensity-growth function and contributes to the steepness of the mountain along the pulse-frequency axis.

P_{obj} = the objective price (opportunity cost).

P_{obj_e} = the objective price at which the time allocation to pursuit of a maximal reward falls halfway between T_{max} and T_{min}.

$P_{sub}(P_{obj})$ = the subjective price corresponding to the objective price, P_{obj}.

$P_{sub\_e}(P_{obj\_e})$ = the subjective price at which time allocation to pursuit of a maximal reward falls halfway between T_{max} and T_{min}.

T_{max} = maximal time allocation, and

T_{min} = minimal time allocation.

When time allocation falls midway between T_{max} and T_{min},

$$\frac{T_{mid} - T_{min}}{T_{max} - T_{min}} = 0.5 \quad \text{(S7)}$$

where

T_{mid} = the T value mid-way between T_{max} and T_{min}

It follows that when $T = T_{mid}$,

$$\frac{F^g_{mid}}{F^g_{mid} + F^g_{hm}} = \frac{P_{sub}(P_{obj})}{P_{sub\_e}(P_{obj\_e})} \quad \text{(S8)}$$

where

F_{mid} = the pulse frequency at which T falls mid-way between T_{max} and T_{min} for each value of P_{sub}.

Thus

$$F^g_{mid} = F^g_{hm} \times \frac{P_{sub}(P_{obj})}{P_{sub\_e}(P_{obj\_e}) - P_{sub}(P_{obj})} \quad \text{(S9)}$$

To plot the mid-range contour line in double logarithmic coordinates, Eq. S9 is transformed as follows:

$$Log_{10}(F_{mid}) = Log_{10}(F_{hm}) + \left[\frac{1}{g} \times Log_{10}\left(\frac{P_{sub}(P_{obj})}{P_{sub\_e}(P_{obj\_e}) - P_{sub}(P_{obj})}\right)\right] \quad \text{(S10)}$$

The values of P_{sub} are obtained by passing the values of P_{obj} employed in the experiment through the subjective-price function. The parameters of that function as well as the values of F_{hm}, g, and P_{sub_e} are obtained by fitting the reward-mountain model.

Back-solutions of the subjective price functions

Objective-price function:

$$P_{obj} = P_{sub} \quad \text{(S11)}$$

Sigmoidal-slope function:

$$P_{obj} = P_{sub_{min}} + P_{sub_{bend}} \times \ln\left[-1 + e^{\left(\frac{P_{sub} - P_{sub_{min}}}{P_{sub_{bend}}}\right)}\right] \quad \text{(S12)}$$

Linear-price function:

$$P_{obj} = \frac{-1 + P_{sub}}{K_h} \quad \text{(S13)}$$

Exponential-price function:

$$P_{obj} = \frac{ln(P_{sub})}{K_x} \tag{S14}$$

Deviation of the objective price from the intended values

The program controlling the experiment measures work time by counting ticks of the computer's system clock. In the venerable personal computers used in this study, the system clock runs at a frequency of 18.206 Hz (rounded to three decimal places), which corresponds to a period of 54.925 ms. To translate the prices specified by the experimenter (the nominal price) into clock ticks, the specified value was multiplied by the clock frequency and the result rounded to the nearest integer. The rounding error is one source of the discrepancy between the price specified by the experimenter (the nominal price) and the price actually paid by the rat (Table A). The second source is the asynchrony between the rat's behaviour and the system clock. The moment that the lever is depressed falls in the interval demarcated by successive clock ticks. On average, the offset of that time from the nearest tick equals one half of the clock period. Consequently, the time that the lever must be depressed in order to trigger a stimulation train will, on average, be one half of a clock period greater than the specified number of ticks. This is why the average work time required to trigger a stimulation train (the objective price) exceeds the nominal price (Table A). For prices greater than 1 s, the error is less than 2%. The larger errors at shorter prices are unlikely to be consequential because the subjective-price function is flat, or nearly so over this range.

Table A. Deviation of average prices from their nominal values

nominal set Price	average objective price
0.125	0.137
0.250	0.302
0.500	0.522
1.000	1.016
2.000	2.005
4.000	4.037
8.000	8.047
16.000	16.011

Deviation of the price specified by the experimenter (nominal price) from the average cumulative work time (objective price) actually required to earn a stimulation train. The asynchrony between the rat's behaviour and the system clock of the computer creates a bias that makes the objective price somewhat greater than the nominal price. Rounding error contributes to the discrepancy.

Table B. Best fitting parameter values for rat F3

Function	Parameter	Fitted estimate	CB low	CB high	CB width
Objective					
	a	4·76	2·60	25·00	22·40
	g	9·72	5·80	11·19	5·40
	$Log_{10}(F_{hm})$	1·74	1·72	1·81	0·09
	$Log_{10}(P_{sub\_e})$	0·97	0·92	1·04	0·12
	T_{max}	0·97	0·96	0·98	0·02
	T_{min}	0·21	0·18	0·22	0·04
Sigmoidal					
	a	3·47	3·01	4·03	1·02
	g	2·02	1·65	2·39	0·74
	$Log_{10}(F_{hm})$	1·93	1·88	2·00	0·12
	$Log_{10}(P_{sub\_e})$	1·14	1·09	1·19	0·10
	$Log_{10}(P_{sub_{min}})$	0·33	0·22	0·43	0·21
	$P_{sub_{bend}}$	0·53	0·11	1·00	0·89
	T_{max}	1·00	1·00	1·00	0·00
	T_{min}	0·13	0·12	0·15	0·03
Linear					
	a	14·88	7·34	25·00	17·66
	g	1·12	0·79	1·50	0·71
	K_h	0·05	0·02	0·10	0·08
	$Log_{10}(F_{hm})$	1·38	1·02	1·67	0·65
	$Log_{10}(P_{sub\_e})$	0·24	0·12	0·40	0·28
	T_{max}	1·00	1·00	1·00	0·00
	T_{min}	0·14	0·13	0·15	0·03
Exponential					
	a	1·83	0·79	5·17	4·38
	g	3·60	1·28	5·81	4·53
	K_x	0·38	0·10	0·65	0·55
	$Log_{10}(F_{hm})$	2·06	1·87	2·17	0·30
	$Log_{10}(P_{sub\_e})$	1·93	0·61	3·00	2·39
	T_{max}	1·00	1·00	1·00	0·00
	T_{min}	0·13	0·12	0·14	0·03

CB = 95% confidence band.

Table C. Best fitting parameter values for rat F9

Function	Parameter	Fitted estimate	CB low	CB high	CB width
Objective					
	a	3·51	2·44	6·05	3·62
	g	7·18	6·17	10·53	4·36
	$Log_{10}(F_{hm})$	1·81	1·77	1·83	0·06
	$Log_{10}(P_{sub\_e})$	0·99	0·92	1·03	0·12
	T_{max}	0·95	0·93	0·96	0·03
	T_{min}	0·20	0·17	0·25	0·08
Sigmoidal					
	a	2·98	2·61	3·45	0·84
	g	2·78	2·35	3·20	0·85
	$Log_{10}(F_{hm})$	1·88	1·85	1·92	0·06
	$Log_{10}(P_{sub\_e})$	1·09	1·06	1·13	0·07
	$Log_{10}(P_{sub_{min}})$	0·25	0·17	0·34	0·17
	$P_{sub_{bend}}$	0·29	0·02	0·71	0·69
	T_{max}	1·00	0·99	1·00	0·01
	T_{min}	0·10	0·08	0·12	0·04
Linear					
	a	17·31	7·76	25·00	17·24
	g	1·34	0·99	1·77	0·79
	K_h	0·04	0·02	0·10	0·08
	$Log_{10}(F_{hm})$	1·35	1·11	1·64	0·53
	$Log_{10}(P_{sub\_e})$	0·19	0·11	0·35	0·24
	T_{max}	1·00	0·99	1·00	0·01
	T_{min}	0·11	0·09	0·13	0·04
Exponential					
	a				
	g				
	K_x				
	$Log_{10}(F_{hm})$				
	$Log_{10}(P_{sub\_e})$				
	T_{max}				
	T_{min}				

CB = 95% confidence band. The values for the exponential-price model are omitted because that fit failed to converge.

Table D. Best fitting parameter values for rat F12

Function	Parameter	Fitted estimate	CB low	CB high	CB width
Objective					
	a	3·63	2·72	4·47	1·75
	g	8·01	6·26	9·38	3·12
	$Log_{10}(F_{hm})$	1·94	1·92	1·96	0·04
	$Log_{10}(P_{sub\_e})$	0·84	0·81	0·88	0·07
	T_{max}	0·96	0·95	0·97	0·02
	T_{min}	0·20	0·16	0·23	0·07
Sigmoidal					
	a	2·85	2·66	3·06	0·40
	g	3·18	2·94	3·43	0·50
	$Log_{10}(F_{hm})$	2·00	1·98	2·02	0·04
	$Log_{10}(P_{sub\_e})$	0·93	0·91	0·96	0·05
	$Log_{10}(P_{sub_{min}})$	0·11	0·10	0·14	0·04
	$P_{sub_{bend}}$	0·02	0·01	0·02	0·01
	T_{max}	1·00	1·00	1·00	0·00
	T_{min}	0·12	0·10	0·13	0·03
Linear					
	a	7·41	5·26	11·18	5·92
	g	2·07	1·68	2·52	0·84
	K_h	0·14	0·07	0·23	0·16
	$Log_{10}(F_{hm})$	1·77	1·63	1·86	0·22
	$Log_{10}(P_{sub\_e})$	0·34	0·22	0·47	0·25
	T_{max}	1·00	1·00	1·00	0·00
	T_{min}	0·12	0·10	0·13	0·04
Exponential					
	a				
	g				
	K_x				
	$Log_{10}(F_{hm})$				
	$Log_{10}(P_{sub\_e})$				
	T_{max}				
	T_{min}				

CB = 95% confidence band. The values for the exponential-price model are omitted because that fit failed to converge.

Table E. Best fitting parameter values for rat F16

Function	Parameter	Fitted estimate	CB low	CB high	CB width
Objective					
	a	1·99	1·52	2·91	1·39
	g	9·42	7·43	24·08	16·65
	$Log_{10}(F_{hm})$	1·85	1·75	1·89	0·14
	$Log_{10}(P_{sub\_e})$	0·92	0·86	0·98	0·11
	T_{max}	0·94	0·90	0·96	0·05
	T_{min}	0·20	0·18	0·23	0·05
Sigmoidal					
	a	2·35	2·06	2·72	0·66
	g	3·06	2·66	3·53	0·87
	$Log_{10}(F_{hm})$	1·88	1·83	1·93	0·10
	$Log_{10}(P_{sub\_e})$	0·97	0·93	1·00	0·07
	$Log_{10}(P_{sub_{min}})$	0·33	0·22	0·42	0·20
	$P_{sub_{bend}}$	0·21	0·03	0·52	0·49
	T_{max}	1·00	1·00	1·00	0·00
	T_{min}	0·15	0·13	0·17	0·04
Linear					
	a	15·44	5·79	25·00	19·21
	g	2·01	1·65	2·49	0·84
	K_h	0·04	0·02	0·10	0·08
	$Log_{10}(F_{hm})$	1·44	1·22	1·68	0·46
	$Log_{10}(P_{sub\_e})$	0·14	0·06	0·29	0·23
	T_{max}	1·00	1·00	1·00	0·00
	T_{min}	0·16	0·15	0·18	0·03
Exponential					
	a	16·80	2·65	25·00	22·35
	g	2·06	1·62	2·97	1·35
	K_x	0·04	0·01	0·16	0·14
	$Log_{10}(F_{hm})$	1·44	1·21	1·87	0·66
	$Log_{10}(P_{sub\_e})$	0·17	0·06	0·61	0·55
	T_{max}	1·00	1·00	1·00	0·00
	T_{min}	0·16	0·15	0·18	0·04

CB = 95% confidence band.

Table F. Best fitting parameter values for rat F17

Function	Parameter	Fitted estimate	CB low	CB high	CB width
Objective					
	a	1·26	1·06	1·52	0·47
	g	7·85	6·84	9·29	2·45
	$Log_{10}(F_{hm})$	1·85	1·82	1·88	0·06
	$Log_{10}(P_{sub\_e})$	1·04	0·98	1·09	0·11
	T_{max}	0·98	0·96	1·00	0·04
	T_{min}	0·15	0·13	0·17	0·04
Sigmoidal					
	a	1·99	1·67	2·67	1·00
	g	3·76	3·09	4·40	1·31
	$Log_{10}(F_{hm})$	1·83	1·78	1·87	0·09
	$Log_{10}(P_{sub\_e})$	0·99	0·95	1·02	0·07
	$Log_{10}(P_{sub_{min}})$	0·22	0·10	0·34	0·24
	$P_{sub_{bend}}$	0·72	0·07	2·50	2·43
	T_{max}	1·00	1·00	1·00	0·00
	T_{min}	0·12	0·10	0·13	0·03
Sigmoidal FB					
	a	1·91	1·69	2·16	0·47
	g	3·83	3·35	4·37	1·02
	$Log_{10}(F_{hm})$	1·84	1·81	1·87	0·05
	$Log_{10}(P_{sub\_e})$	0·99	0·95	1·03	0·07
	$Log_{10}(P_{sub_{min}})$	0·24	0·13	0·33	0·21
	T_{max}	1·00	1·00	1·00	0·00
	T_{min}	0·11	0·10	0·13	0·03
Linear					
	a	4·66	2·83	8·56	5·73
	g	2·76	2·16	3·35	1·19
	K_h	0·14	0·05	0·26	0·21
	$Log_{10}(F_{hm})$	1·68	1·52	1·78	0·26
	$Log_{10}(P_{sub\_e})$	0·37	0·18	0·56	0·38
	T_{max}	1·00	1·00	1·00	0·00
	T_{min}	0·13	0·11	0·14	0·03
Exponential					
	a	0·71	0·45	1·51	1·07
	g	8·32	4·31	11·31	7·01
	K_x	0·60	0·25	0·82	0·58
	$Log_{10}(F_{hm})$	1·93	1·86	1·98	0·12
	$Log_{10}(P_{sub\_e})$	2·30	0·93	3·00	2·07
	T_{max}	1·00	1·00	1·00	0·00
	T_{min}	0·12	0·11	0·14	0·03

CB = 95% confidence band; FB = “fixed bend”.

Table G. Best fitting parameter values for rat F18

Function	Parameter	Fitted estimate	CB low	CB high	CB width
Objective					
	a	3·91	2·68	7·82	5·15
	g	7·49	6·02	10·00	3·98
	$Log_{10}(F_{hm})$	1·71	1·65	1·74	0·09
	$Log_{10}(P_{sub\_e})$	1·03	0·97	1·08	0·11
	T_{max}	0·95	0·93	0·96	0·04
	T_{min}	0·14	0·11	0·16	0·05
Sigmoidal					
	a	4·28	3·02	7·06	4·04
	g	2·66	1·66	3·50	1·83
	$Log_{10}(F_{hm})$	1·78	1·69	1·83	0·14
	$Log_{10}(P_{sub\_e})$	1·14	1·10	1·18	0·08
	$Log_{10}(P_{sub_{min}})$	0·33	0·15	0·58	0·43
	$P_{sub_{bend}}$	1·24	0·02	3·59	3·57
	T_{max}	0·98	0·98	0·99	0·01
	T_{min}	0·09	0·08	0·10	0·02
Linear					
	a	20·86	8·77	25·00	16·23
	g	1·38	1·06	1·82	0·75
	K_h	0·04	0·02	0·09	0·07
	$Log_{10}(F_{hm})$	1·29	1·15	1·59	0·44
	$Log_{10}(P_{sub\_e})$	0·19	0·13	0·38	0·25
	T_{max}	0·98	0·98	0·99	0·01
	T_{min}	0·09	0·08	0·10	0·02
Exponential					
	a				
	g				
	K_x				
	$Log_{10}(F_{hm})$				
	$Log_{10}(P_{sub\_e})$				
	T_{max}				
	T_{min}				

CB = 95% confidence band. The values for the exponential-price model are omitted because that fit failed to converge.

Table H. Comparison of P_{obj_e} and P_{sub_e}

Rat	Parameter	Objective	Sigmoidal-Slope	Sigmoidal-Slope FB	Linear	Exponential
F03						
	P_{obj_e}	9.30	13.72		14.12	11.59
	P_{sub_e}	9.30	13.72		1.73	85.32
F09						
	P_{obj_e}	9.72	12.29		12.57	
	P_{sub_e}	9.72	12.29		1.56	
F12						
	P_{obj_e}	6.87	8.48		8.52	
	P_{sub_e}	6.87	8.48		2.21	
F16						
	P_{obj_e}	8.40	9.27		9.15	9.15
	P_{sub_e}	8.40	9.27		1.37	1.47
F17						
	P_{obj_e}	10.85	9.75	9.73	9.55	8.77
	P_{sub_e}	10.85	9.75	9.73	2.33	198.31
F18						
	P_{obj_e}	10.64	13.72		14.39	
	P_{sub_e}	10.64	13.72		1.54	

P_{obj_e} and P_{sub_e} are, respectively, the objective and subjective prices at which time allocation falls halfway between its minimal and maximal values. Blank cells indicate that the fit did not converge. “FB” refers to the “fixed-bend” fit of the sigmoidal-slope function with the value of the $P_{sub_{bend}}$ parameter set to 0.5. The estimates of P_{obj_e} and P_{sub_e} are identical, or nearly so, in the case of the sigmoidal-slope function, indicating that this function has already converged on the objective-price function. In contrast, highly discrepant estimates of P_{obj_e} and P_{sub_e} are produced by the linear- and exponential-price functions derived from temporal-discounting accounts. The discrepancy reflects increasing divergence of the subjective prices produced by these functions from the corresponding objective prices.

The main figures show data from Rat F16. The supplementary figures provide the data from the remaining subjects. The data from Rat F16 are included in the supplementary figures as well to facilitate comparison.

Figures A-F. Time allocation as a function of the strength and cost of reward for all six rats. The colored symbols represent the proportion of trial time allocated to reward seeking as a function of price and pulse frequency. The corresponding legend and contour plots are presented in Figs. G-L. Each of the fitted surfaces is defined by one of the four subjective-price functions.

Figures G-L. Contour plots corresponding to the surfaces in Figs. A-F. Time allocation is represented by the grey level, as shown in the bar at the upper right. Each colored symbol represents a tested pair of price and pulse-frequency values (i.e., a row of a sampling matrix); each color-shape combination denotes a different pseudo-sweep. The horizontally oriented series of blue squares represents the price pseudo-sweep, whereas the diagonally oriented series of green circles represents the radial pseudo-sweep. All the remaining series are pulse-frequency pseudo-sweeps carried

out at different prices. The vertical blue line represents the fitted value of the P_{obj_e} location parameter, whereas the horizontal red line represents the fitted value of the F_{hm} location parameter. The colored bands surrounding the location-parameter lines are 95% confidence intervals.

Figures M-R. Comparison between interpolated data points and pulse-frequency-versus-objective-price trade-off functions derived from the surface fits. The solid line is the contour in Figs. S1-A:F representing mid-range time allocation (half-way between T_{min} and T_{max}). The corresponding data points were interpolated by means of spline fits to the data from the pulse-frequency, price, and radial pseudo-sweeps.

Figures S-X. The subjective-price functions obtained by fitting the four models. The dashed lines are the subjective-price functions corresponding to the contours in Figs. M-R representing mid-range time allocation (half-way between T_{min} and T_{max}). These functions were computed by back-solving for $P_{sub}(P_{obj})$, given the fitted values of F_{hm}, g, and $P_{sub\_e}(P_{obj\_e})$ and the values of the two independent variables at each point along the contour line in Figs. M-R. The data points were transformed in the same manner.

References

1. Breton YA, Mullett A, Conover K, Shizgal P. Validation and extension of the reward-mountain model. Frontiers in Behavioral Neuroscience. 2013;7:125.
2. Hernandez G, Breton YA, Conover K, Shizgal P. At what stage of neural processing does cocaine act to boost pursuit of rewards? PLoS ONE. 2010;5(11).
3. Petry N, Heyman G. Rat Toys, Reinforcers, and Response Strength: An Examination of the Re Parameter in Herrnstein's Equation. Behavioural Processes. 1997;39:39–52.
4. Simmons JM, Gallistel CR. Saturation of subjective reward magnitude as a function of current and pulse frequency. Behavioral Neuroscience. 1994;108(1):151–160.
5. Sonnenschein B, Conover K, Shizgal P. Growth of brain stimulation reward as a function of duration and stimulation strength. Behavioral Neuroscience. 2003;117(5):978–994.
6. Kahneman D. Evaluation by Moments: Past and Future. In: Kahneman D, Tversky A, editors. Choices, Values, and Frames. Cambridge University Press; 2000. p. 693–708.

Rat F03

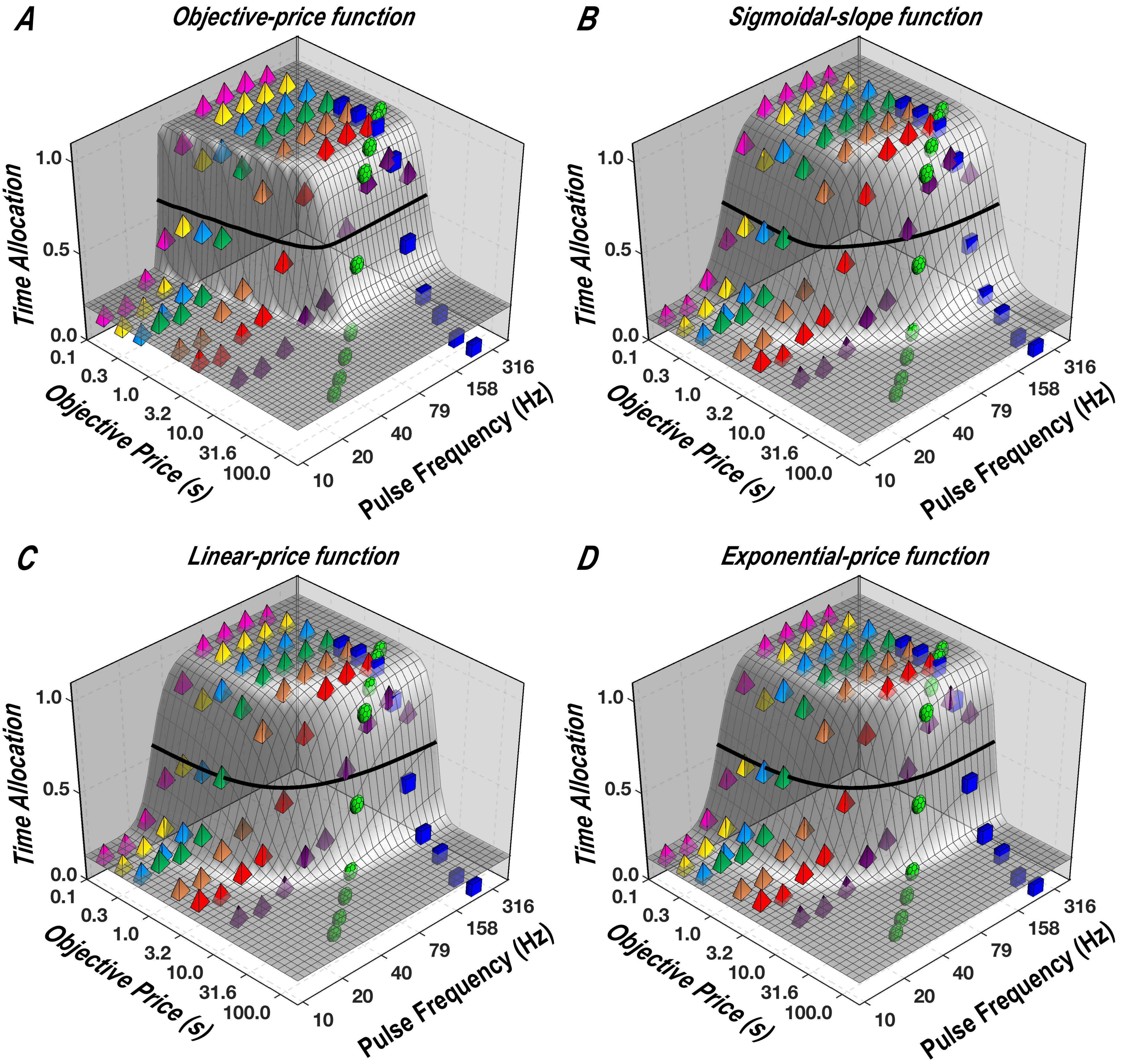

Figure A

Rat F09

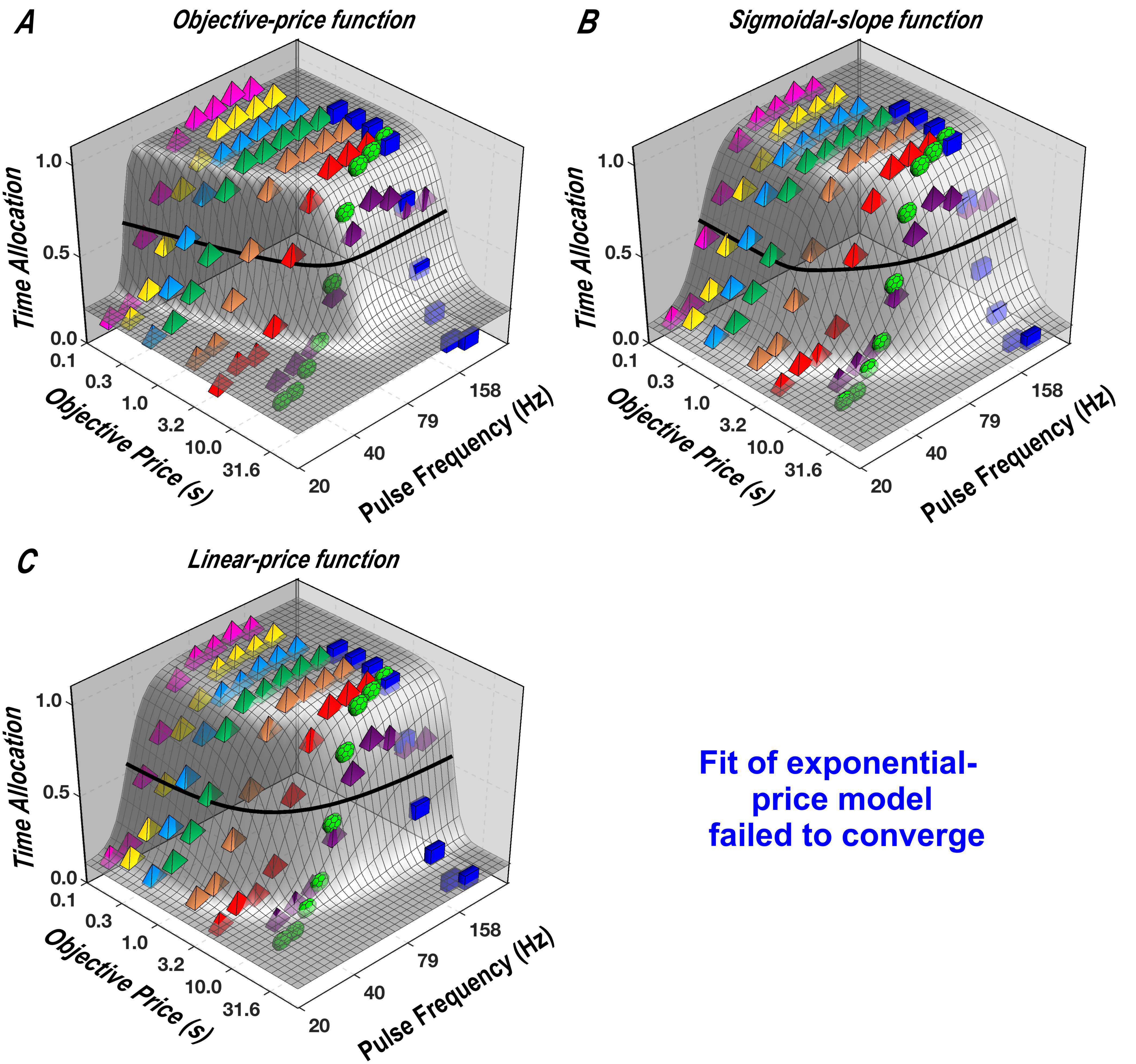

Figure B

Rat F12

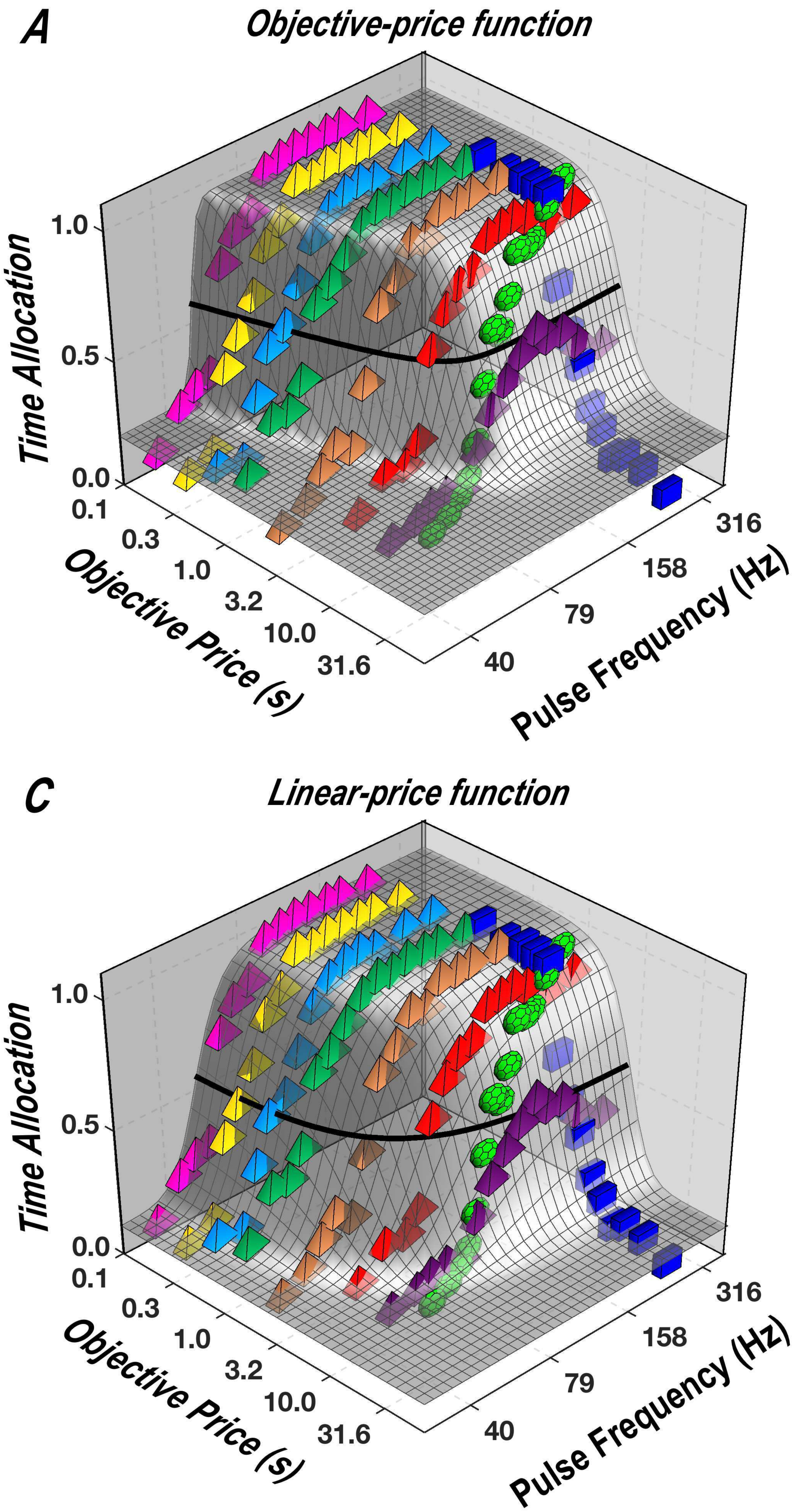

Fit of exponential-price model failed to converge

Figure C

Rat F16

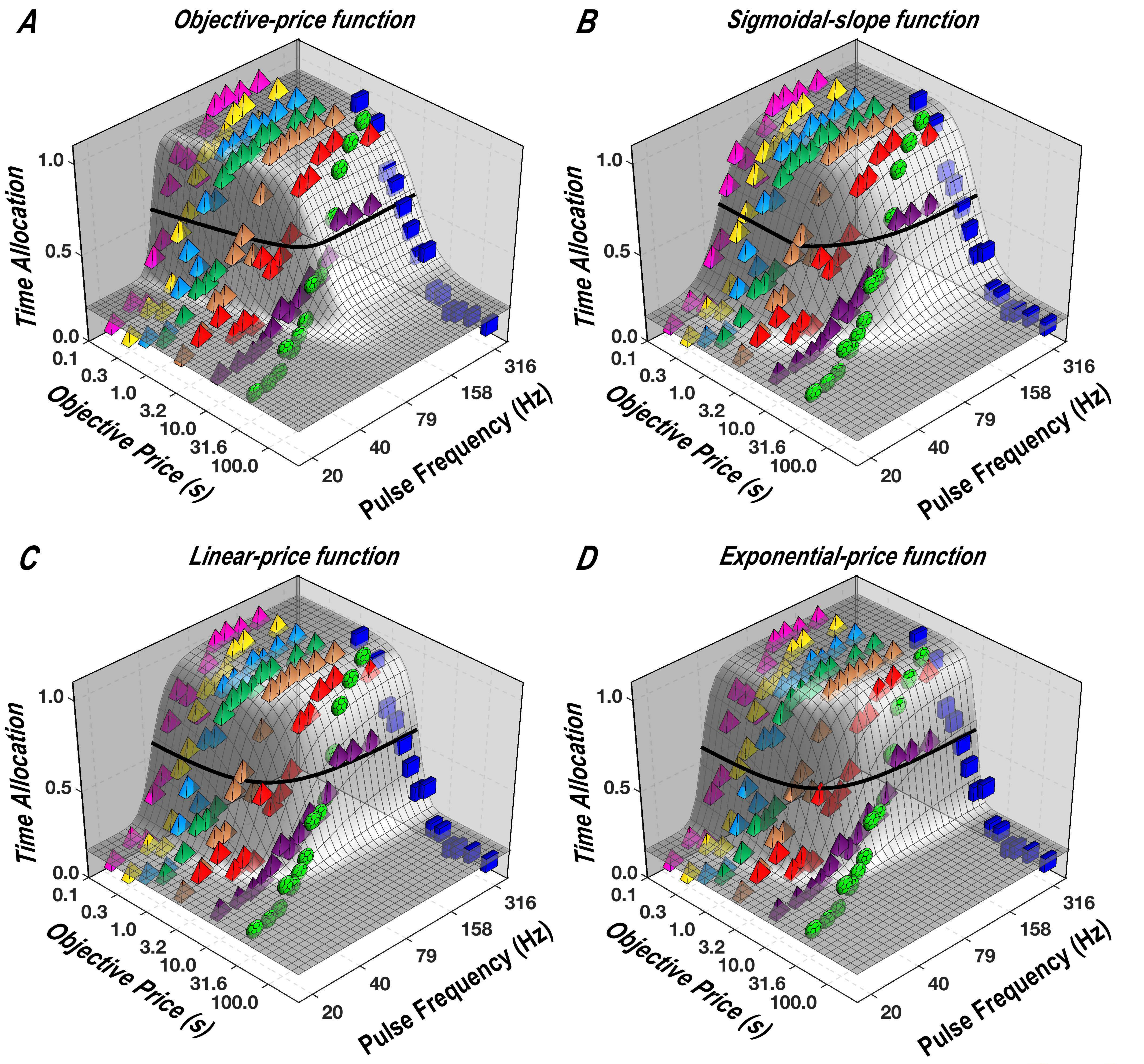

Figure D

Rat F17

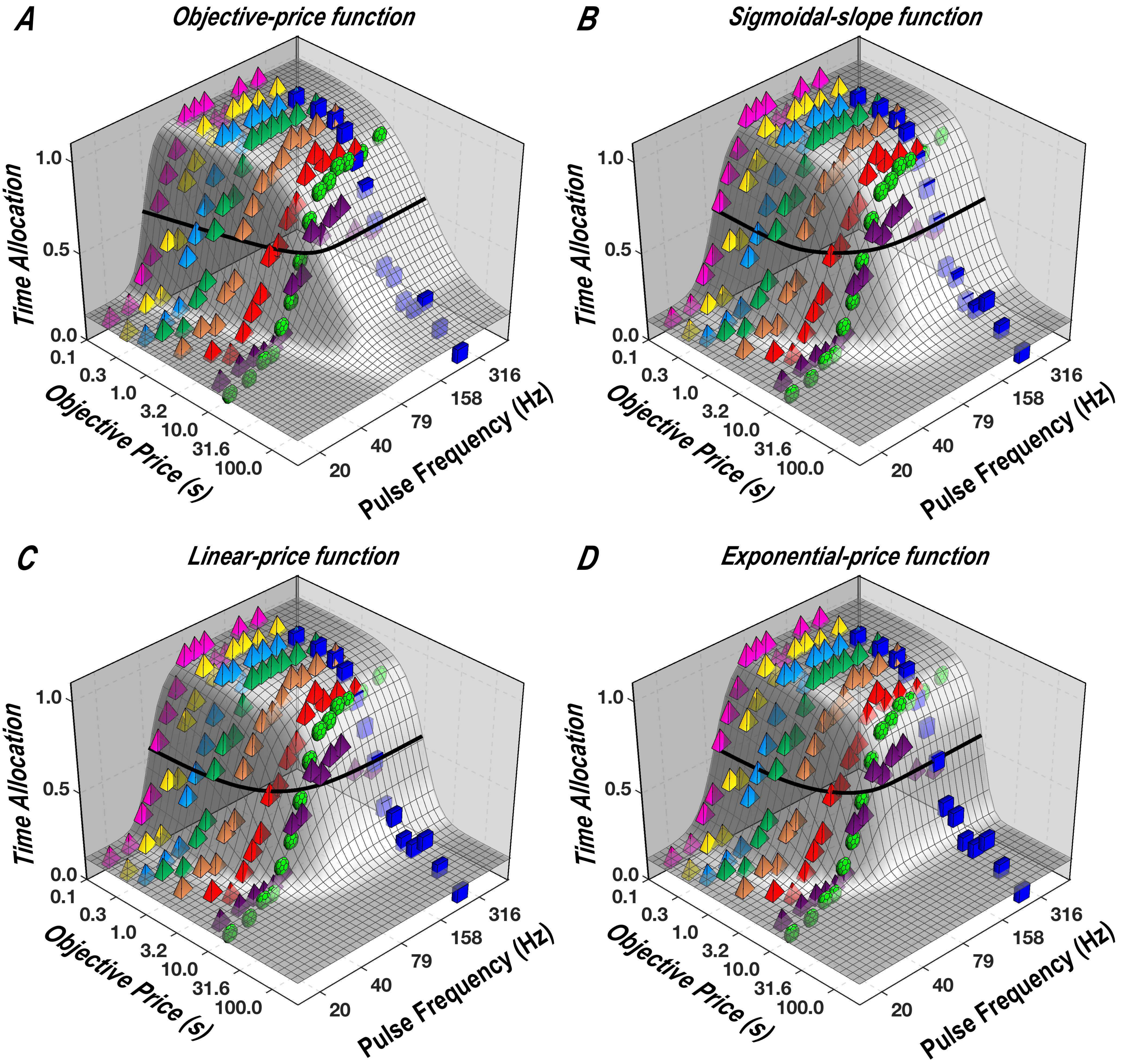

Figure E

Rat F18

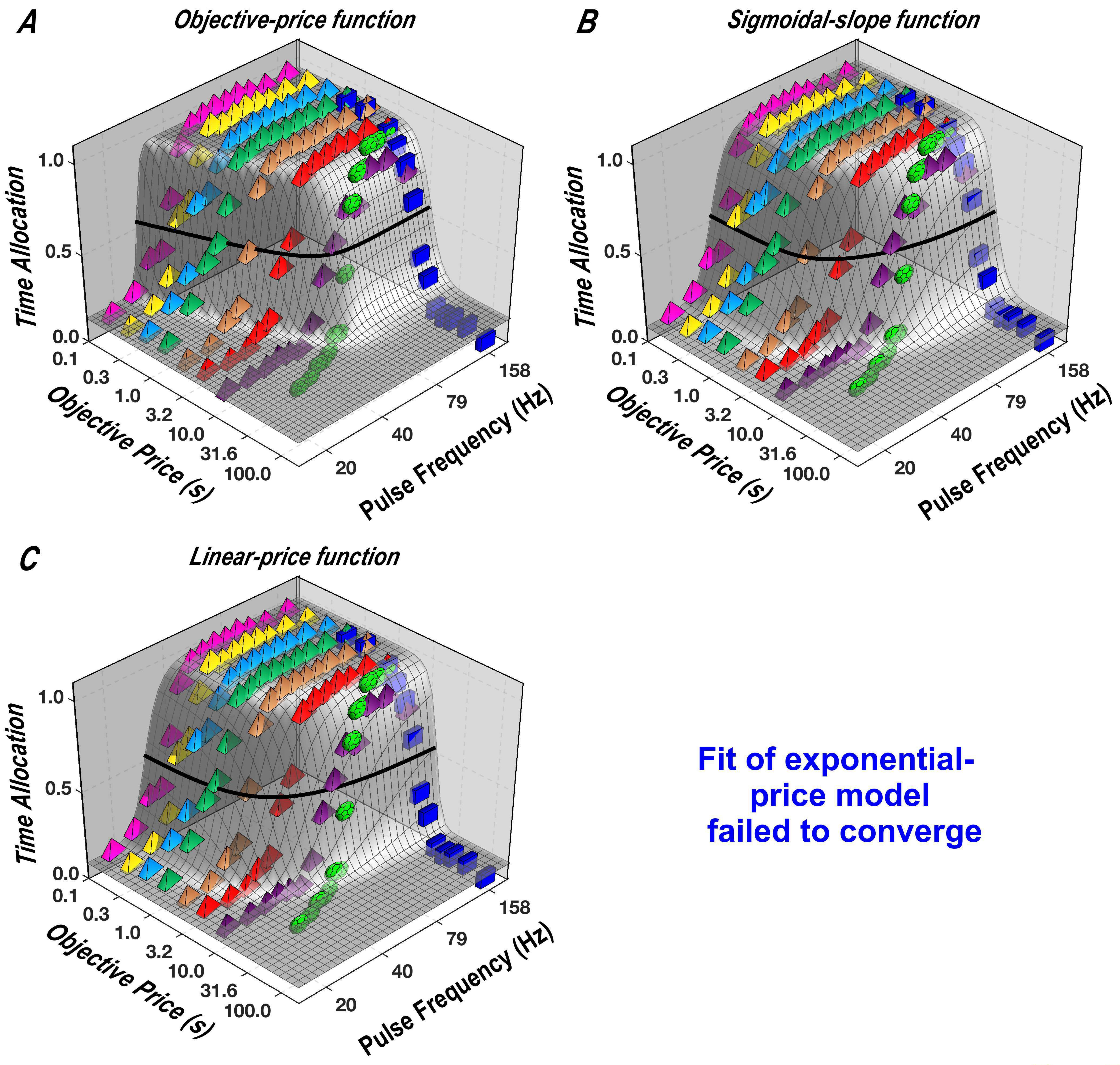

Figure F

Rat F03

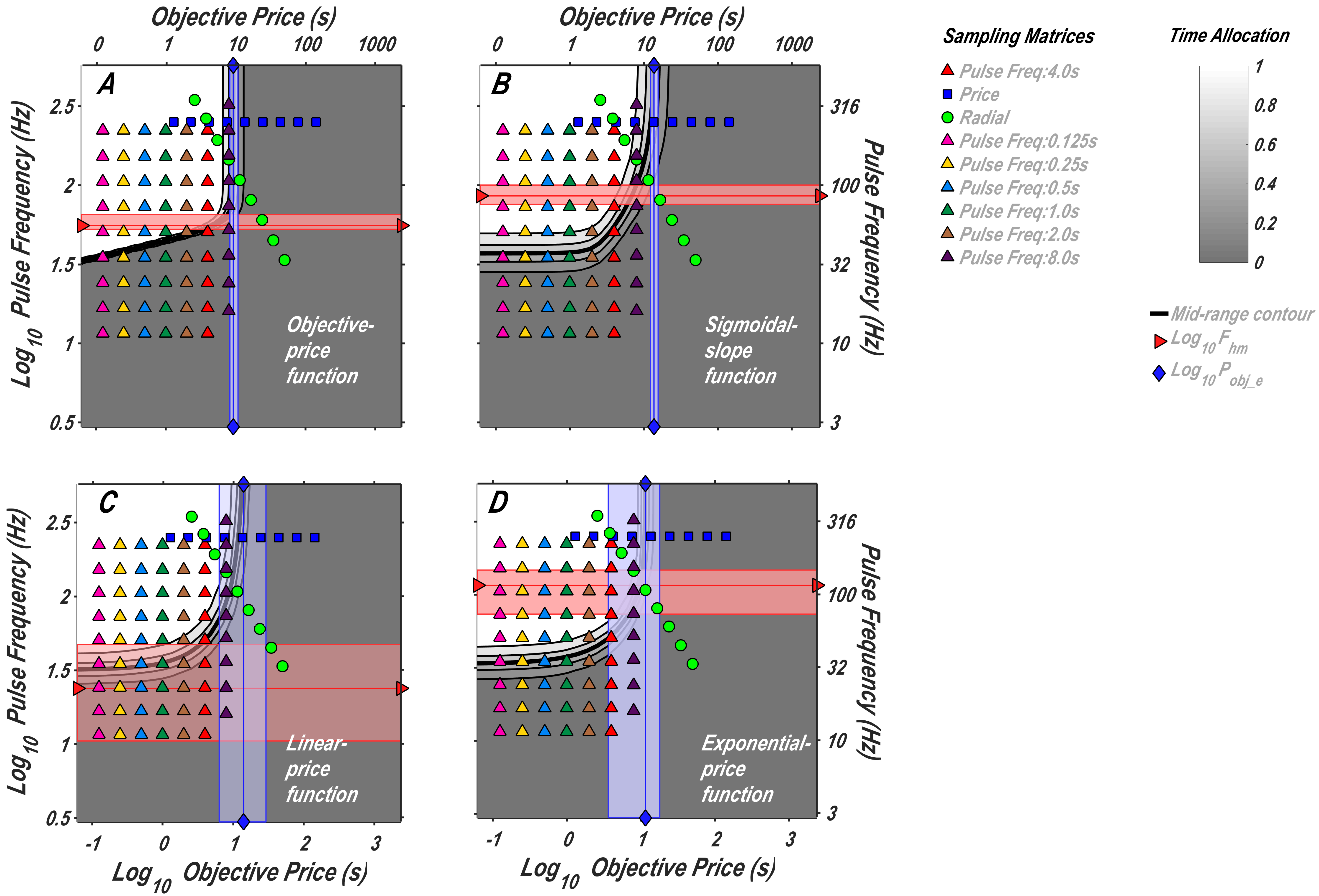

Figure G

Rat F09

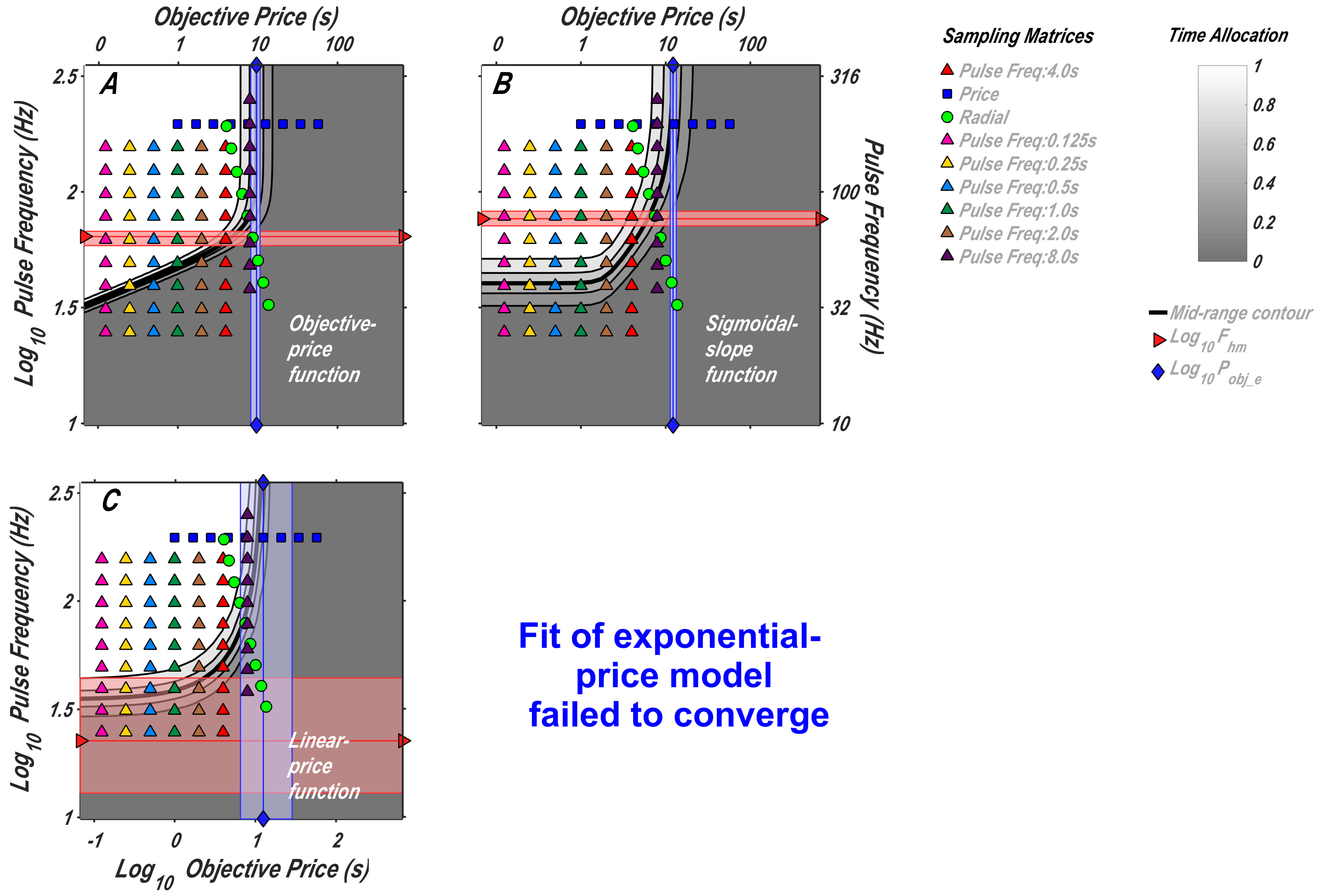

Fit of exponential-price model failed to converge

Figure H

Rat F12

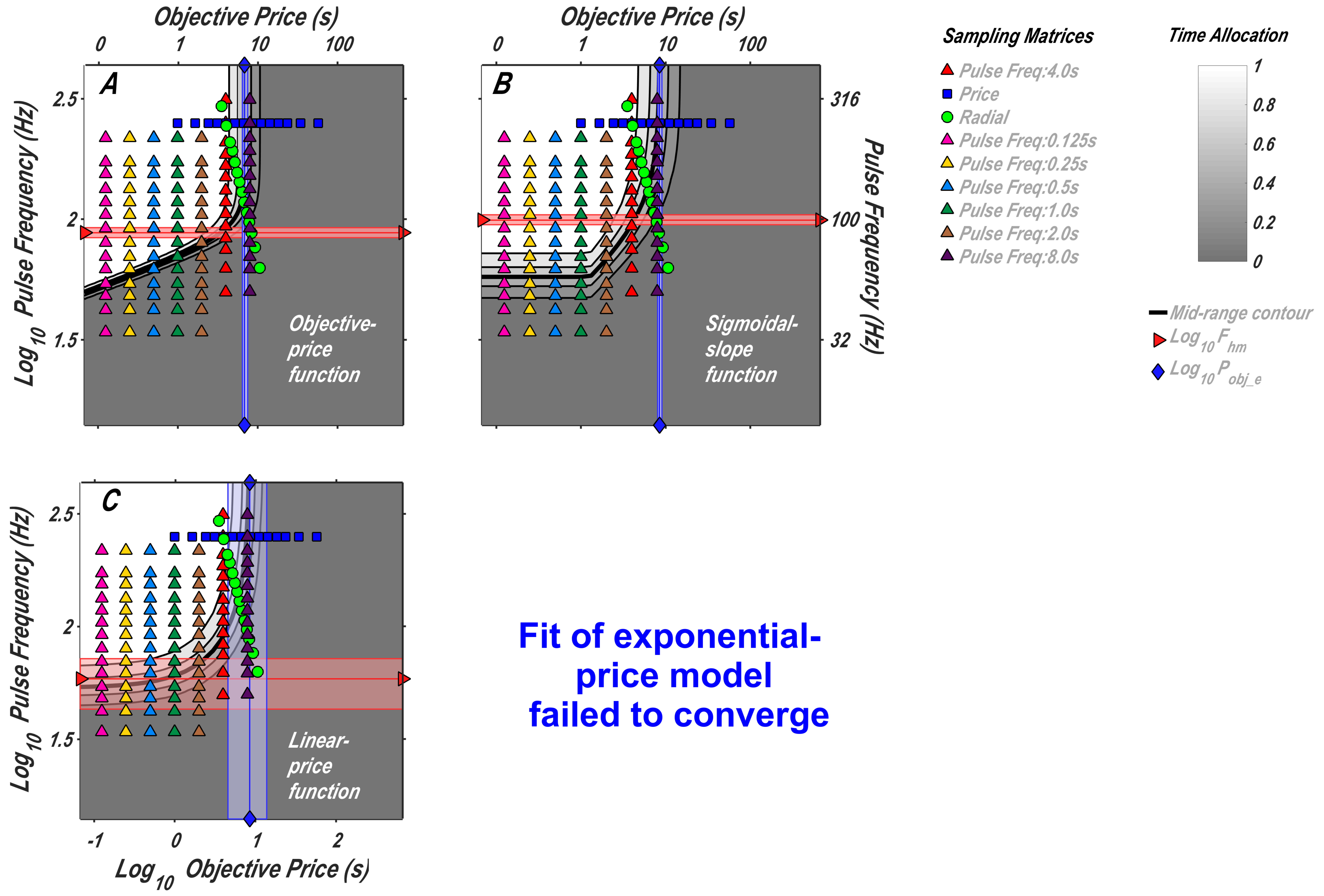

Figure I

Rat F16

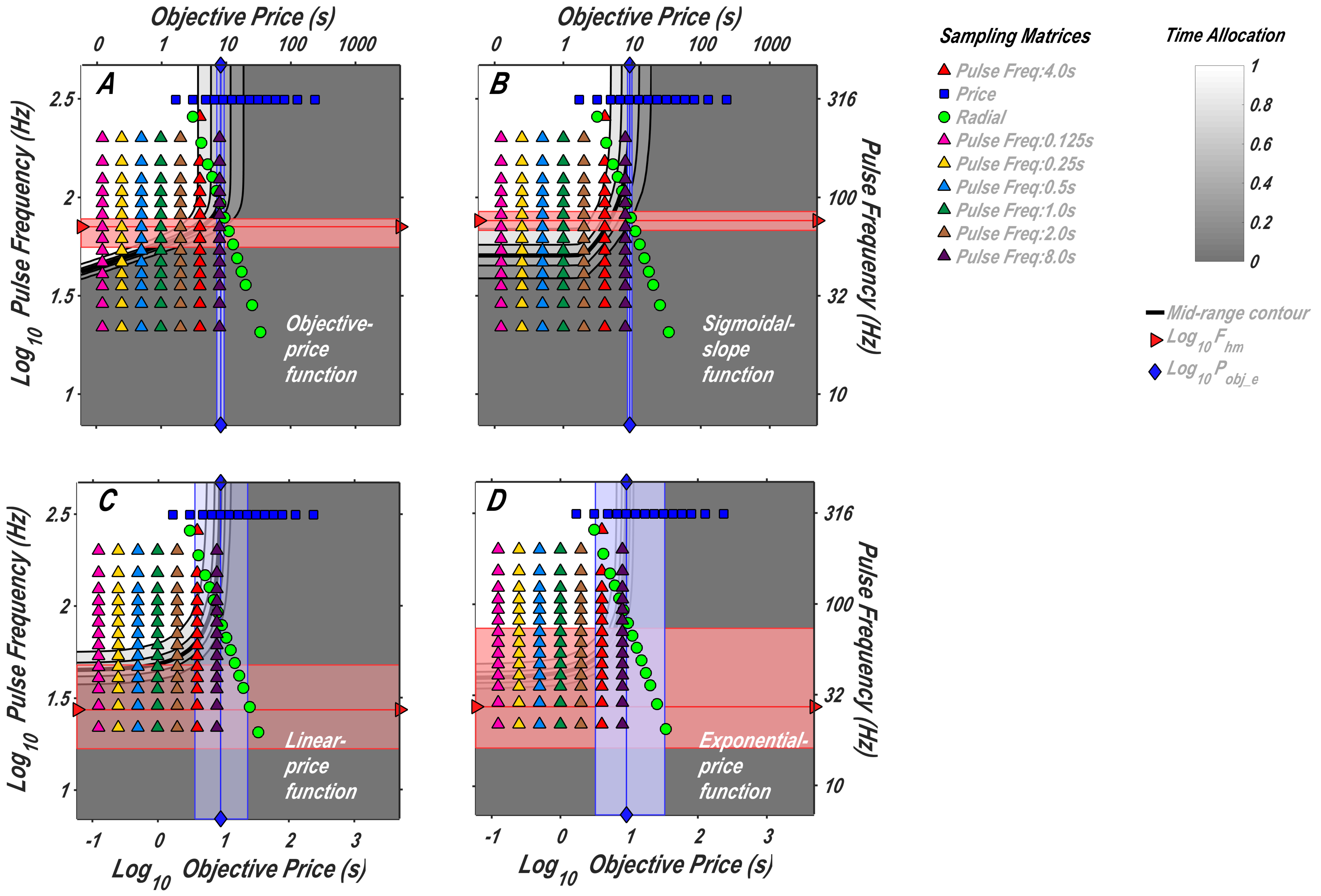

Figure J

Rat F17

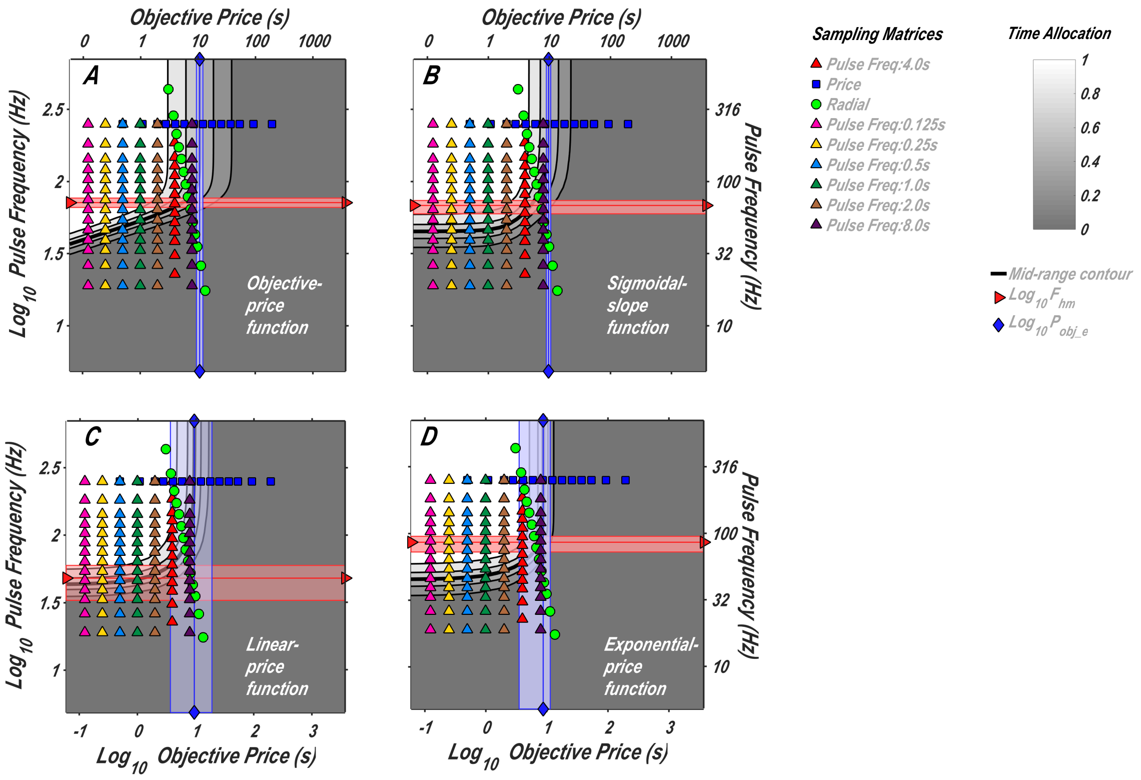

Figure K

Rat F18

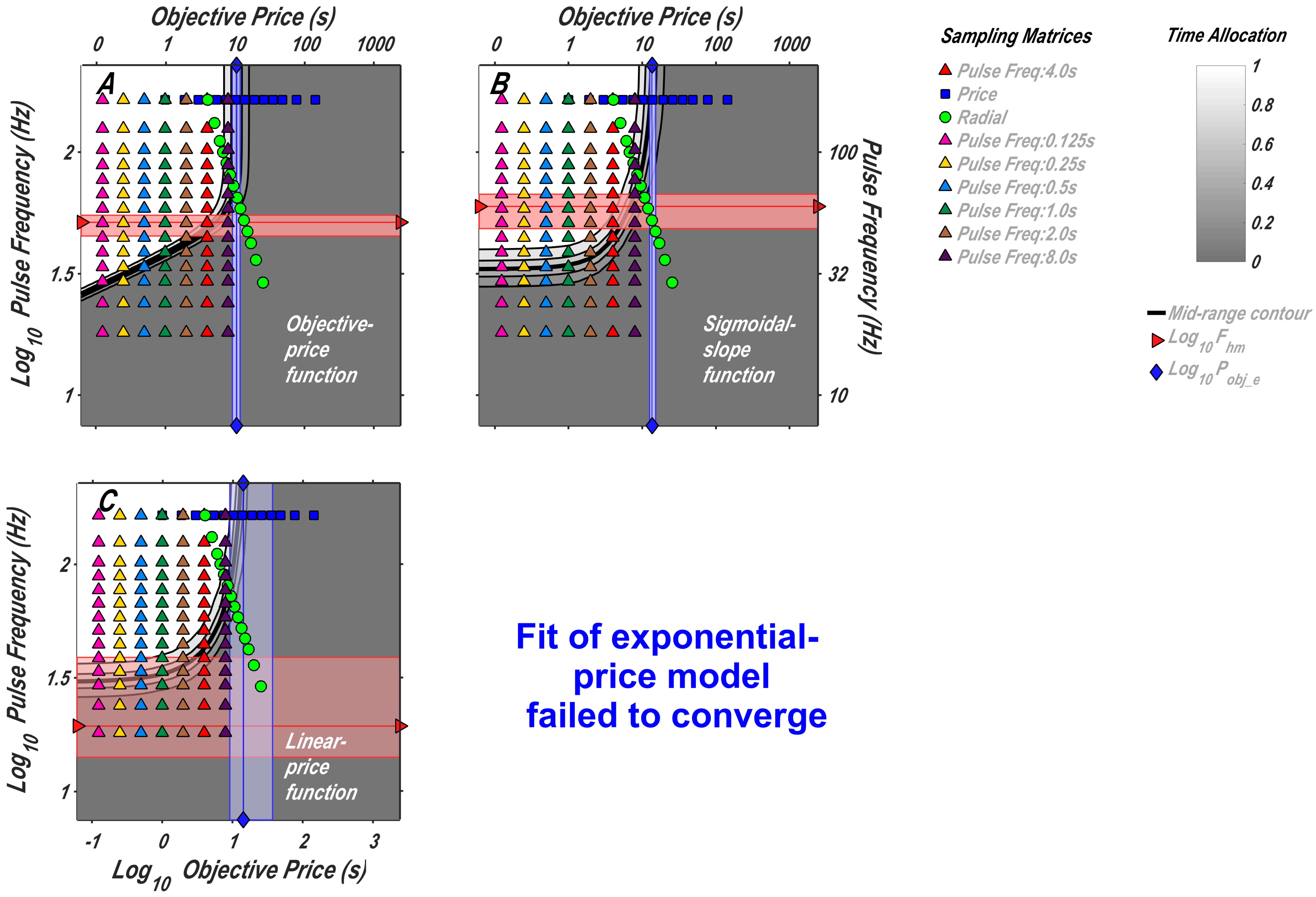

Fit of exponential-price model failed to converge

Figure L

Rat F03

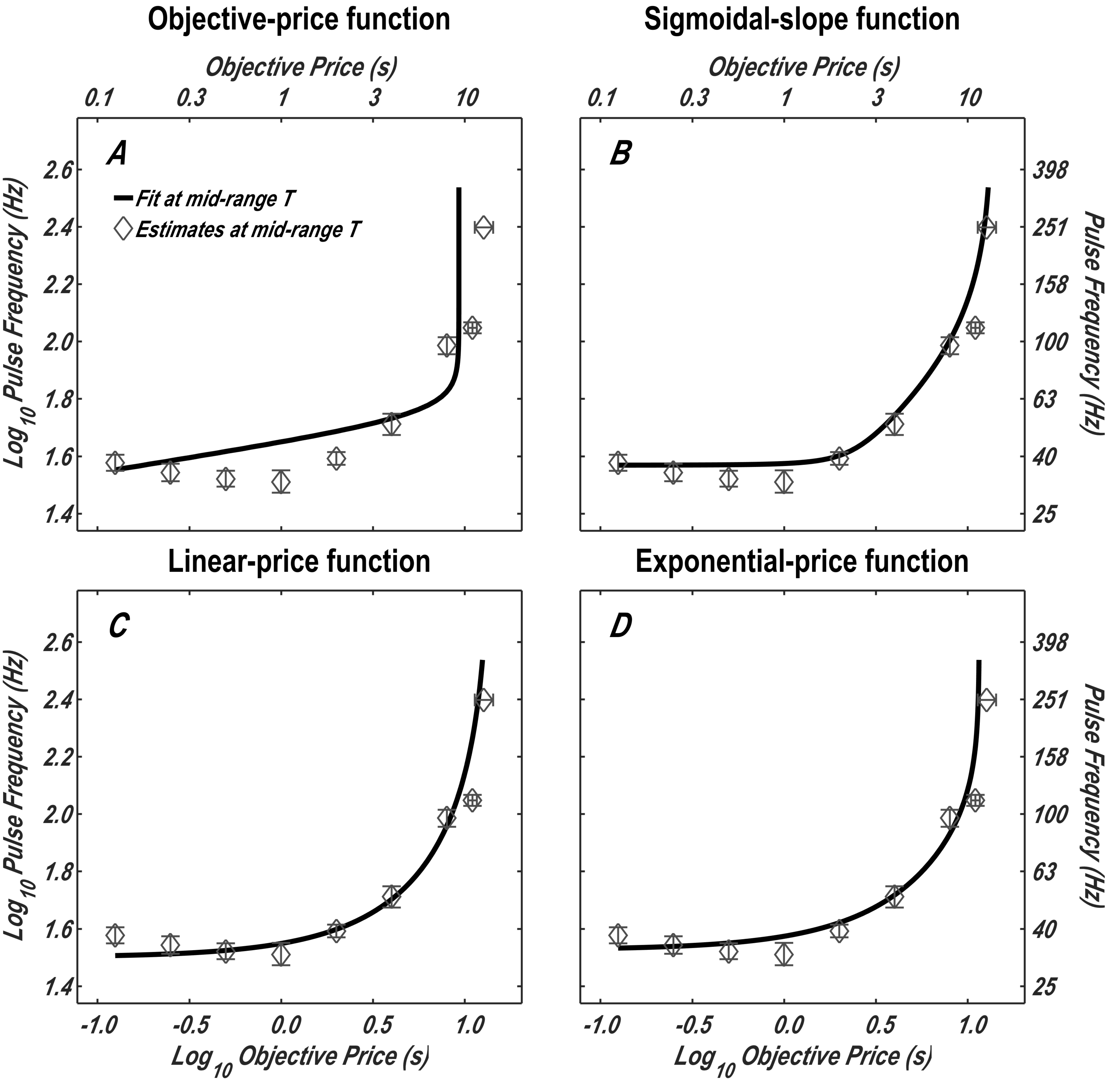

Figure M

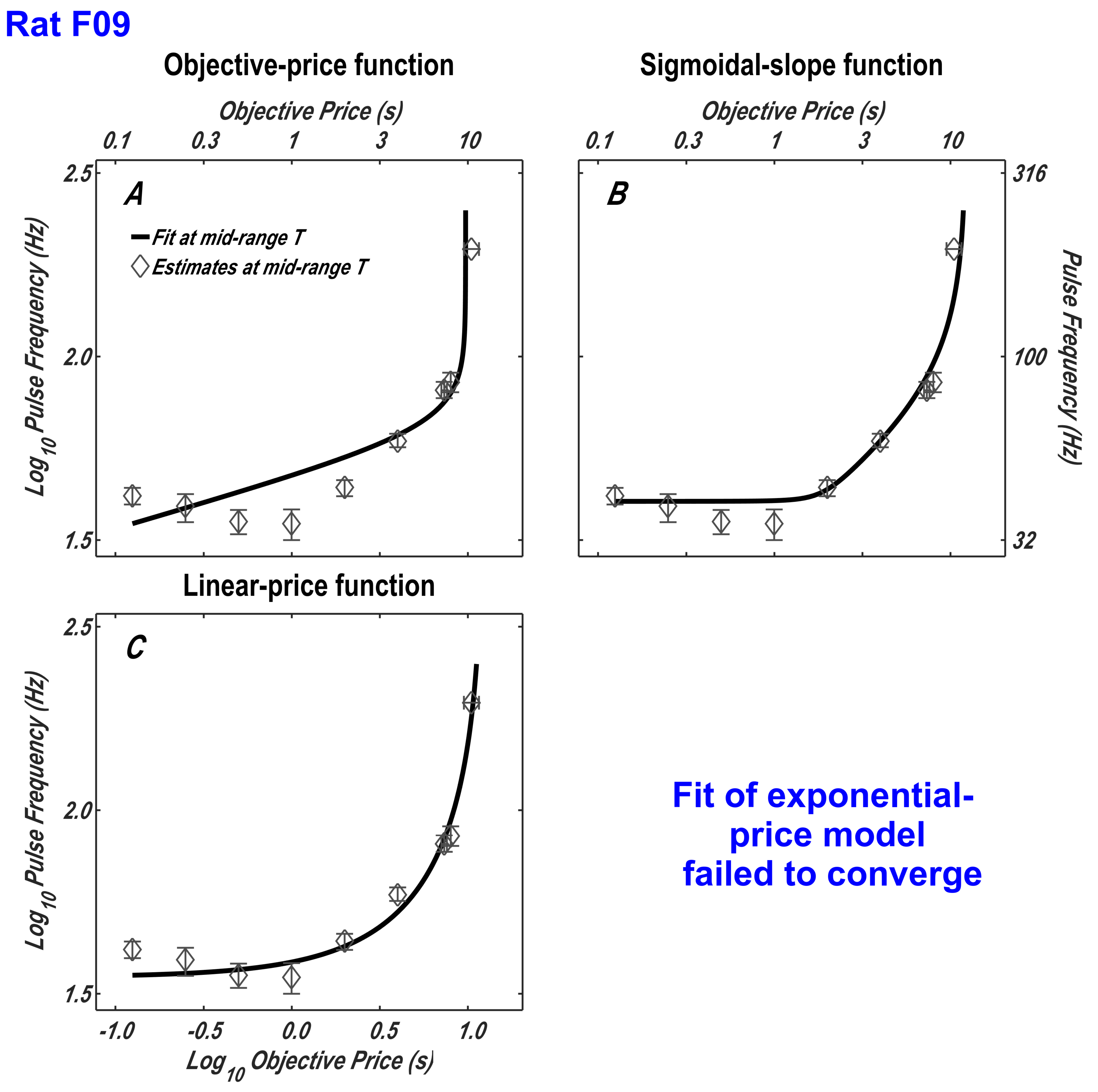

Rat F09
Objective-price function
Sigmoidal-slope function
Objective Price (s)
0.1
0.3
1
3
10
316
100
32
Pulse Frequency (Hz)
2.5
2.0
1.5
Log10 Pulse Frequency (Hz)
A
B
C
Fit at mid-range T
Estimates at mid-range T
Linear-price function
-1.0
-0.5
0.0
0.5
1.0
Log10 Objective Price (s)
Fit of exponential-price model failed to converge

Figure N

Rat F12

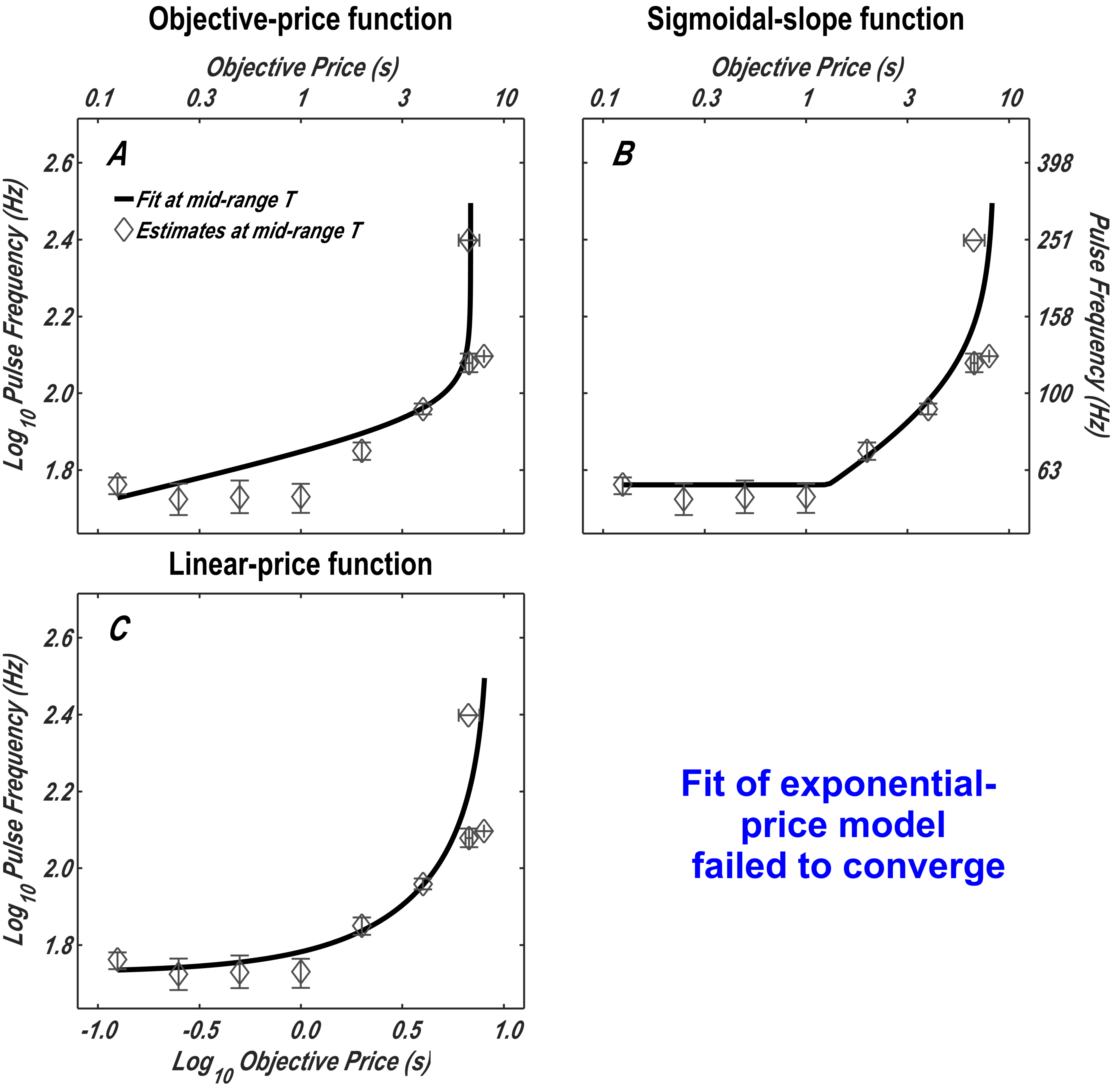

Fit of exponential-price model failed to converge

Figure O

Rat F16

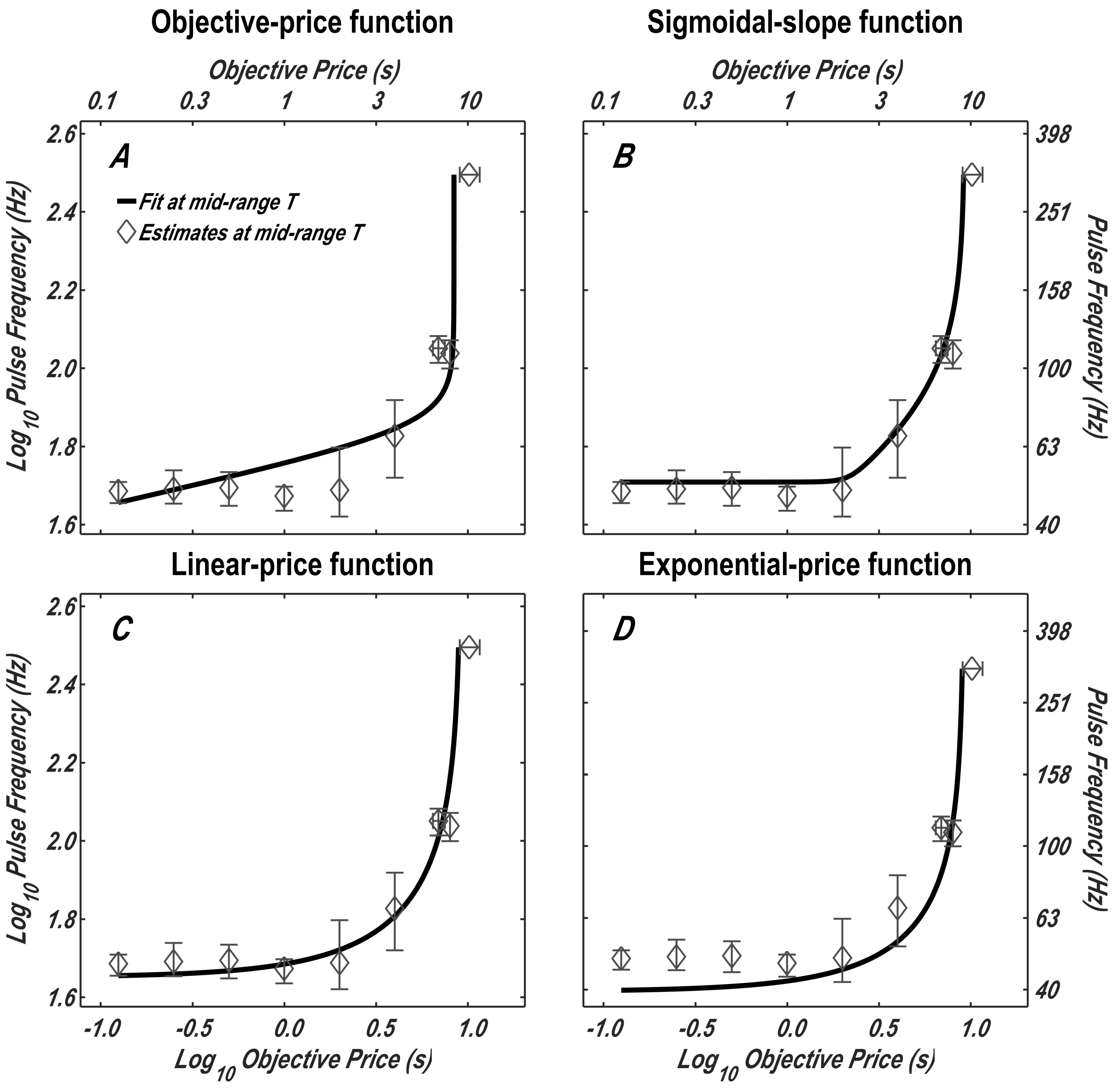

Figure P

Rat F17

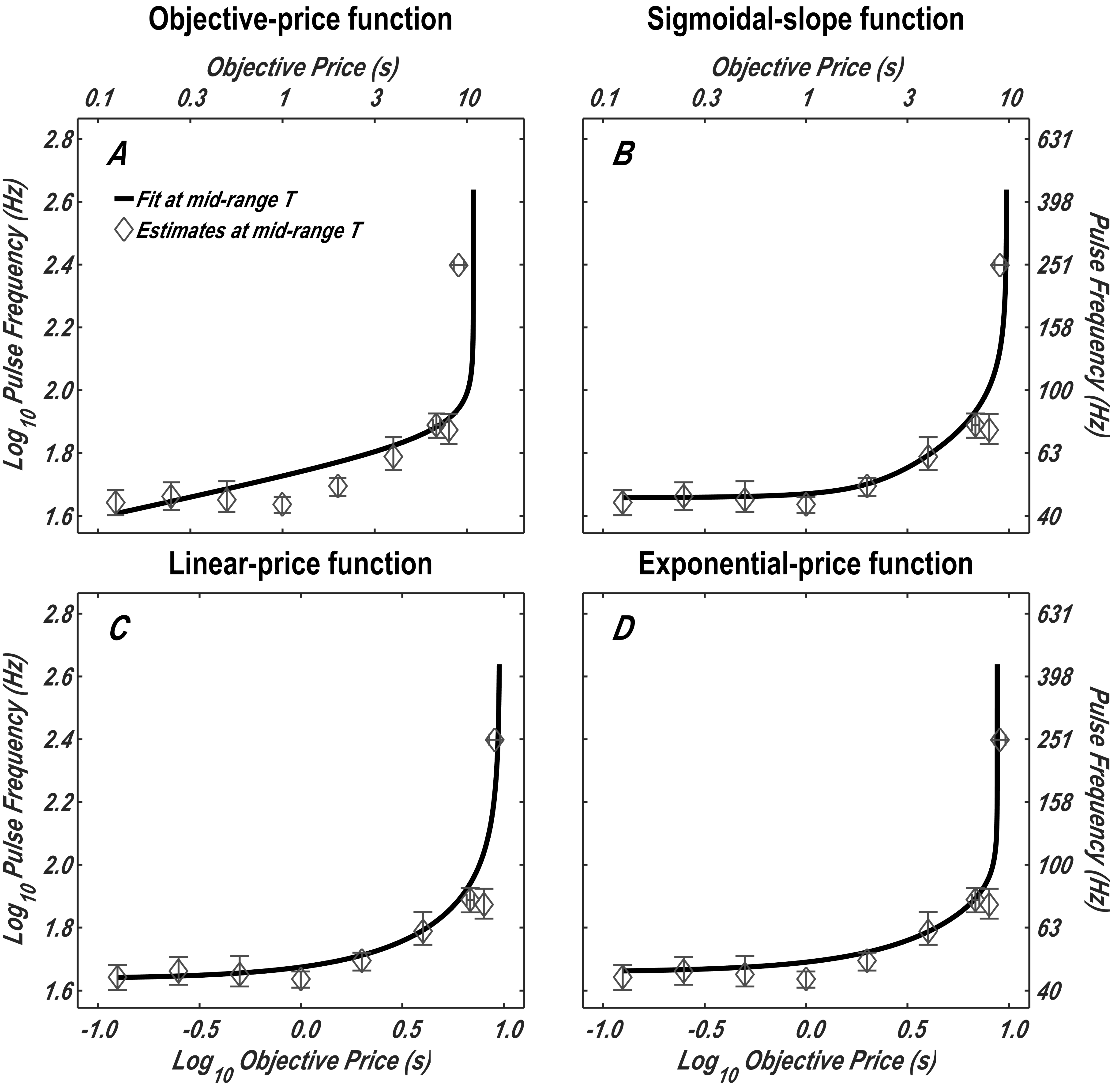

Figure Q

Rat F18

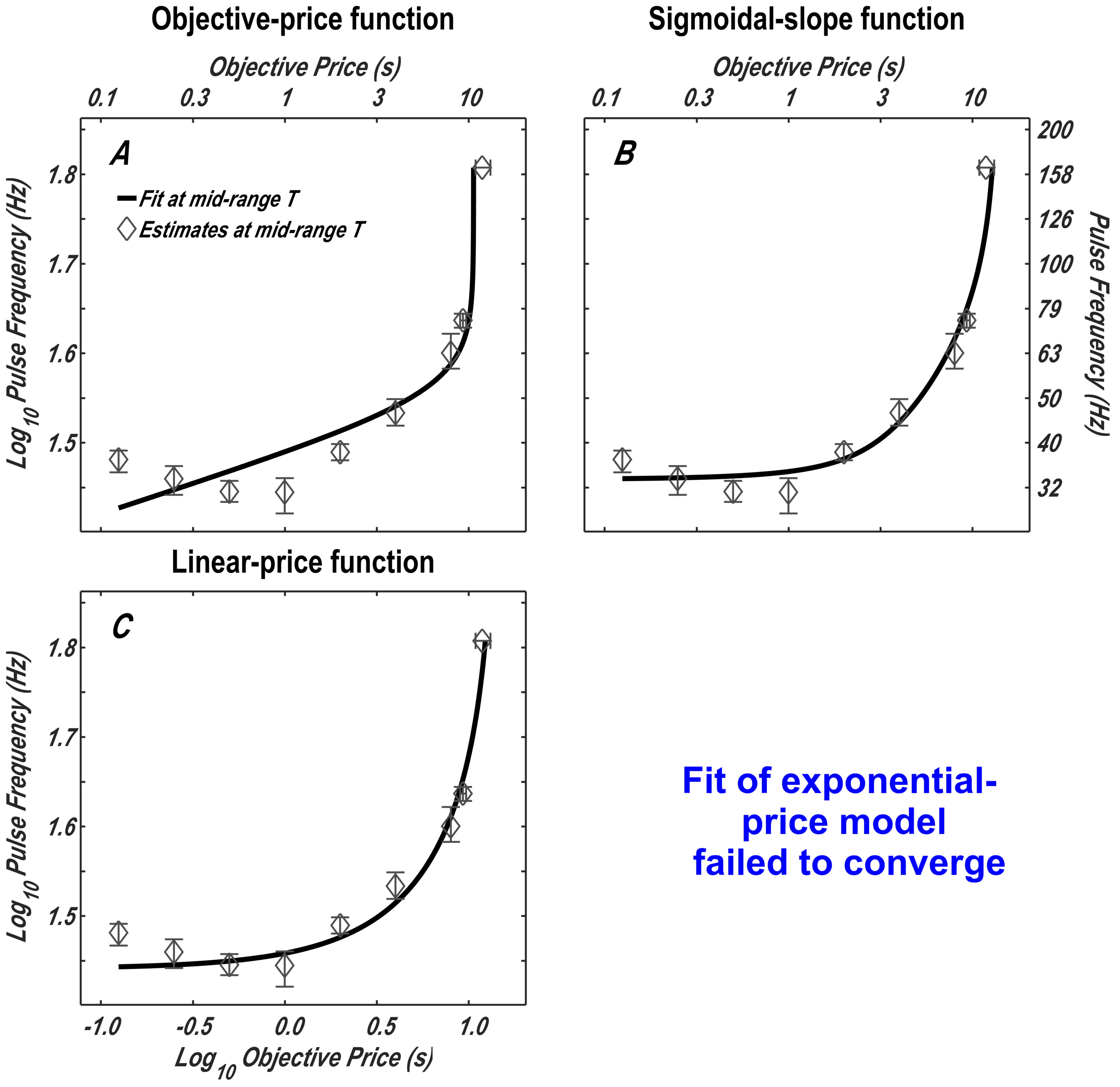

Fit of exponential-price model failed to converge

Figure R

Rat F03

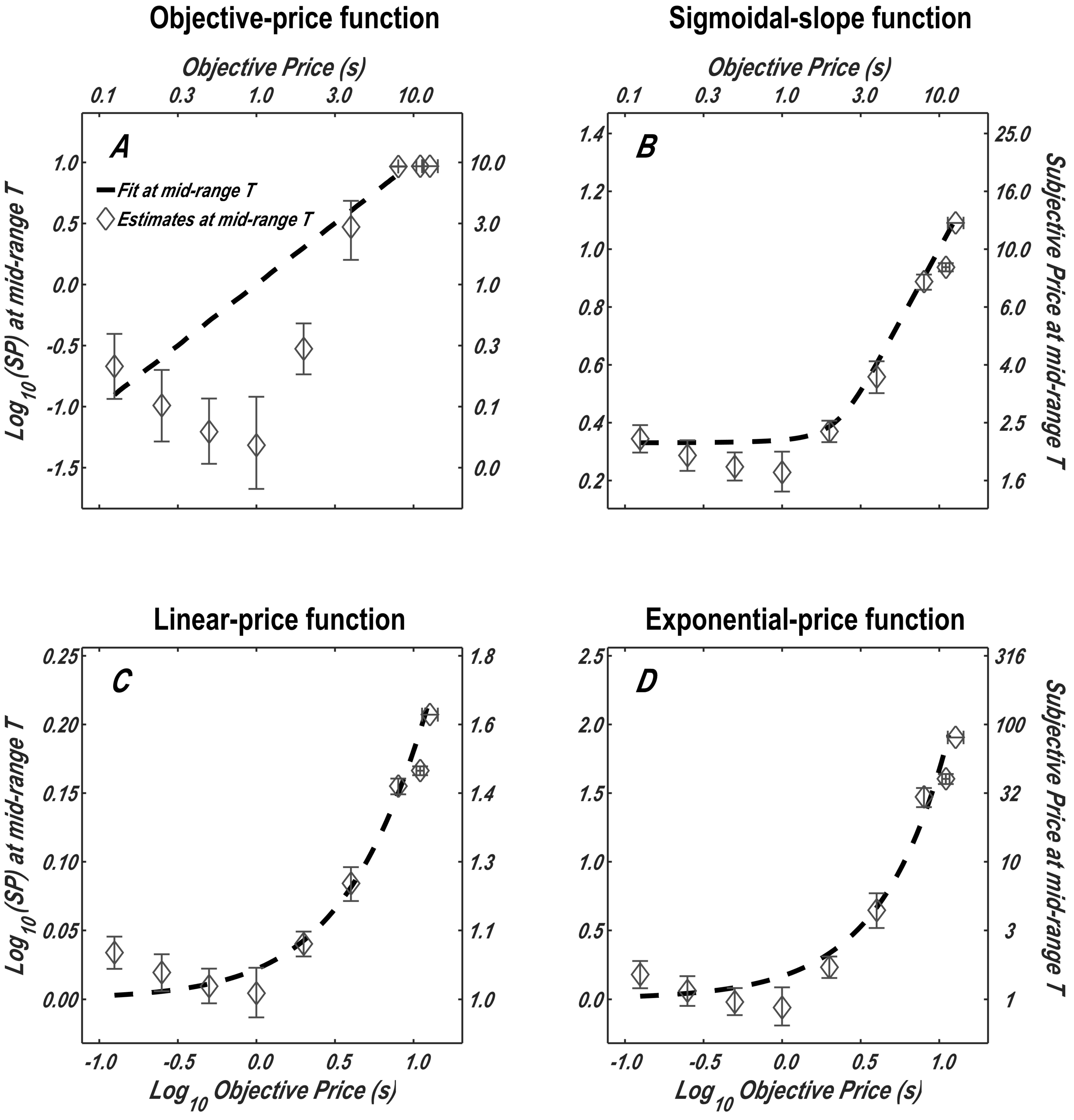

Figure S

Rat F09

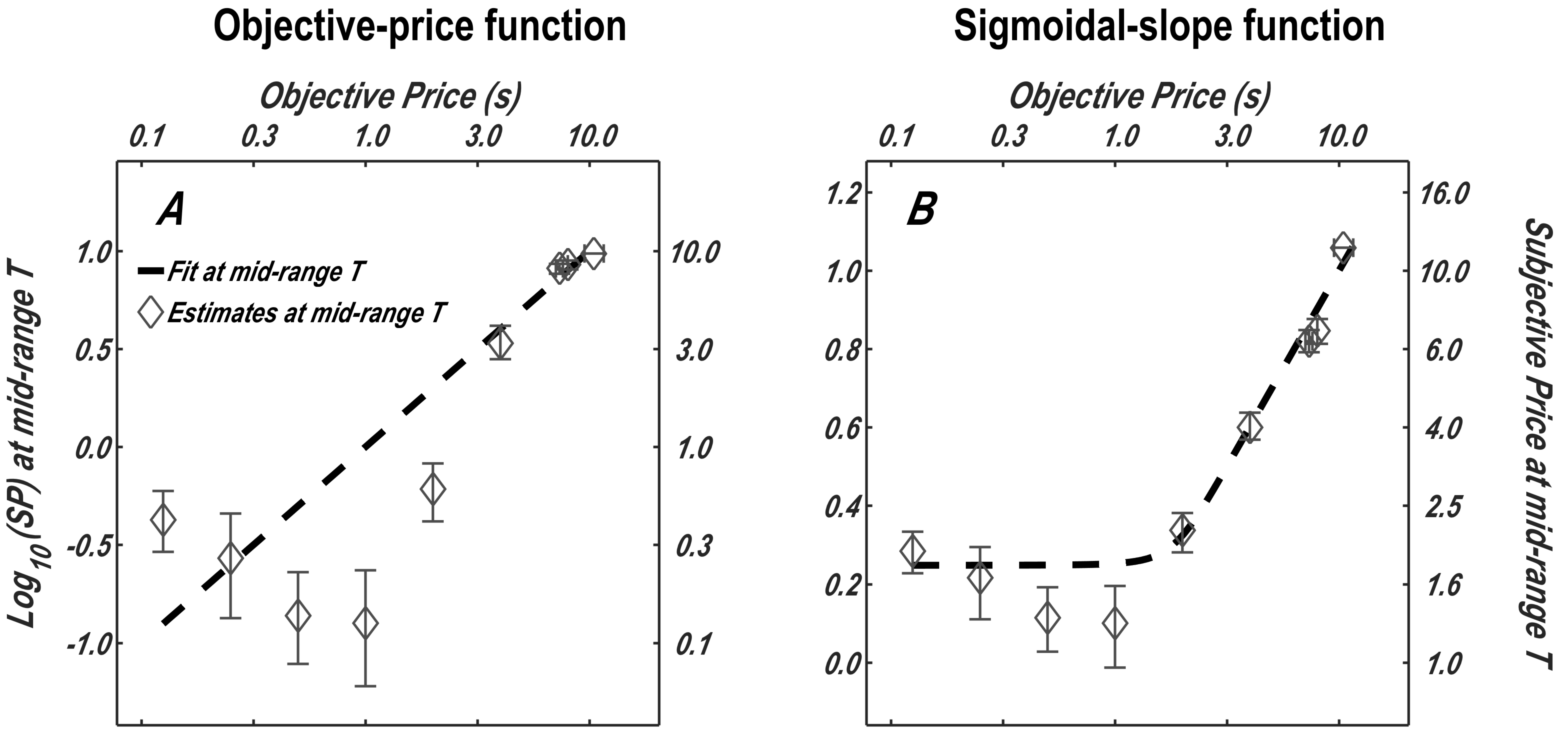

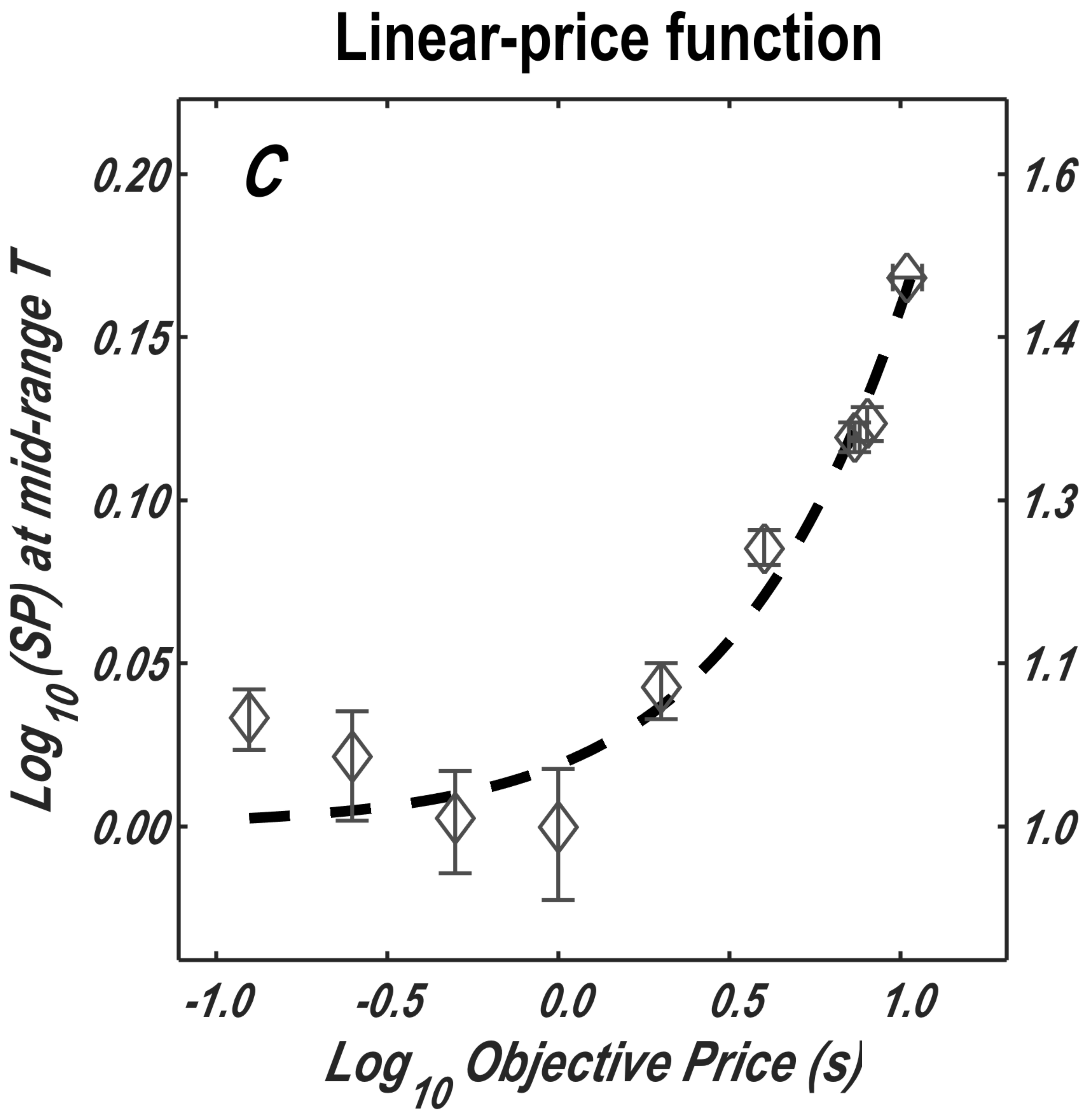

Fit of exponential-price model failed to converge

Figure T

Rat F12

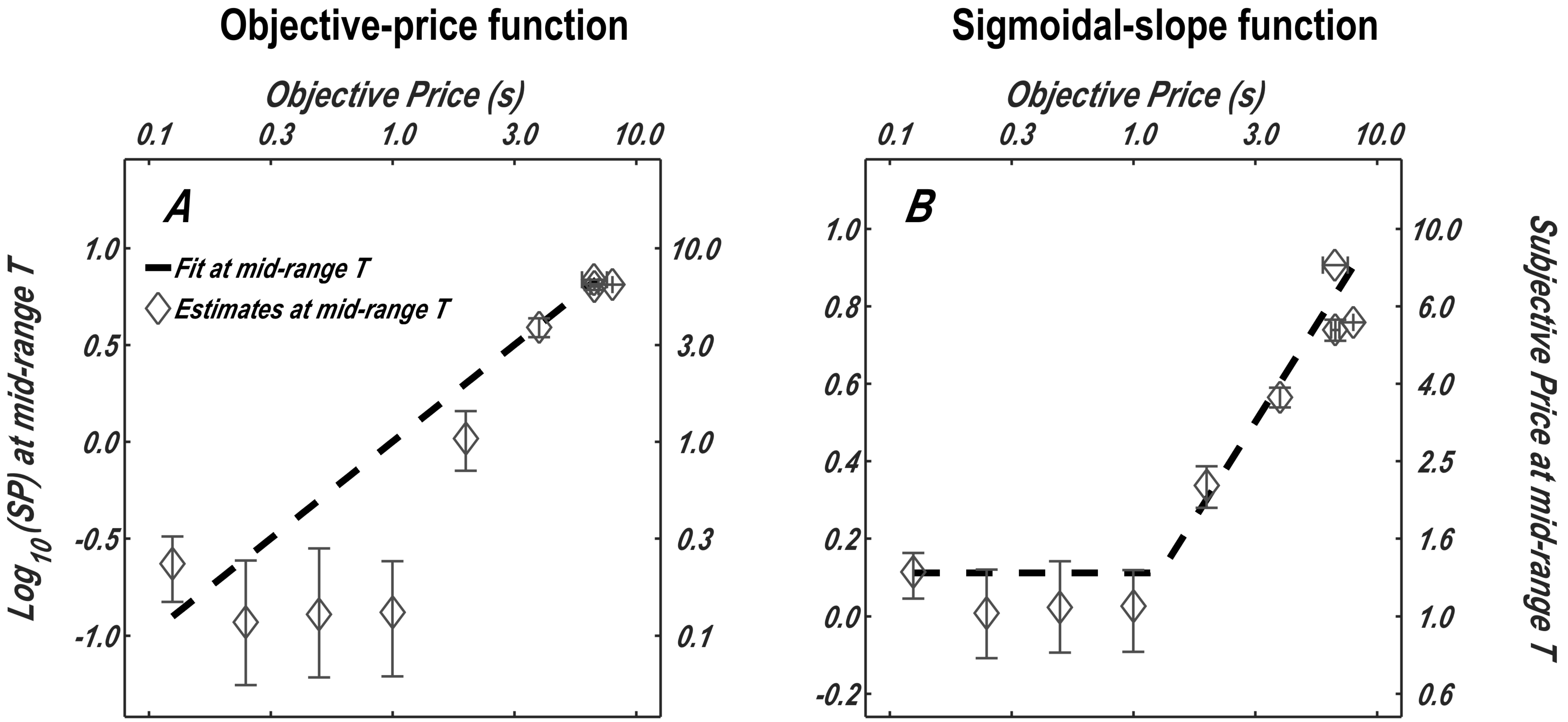

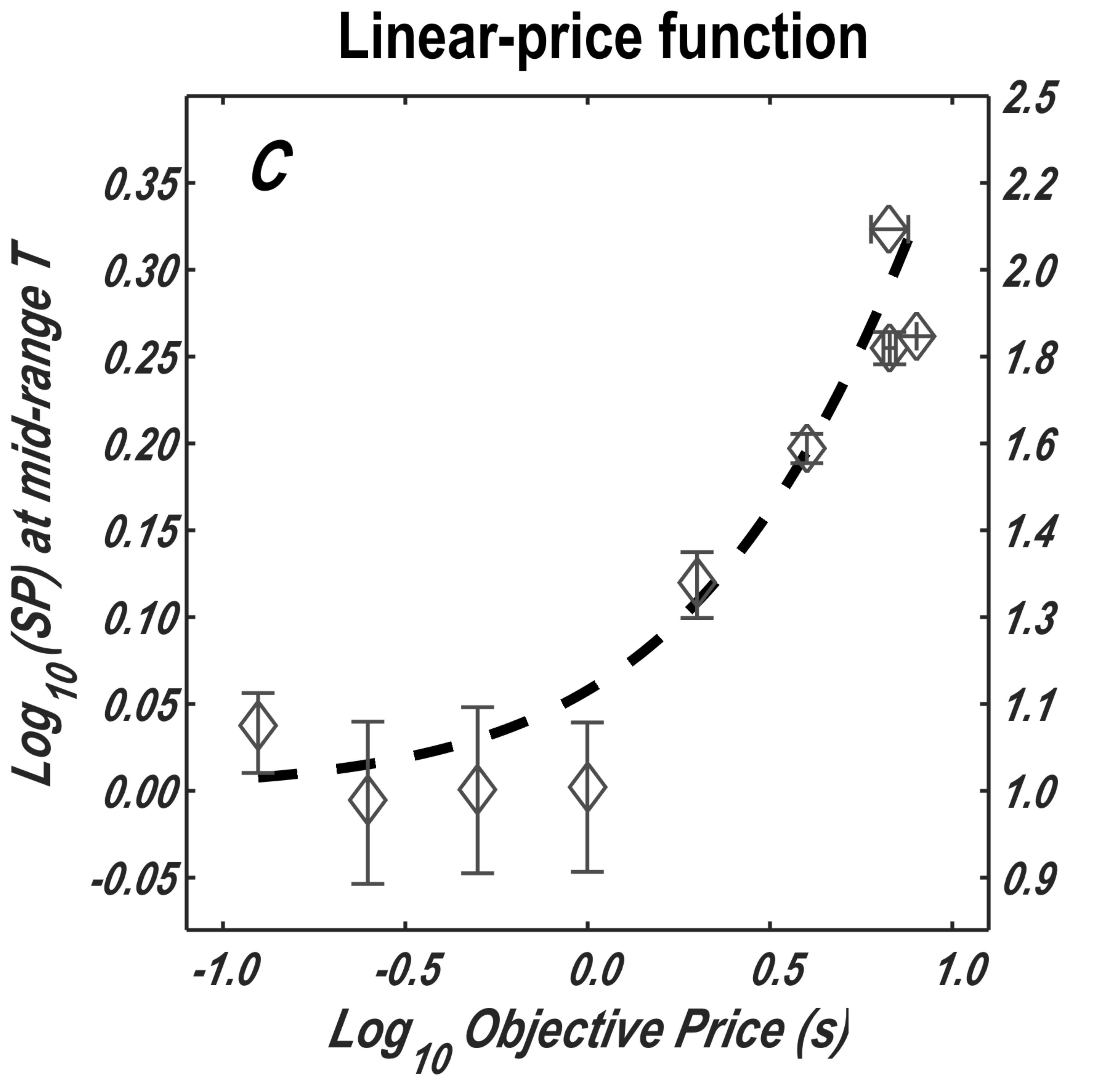

Fit of exponential-price model failed to converge

Figure U

Rat F16

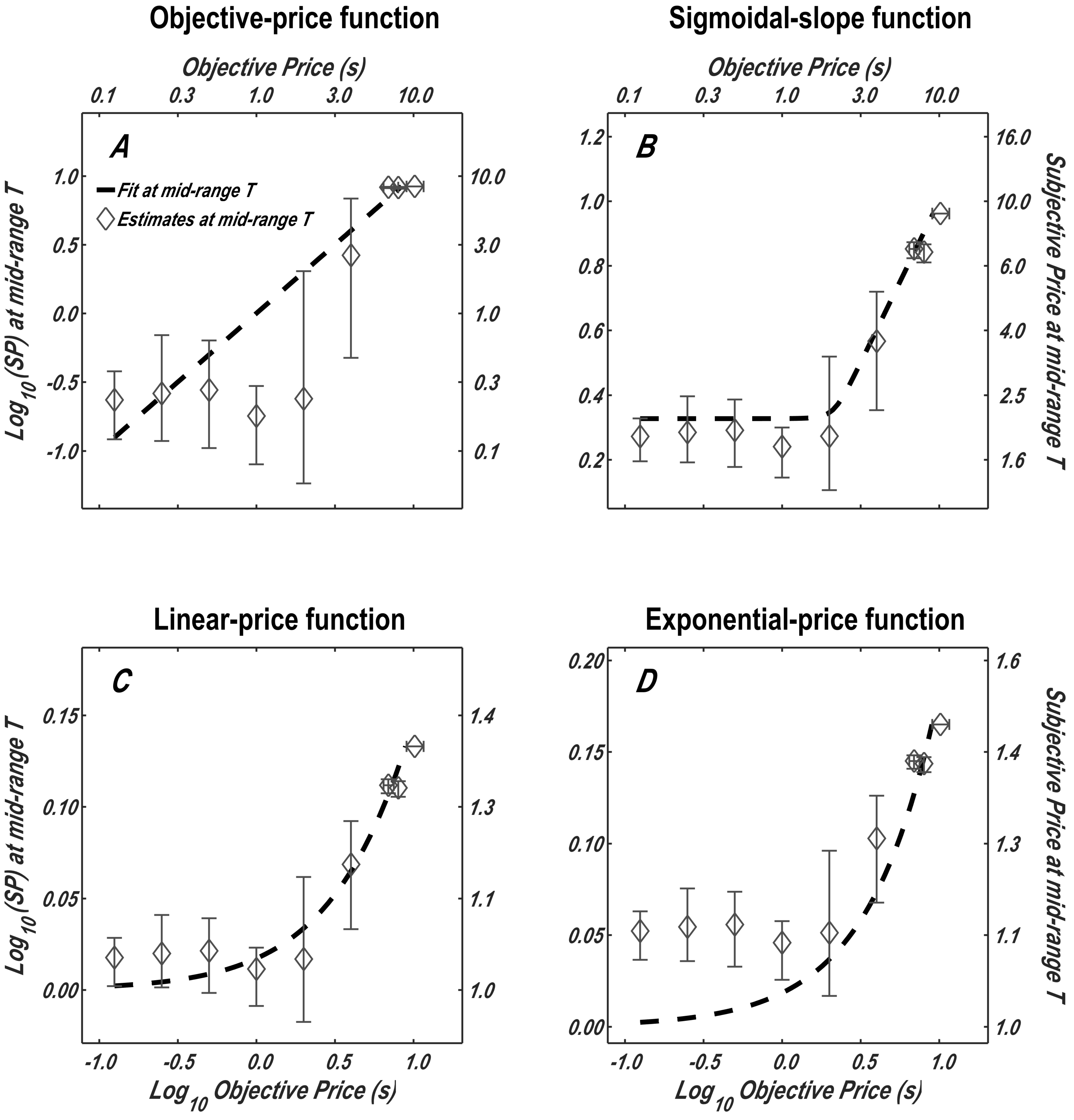

Figure V

Rat F17

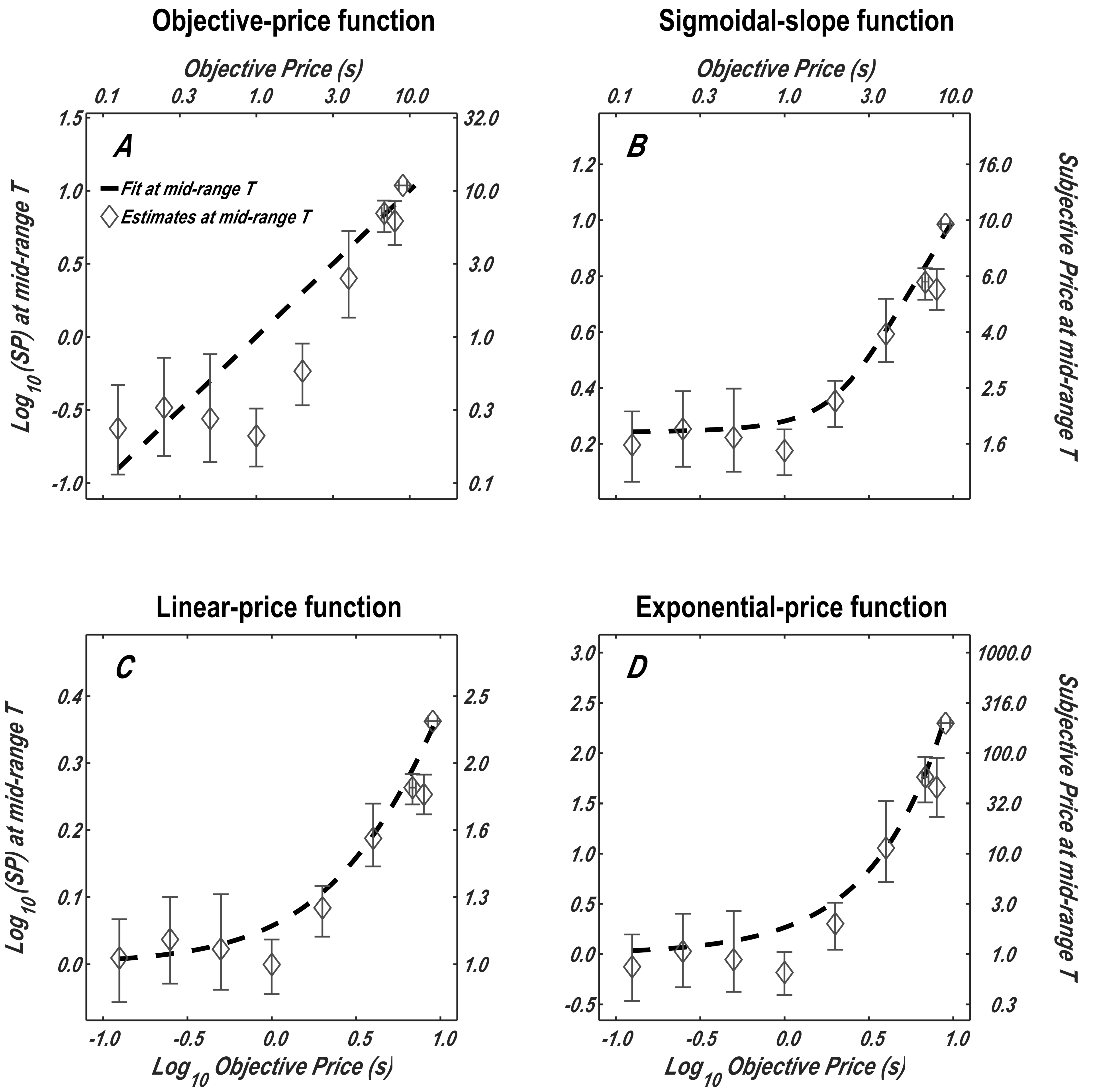

Figure W

Rat F18

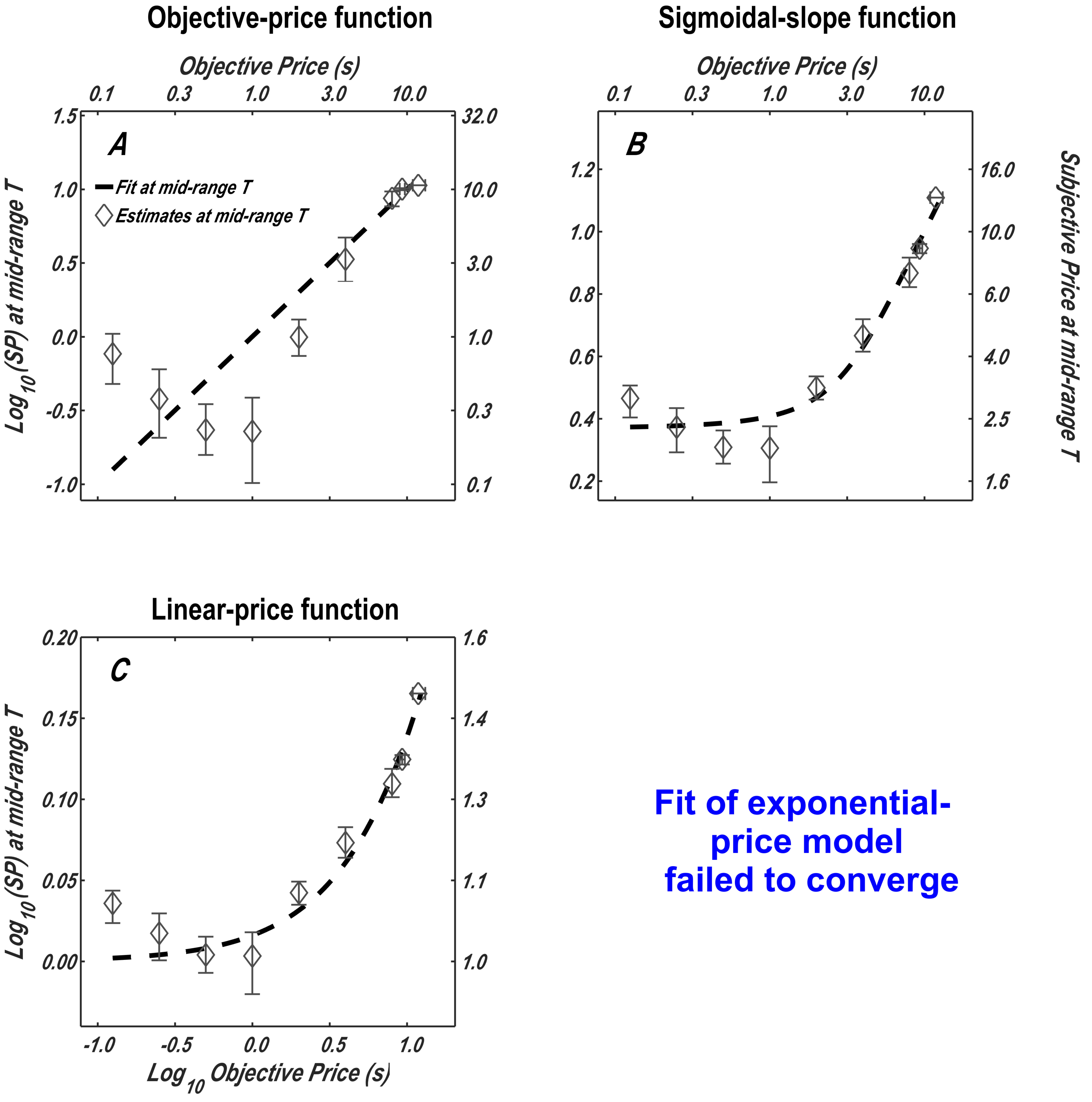

Figure X
